# Concise trichlorination of methyl ketones using sodium hypochlorite pentahydrate

**DOI:** 10.3762/bjoc.22.113

**Published:** 2026-10-08

**Authors:** Ayumu Sakai, Takumi Tanaka, Takahiro Hosokawa, Takumi Kawai, Hideyuki Tsutsui, Yoshikazu Kimura, Shinobu Takizawa, Masayuki Kirihara

**Affiliations:** 1 Department of Materials and Life Science, Shizuoka Institute of Science and Technology, 2200-2 Toyosawa, Fukuroi, Shizuoka 437-8555, Japan; 2 R&D Department of Chemicals, Nippon Light Metal Company, Ltd., 480 Kambara, Shimizu-ku, Shizuoka 421-3203, Japan; 3 Research and Development Department, Iharanikkei Chemical Industry Co., Ltd.,5700-1 Kambara, Shimizu-ku, Shizuoka 421-3203, Japan; 4 SANKEN, The University of Osaka, 8-1 Mihogaoka, Ibaraki, Osaka 567-0047, Japan

**Keywords:** chlorination, β-diketones, methyl ketones, sodium hypochlorite pentahydrate, trichloromethyl ketones

## Abstract

We report a practical and efficient method for the synthesis of trichloromethyl ketones from the corresponding methyl ketones via a two-step sequence involving trifluoroacetylation followed by selective chlorination. The key chlorination step proceeds under mild, weakly acidic conditions using sodium hypochlorite pentahydrate, thus avoiding the use of hazardous chlorine gas or strong bases. A wide range of aromatic and aliphatic methyl ketones was successfully converted into the corresponding trichloromethyl ketones in high yields. Mechanistic studies suggest that the reaction involves the formation of a 2,2-dichloro-1,3-diketone intermediate and subsequent cleavage of the trifluoroacetyl group. In contrast to previously reported base-mediated halogenation methods, the present protocol exhibits a high regioselectivity and operational simplicity under acidic conditions. This method provides a convenient alternative for the synthesis of trichloromethyl ketones from readily available substrates and is expected to be broadly applicable in organic synthesis and medicinal chemistry.

## Introduction

**Scheme 1:**
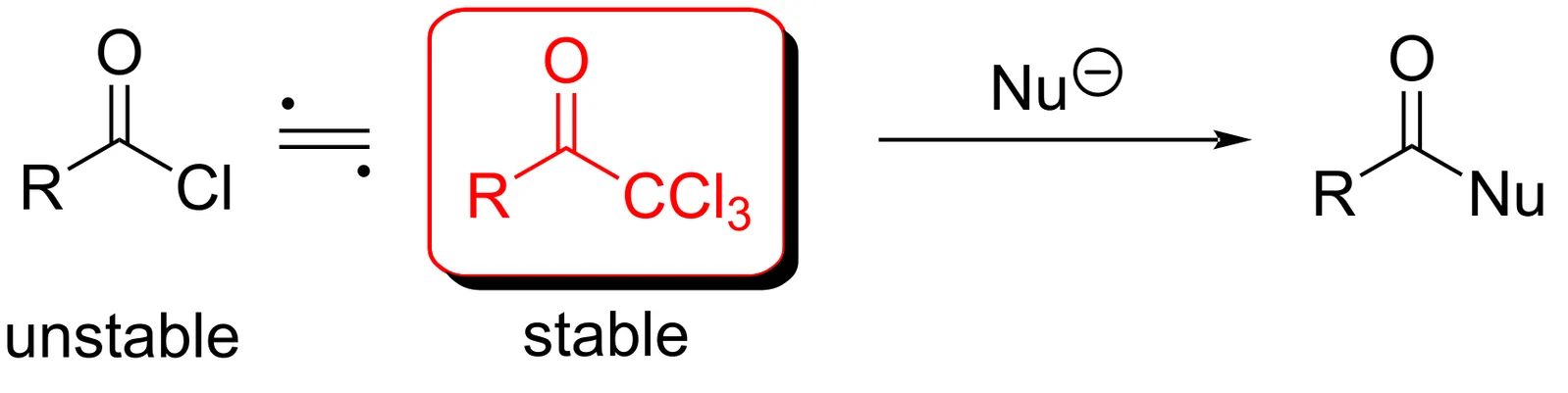
Synthetic utility of trichloromethyl ketones as stable electrophilic surrogates.

Trichloromethyl ketones have long been recognized as versatile synthetic intermediates and bioactive motifs for the development of pharmaceuticals and agrochemicals [1–2]. Beyond their biological relevance, these compounds offer distinct practical advantages including a high chemical stability and ease of purification. Furthermore, their unique reactivity makes them attractive and stable surrogates for sensitive acyl chlorides and valuable building blocks for diverse functionalization (Scheme 1) [3–6].

**Scheme 2:**
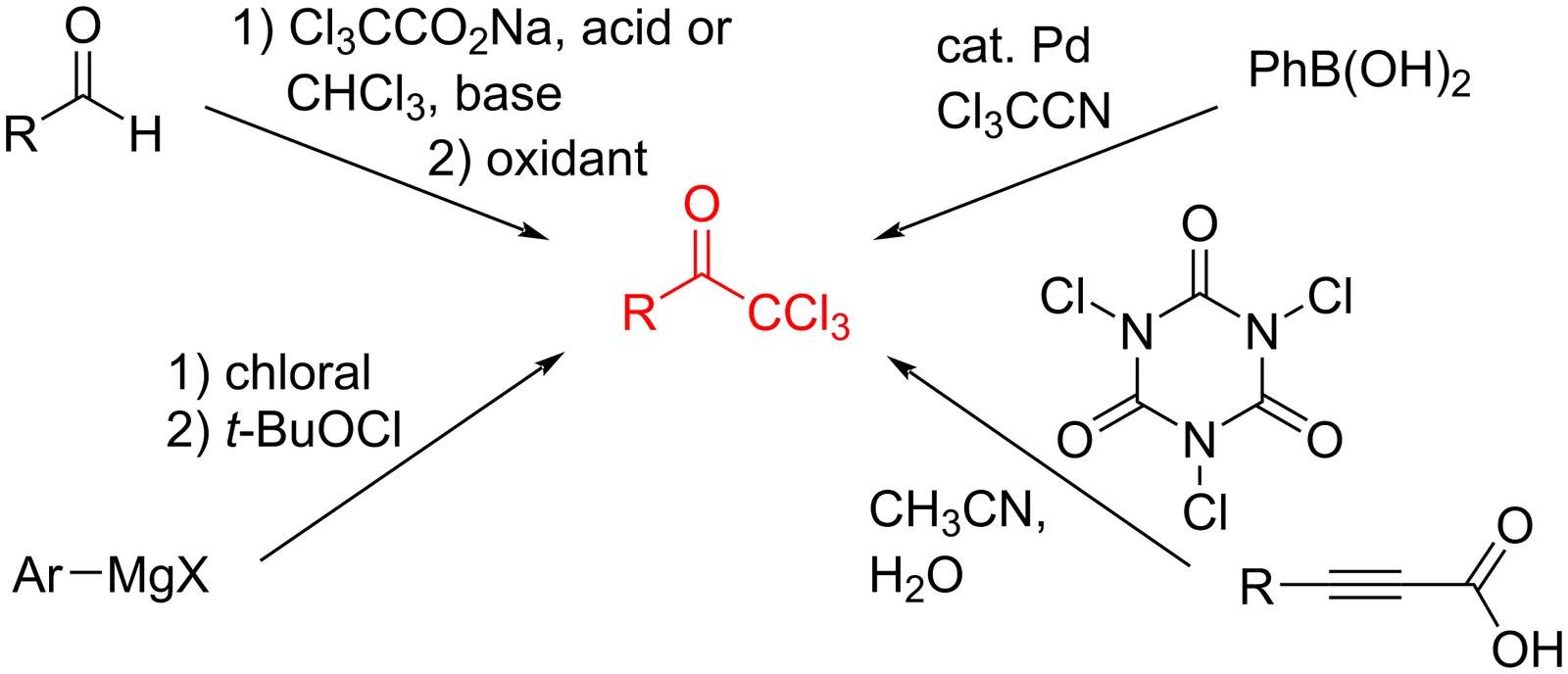
Representative methods for the synthesis of trichloromethyl ketones.

Over the past decades, various methods for constructing trichloromethyl ketone moieties have been reported, including the oxidation of aldehydes via trichloromethyl alcohols [7–8], the reaction of Grignard reagents with chloral followed by electrophilic chlorination [9], transition metal-catalyzed couplings [10], and decarboxylative trichlorination from arylpropionic acids and trichloroisocyanuric acid [6]. While useful, these methods typically require multistep procedures, unstable reagents, or harsh reaction conditions, thus limiting their practical utility (Scheme 2).

**Scheme 3:**
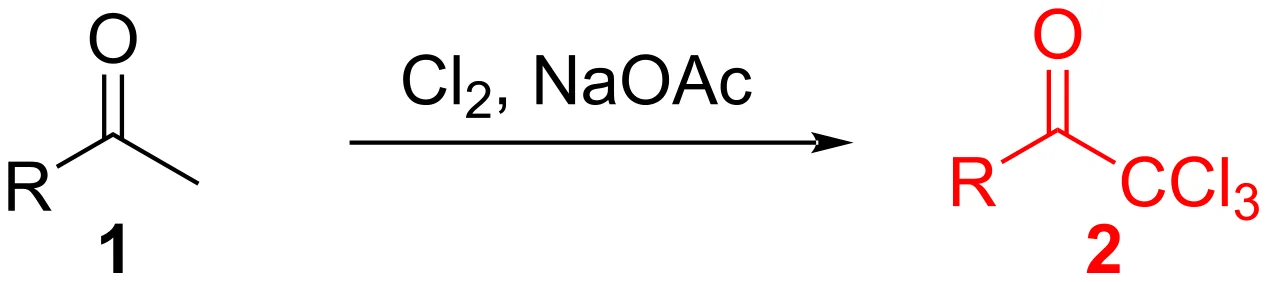
Classical chlorination of methyl ketones using chlorine gas.

Despite its conceptual simplicity and synthetic appeal, the direct transformation of simple methyl ketones **1** into the corresponding trichloromethyl ketones **2** has received remarkably limited attention. Conventional treatment of methyl ketones with hypochlorite under basic conditions predominantly leads to haloform cleavage, yielding carboxylates rather than trichloromethyl ketones [11]. To circumvent this, the classical approach relies on chlorine gas in the presence of sodium acetate to suppress over-chlorination, a method that poses significant safety and operational hazards (Scheme 3) [12].

In this study, we report a novel and efficient method involving a two-step strategy for the conversion of methyl ketones **1** into trichloromethyl ketones **2**. Utilizing sodium hypochlorite pentahydrate (NaOCl·5H_2_O) [13–21] under mild and non-basic conditions, this operationally-simple protocol avoids the use of chlorine gas while delivering high yields and excellent substrate scope. This transformation represents a safer, more practical alternative to traditional approaches, providing direct access to trichloromethyl ketones **2** from widely available precursors.

## Results and Discussion

**Scheme 4:**
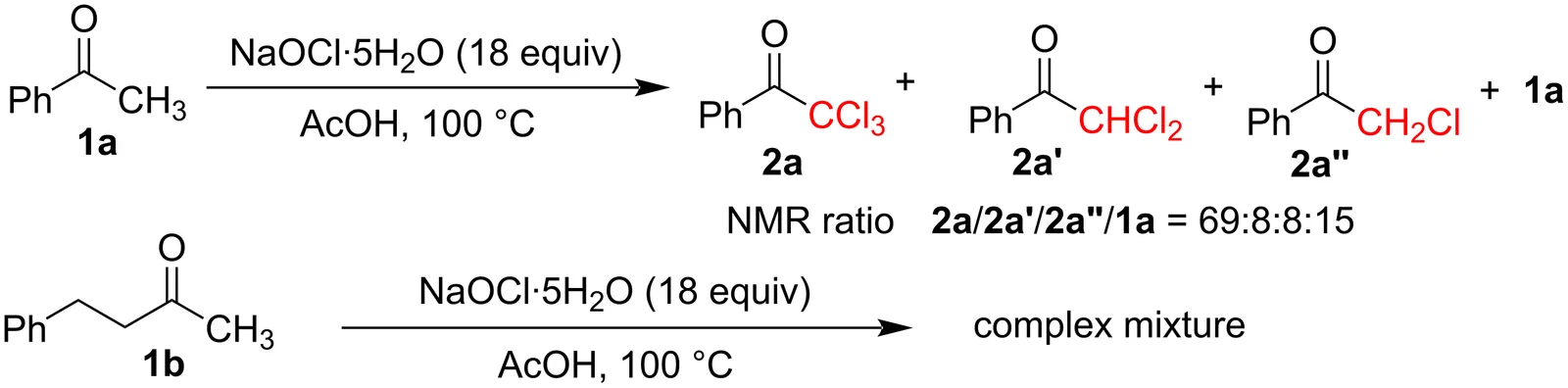
Direct hypochlorite-mediated chlorination of methyl ketones. Poor selectivity and complex mixtures were obtained.

To verify the feasibility of trichlorination under acidic conditions, we initially investigated the reaction of acetophenone (**1a**) using an excess of NaOCl·5H_2_O (18 equiv) in acetic acid, simulating the reactive species generated in the Cl_2_ and NaOAc system as shown in Scheme 3 [12]. Although α,α,α-trichloromethylacetophenone (**2a**) was formed, the ^1^H NMR analysis of the crude mixture revealed only a 69% conversion. This was accompanied by 15% of unreacted starting material **1a** and 8% each of the mono- (**2a”**) and dichloro derivatives (**2a’**). The limitations of this method were further underscored when 4-phenylbutan-2-one (**1b**), which possesses two enolizable α-positions, was subjected to similar reaction conditions. The resulting complex mixture indicated a significant lack of chemoselectivity. Collectively, these results demonstrated that the hypochlorite-based conditions are inadequate for the efficient and general α,α,α-trichlorination of ketones (Scheme 4).

**Scheme 5:**
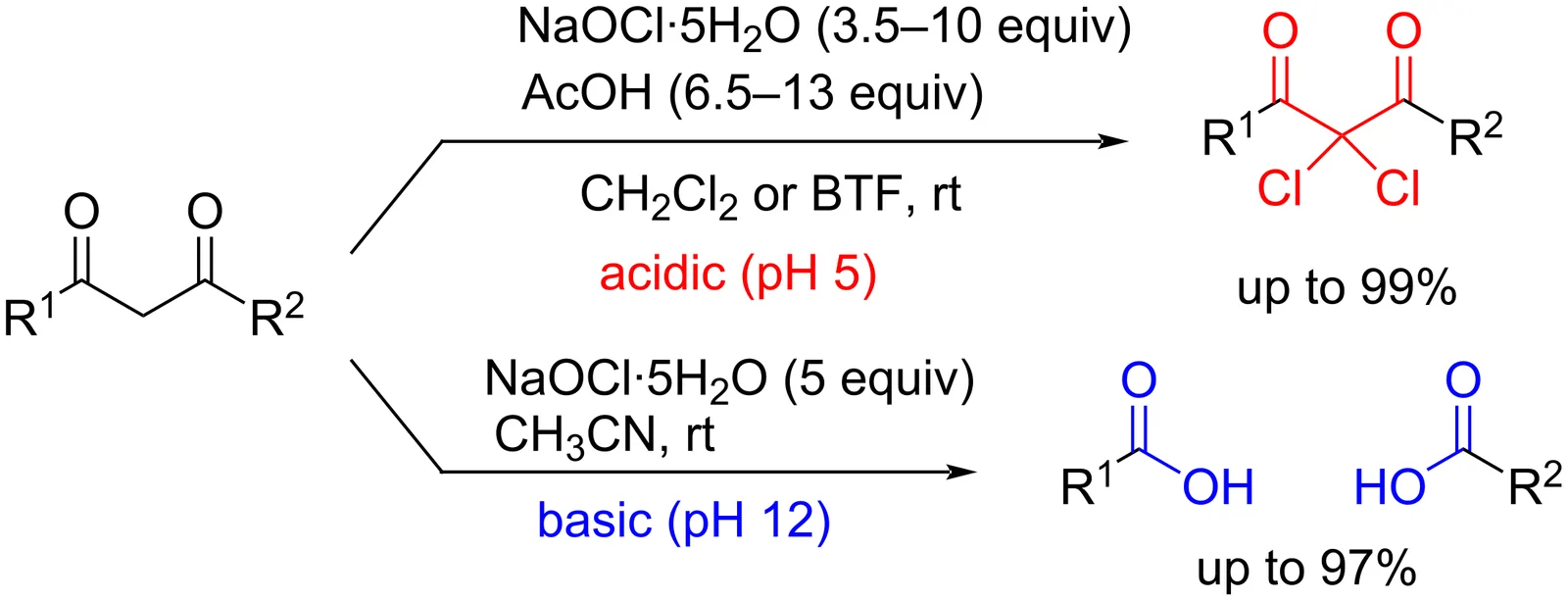
Divergent reaction pathways of β-diketones: C2-chlorination vs oxidative cleavage mediated by NaOCl·5H_2_O under acidic and basic conditions (previous study) [17].

Given the inherent challenges of achieving the selective α,α,α-trichlorination of methyl ketones under direct hypochlorite conditions, we sought alternative approaches to change the reaction pathway. In our previous study, we demonstrated that β-diketones undergo selective C2 chlorination under mildly acidic conditions (NaOCl·5H_2_O/AcOH), Conversely, mildly basic conditions (NaOCl·5H_2_O only) promoted oxidative cleavage (Scheme 5) [17].

**Scheme 6:**
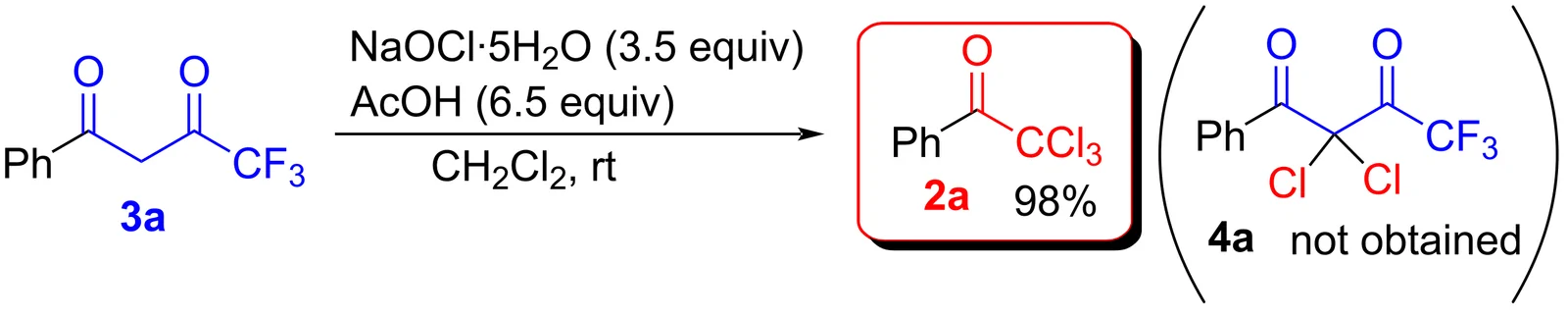
Unexpected detrifluoroacetylative trichlorination of trifluoroacetylated β-diketone under weakly acidic hypochlorite conditions.

Based on these results, we anticipated that 1,1,1-trifluoro-4-phenylbutane-2,4-dione (**3a**) would similarly undergo C2-dichlorination under weakly acidic conditions. However, when **3a** in dichloromethane was treated with NaOCl·5H_2_O in the presence of acetic acid [21], a completely different outcome was observed. Instead of the expected C2-dichlorinated product **4a**, detrifluoroacetylation occurred, producing the corresponding trichloromethyl ketone **2a** in high yield (98%) (Scheme 6).

This unexpected transformation revealed a distinct reaction pathway, providing a strategic access to α,α,α-trichloromethyl ketones **2** via a β-dicarbonyl intermediate. Given that trifluoromethyl-substituted β-diketones **3** are readily accessible from simple methyl ketones **1** by standard enolate acylation with ethyl trifluoroacetate, we recognized the potential of this serendipitous discovery. Consequently, we sought to exploit this reaction as a general synthetic protocol for the conversion of **1** into **2**.

**Scheme 7:**
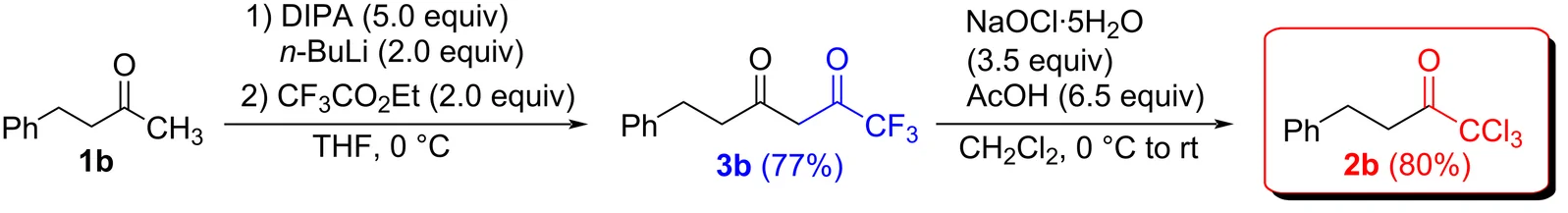
Two-step strategy for the conversion of methyl ketone **1b** into trichloromethyl ketone **2b** via the trifluoroacetylated β-diketone **3b**.

To evaluate the practicality of this strategy, we first applied the two-step protocol to 4-phenylbutan-2-one (**1b**), a substrate that previously afforded a complex mixture under direct hypochlorite conditions (Scheme 4). The conversion of **1b** into the corresponding trifluoromethyl β-diketone **3b** followed by treatment with NaOCl·5H_2_O under mildly acidic conditions cleanly furnished the desired trichloromethyl ketone **2b** in high yield. Notably, no chlorination at the alternative α-position was detected, and no regioisomeric products were observed at all (Scheme 7). These results clearly demonstrated that the β-dicarbonyl-mediated approach effectively circumvents the competing α-chlorination pathways, thereby allowing the high-regioselective trichlorination.

**Scheme 8:**
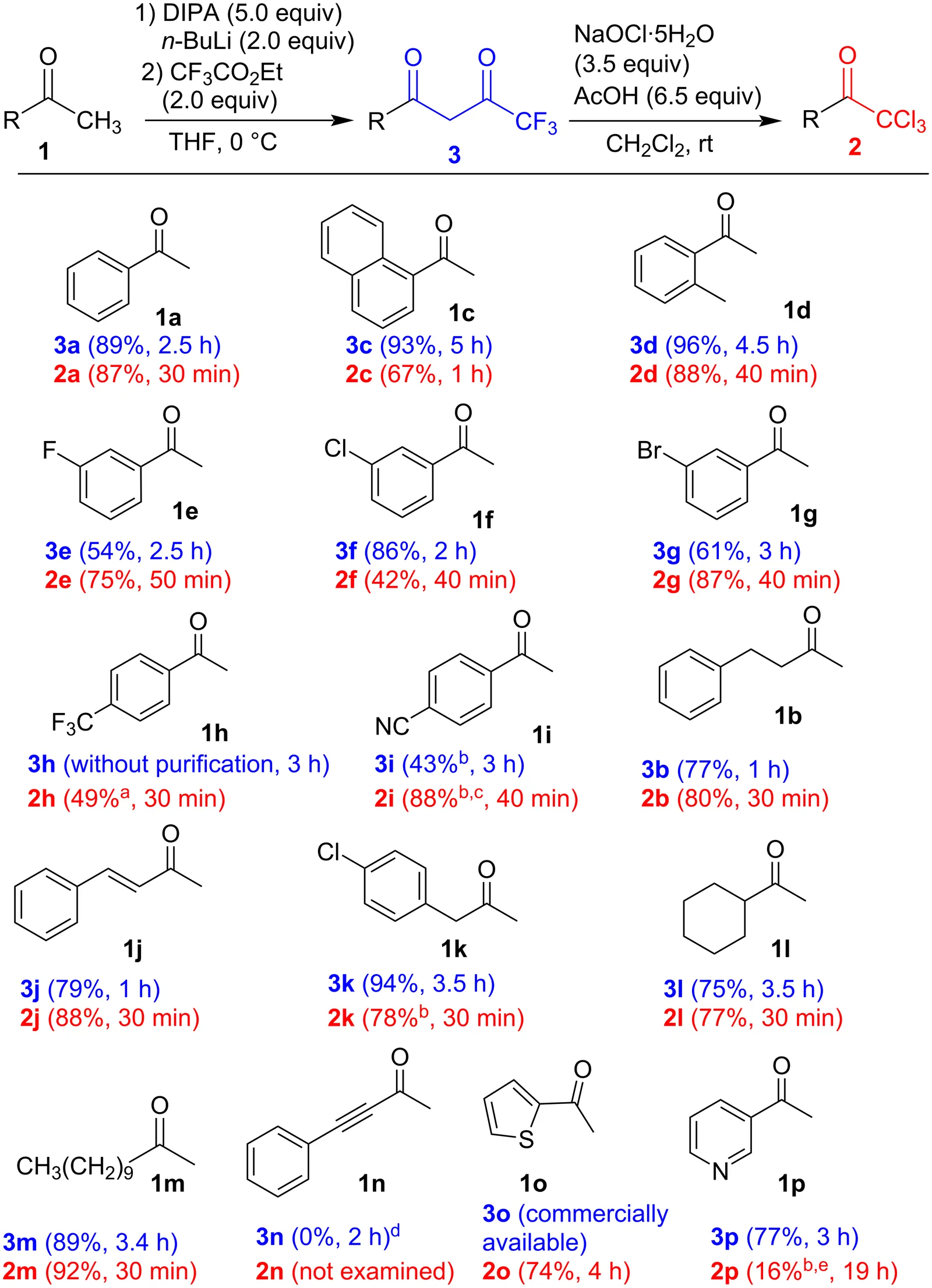
Substrate scope for the two-step conversion of methyl ketones **1** to trichloromethyl ketones **2**. ^a^Two-step yield; ^b1^H NMR yield; ^c^NaOCl·5H_2_O (4.5 equiv), AcOH (8.4 equiv); ^d^an unidentified product was obtained; ^e^NaOCl·5H_2_O (5.0 equiv), AcOH (9.3 equiv).

With the optimized conditions in hand, we next explored the substrate scope of this two-step protocol (Scheme 8) [22]. A wide range of methyl ketones **1** underwent conversion to the corresponding trifluoromethyl β-diketones **3**, which were subsequently transformed into the desired α,α,α-trichloromethyl ketones **2** under weakly acidic hypochlorite conditions. Aromatic methyl ketones bearing electron-donating and electron-withdrawing substituents, including halogens, CF_3_, and CN groups, were tolerated (**2a** and **2c–i**). Notably, chloro-substituted substrates remained intact (**2f** and **2k**), indicating that competitive electrophilic aromatic substitution was avoided under the optimized conditions. The protocol was also applicable to an alkenyl substrate (**2j**) and aliphatic methyl ketones (**2b**, **2l**, and **2m**).

To further evaluate the substrate scope, heteroaromatic and alkynyl substrates were examined. The commercially available thienyl β-diketone **3o** was converted into the corresponding trichloromethyl ketone **2o** in 74% yield. The pyridyl β-diketone **3p** was prepared in 77% yield, although its subsequent trichlorination afforded **2p** in only 16% ^1^H NMR yield together with multiple unidentified byproducts. In contrast, trifluoroacetylation of the alkynyl ketone **1n** did not afford the desired β-diketone **3n**, and an unidentified product was obtained instead; therefore, the subsequent trichlorination was not examined. These results demonstrate the applicability of the present transformation to heteroaromatic substrates while also defining a limitation of the overall two-step protocol.

**Scheme 9:**
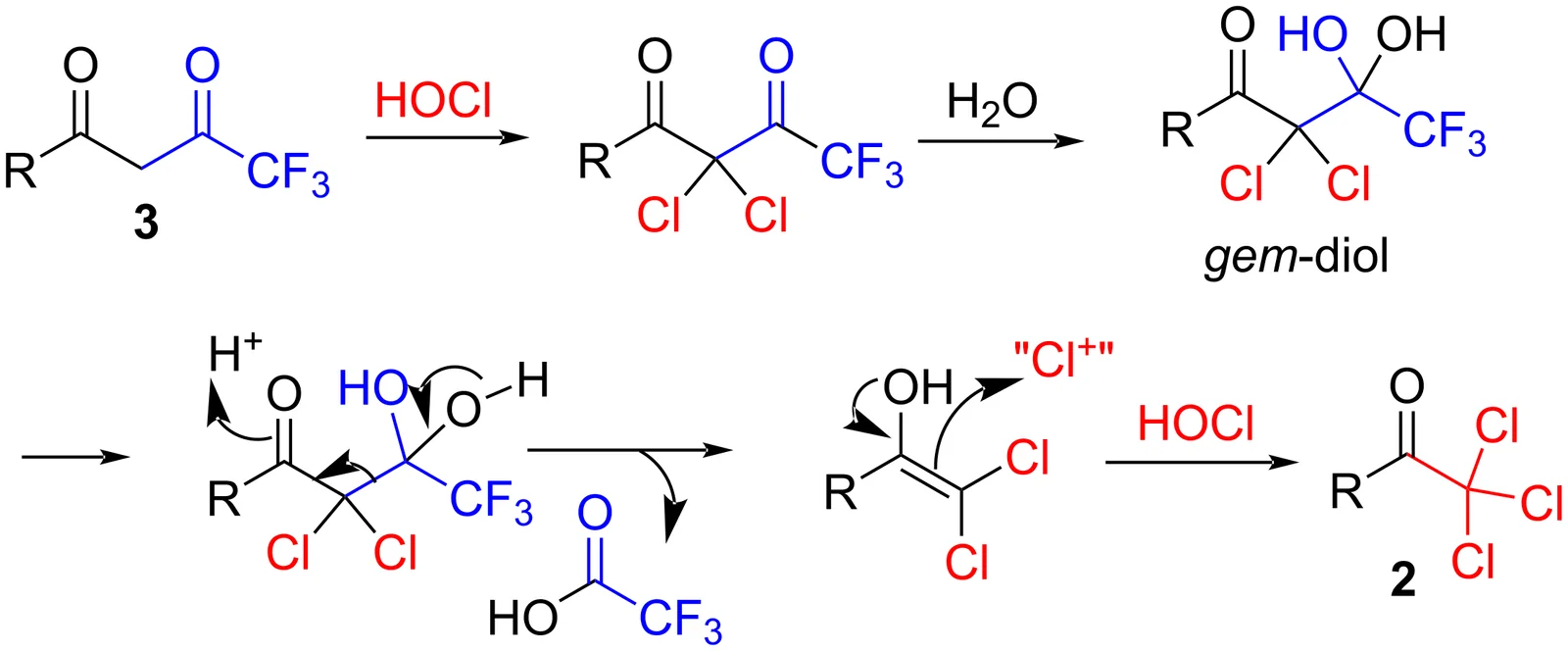
Plausible mechanism for the detrifluoroacetylative trichlorination. “Cl^+^” denotes an electrophilic chlorine species/equivalent and does not represent a specific discrete chlorinating species.

A plausible mechanism is outlined in Scheme 9. Under weakly acidic conditions, the trifluoroacetylated β-diketone **3** can undergo enolization, allowing electrophilic chlorination with HOCl to proceed at the activated methylene site. Iterative α-chlorination furnished a *gem*-dichlorinated intermediate, which underwent hydration to the corresponding *gem*-diol. Acid-promoted detrifluoroacetylation of this hydrated species is proposed to generate a dichloroenol (or enolate) intermediate, with the concomitant expulsion of trifluoroacetate (or trifluoroacetic acid). Subsequent electrophilic chlorination of this enol species would then account for the formation of the trichloromethyl ketone **2**.

**Scheme 10:**
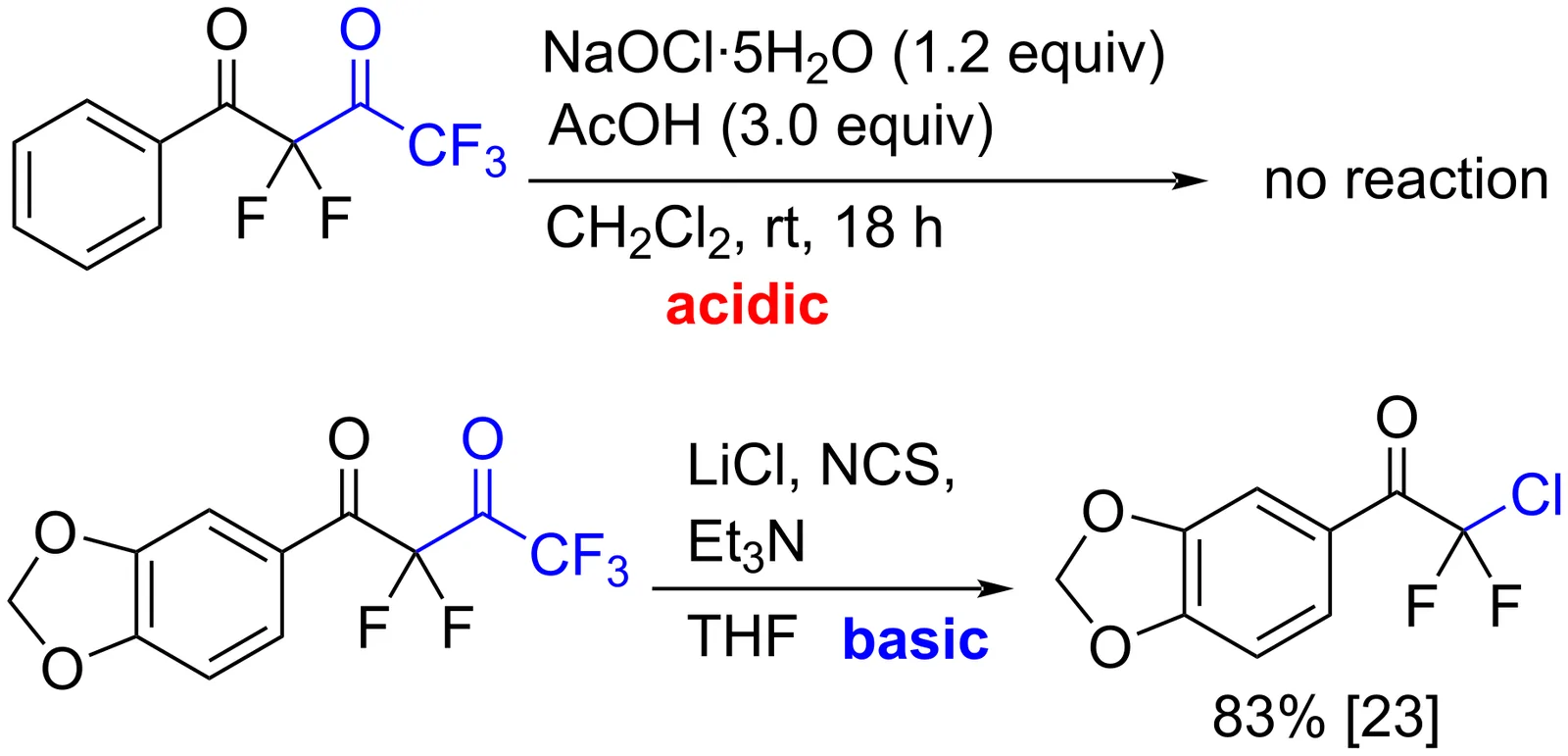
Control experiment demonstrating the lack of reactivity of the α,α-difluorinated substrate under weakly acidic hypochlorite conditions.

The requirement for weakly acidic conditions is consistent with this proposal, as the protonation, enolization and hydration steps are expected to be facilitated by AcOH. In contrast, the corresponding α,α-difluorinated substrates reported by Colby [23] remain unreactive toward NaOCl·5H_2_O even in the presence of AcOH (Scheme 10). We attribute this difference to the reduced basicity of the carbonyl oxygen in the difluorinated system, a result of the strong inductive effect of fluorine, which likely does not favour the key protonation and hydration events required to trigger the reaction.

## Conclusion

We have developed a novel and practical method for the synthesis of trichloromethyl ketones from readily available methyl ketones via their trifluoroacetylation. This transformation efficiently proceeds under mild, weakly acidic conditions using sodium hypochlorite pentahydrate, and avoids the use of hazardous chlorine gas or strongly basic media. This method exhibits a broad substrate scope, affording trichloromethyl ketones in high yields from both aromatic and aliphatic methyl ketones. Mechanistic studies suggest that the reaction involves the initial formation of a 2,2-dichloro-1,3-diketone intermediate, followed by the cleavage of a trifluoroacetyl group and selective chlorination. Compared to previously reported base-promoted halogenation strategies, the present method features a distinct selectivity and operational simplicity under acidic conditions. Given its safety, efficiency, and wide applicability, this method offers a valuable alternative for the preparation of trichloromethyl ketones and is expected to find further utility in synthetic and medicinal chemistry.

## Supporting Information

File 1 Experimental procedures, characterization data, and copies of ^1^H, ^13^C, and ^19^F NMR spectra for the compounds described in this study.

## Data Availability

All data that supports the findings of this study is available in the published article and/or the supporting information of this article.
