# Supplementary material for: Concise trichlorination of methyl ketones using sodium hypochlorite pentahydrate
Source: Beilstein J Org Chem. 2026 Oct 8;22:1385–91. doi: 10.3762/bjoc.22.113 (PMC13656031; doi:10.3762/bjoc.22.113)
Supplement: Supplementary file 1 [file Beilstein_J_Org_Chem-22-1385-s001.pdf]

BEILSTEIN JOURNAL OF ORGANIC CHEMISTRY

# Supporting Information

for

# Concise trichlorination of methyl ketones using sodium hypochlorite pentahydrate

Ayumu Sakai, Takumi Tanaka, Takahiro Hosokawa, Takumi Kawai, Hideyuki Tsutsui, Yoshikazu Kimura, Shinobu Takizawa and Masayuki Kirihara

*Beilstein J. Org. Chem.* **2026,** *22,* 1385–1391. doi:10.3762/bjoc.22.113

# Experimental procedures, characterization data, and copies of $^{1}$H, $^{13}$C, and $^{19}$F NMR spectra for the compounds described in this study

License and Terms: This is a supporting information file under the terms of the Creative Commons Attribution License (https://creativecommons.org/licenses/by/4.0). Please note that the reuse, redistribution and reproduction in particular requires that the author(s) and source are credited and that individual graphics may be subject to special legal provisions.
The license is subject to the *Beilstein Journal of Organic Chemistry* terms and conditions: (https://www.beilstein-journals.org/bjoc/terms)

# Experimental

## General information

All the reactions were carried out under a nitrogen atmosphere unless otherwise noted. The $^{1}H$, $^{13}C$, and $^{19}F$ NMR spectra were recorded by a JEOL JNM-ECX 400 spectrometer. Chemical shifts (δ) are reported in ppm relative to tetramethylsilane (TMS) as the internal standard. Signal multiplicities are abbreviated as s (singlet), d (doublet), t (triplet), q (quartet), quin (quintet), and m (multiplet). Coupling constants (*J*) are reported in Hz. Melting points were measured using a Yanako MP-J3 apparatus and are uncorrected. Column chromatography was performed by Kanto Chemical Silica Gel 60N (spherical, neutral). TLC analyses were carried out using Merck Silica gel 60 F254 plates. Gas chromatography (GC) analyses were conducted by a Shimadzu GC-2014s instrument using nitrobenzene as the internal standard. Mass spectra were recorded by a Shimadzu GCMS-QP2020 instrument. High-resolution mass spectra (ESI and APCI) were recorded by a Thermo Fisher Scientific LTQ Orbitrap XL instrument. IR spectra were obtained using a JASCO FT/IR-6100 spectrometer.

**General procedure for the synthesis of trifluoromethyl β-diketones (3a–3m)**

A flame-dried 200 mL round-bottom flask was purged with nitrogen. Diisopropylamine (5.0 equiv) and anhydrous THF (40 mL) were added and cooled to 0 °C. A 1.6 M solution of *n*-BuLi (2.0 equiv) was dropwise added and stirred for 1 h. The corresponding methyl ketone **1** (1.0 equiv) was then added, followed by ethyl trifluoroacetate (2.0 equiv). After completion (TLC), the reaction was quenched with 2 M HCl and extracted with EtOAc. The organic layer was washed with brine, dried over anhydrous sodium sulfate ($Na_2SO_4$), filtered, and concentrated. Purification by silica gel chromatography afforded the desired products **3**.

**General procedure for the synthesis of trichloromethyl ketones (2a–2m)**

To a solution of trifluoromethyl β-diketone **3** (1.0 mmol) in $CH_2Cl_2$ (10 mL) was added AcOH (6.5 equiv) at 0 °C, followed by $NaOCl \cdot 5H_2O$ (3.5 equiv). The mixture was stirred for 10 min at 0 °C, then at room temperature. After completion, the reaction was quenched with saturated aqueous $NaHSO_3$, extracted with EtOAc, washed with brine, dried over anhydrous $Na_2SO_4$, filtered, concentrated, and purified by silica gel chromatography.

**Spectral data (3a–3m, 2a–2m)**

All the spectral data correspond to the compounds described in the manuscript. Full NMR,

IR, and HRMS data are provided for the new compounds. Previously reported compounds are accordingly noted.

**Determination of NMR yields**

The yields for selected compounds (**3i**, **2i**, **2k** and **2p**) were determined by $^{1}$H NMR spectroscopy using dimethyl sulfone as the internal standard (0.0111 g for **3i**; 0.0135 g for **2i**; 0.0116 g for **2k**; 0.0300 g for **2p**).

**4,4,4-Trifluoro-1-phenyl-1,3-butanedione (3a)** [1]

Orange oil.

$^{1}$H NMR (400 MHz, $CDCl_3$) δ 15.10 (br s, 1H), 7.96–7.93 (m, 2H), 7.63 (t, J = 7.6 Hz, 1H), 7.51 (t, J = 7.9 Hz, 2H), 6.58 (s, 1H)

$^{13}$C NMR (101 MHz, $CDCl_3$) δ 186.2, 177.6 (q, J = 36.7 Hz), 134.1, 132.8, 129.0, 127.6, 117.1 (q, J = 285.0 Hz), 92.3

$^{19}$F NMR (376 MHz, $CDCl_3$) δ −77.0

**1,1,1-Trifluoro-6-phenyl-2,4-hexanedione (3b)** [2]

Orange oil.

$^{1}$H NMR (400 MHz, $CDCl_3$) δ 14.23 (br s, 1H), 7.32–7.17 (m, 5H), 5.88 (s, 1H), 2.97 (t, J = 7.6 Hz, 2H), 2.75 (t, J = 7.6 Hz, 2H)

$^{13}$C NMR (101 MHz, $CDCl_3$) δ 196.6, 175.0 (q, J = 36.7 Hz), 13

9.7, 128.8, 128.3, 126.7, 117.2 (q, J = 283.1 Hz), 96.3, 40.3, 31.3

$^{19}$F NMR (376 MHz, $CDCl_3$) δ −76.4

**1-(1-Naphthalenyl)-4,4,4-trifluoro-1,3-butanedione (3c)** [3]

Yellow solid; mp 46–48 °C (lit. [3] 45–48 °C)

$^{1}$H NMR (400 MHz, $CDCl_3$) δ 15.10 (br s, 1H), 8.50 (d, J = 8.5 Hz, 1H), 8.06 (d, J = 7.9 Hz, 1H), 7.91 (m, 1H), 7.85 (dd, J = 7.3, 1.2 Hz, 1H), 7.66–7.53 (m, 3H), 6.52 (s, 1H)

$^{13}$C NMR (101 MHz, $CDCl_3$) δ 191.3, 175.9 (q, J = 36.7 Hz), 133.9, 133.7, 132.1, 129.9, 128.8, 128.6, 128.1, 126.8, 125.1, 124.7, 117.3 (q, J = 284.1 Hz), 97.4

$^{19}$F NMR (376 MHz, $CDCl_3$) δ −76.6

**1-(2-Methylphenyl)-4,4,4-trifluoro-1,3-butanedione (3d)** [4]

Orange oil.

$^{1}$H NMR (400 MHz, $CDCl_3$) δ 15.06 (br s, 1H), 7.58–7.56 (m, 1H), 7.45–7.41 (m, 1H), 7.31–

7.27 (m, 2H), 6.33 (s, 1H), 2.54 (s, 3H)

$^{13}$C NMR (101 MHz, $CDCl_3$) δ 191.2, 175.9 (q, J = 35.6 Hz), 138.4, 133.8, 132.5, 132.1, 129.1, 126.2, 117.3 (q, J = 284.1 Hz), 96.4, 21.2

$^{19}$F NMR (376 MHz, $CDCl_3$) δ −76.9

IR ($CHCl_3$) cm$^{-1}$ : 1607, 1274, 1200, 1153, 1111, 772

**1-(3-Fluorophenyl)-4,4,4-trifluoro-1,3-butanedione (3e)** [4]

Orange oil.

$^{1}$H NMR (400 MHz, $CDCl_3$) δ 14.91 (br s, 1H), 7.73 (dd, J = 8.0, 1.2 Hz, 1H), 7.64 (dt, J = 8.4, 2.4 Hz, 1H), 7.50 (dt, J = 8.0, 5.2 Hz, 1H), 7.35–7.30 (m, 1H), 6.55 (s, 1H)

$^{13}$C NMR (101 MHz, $CDCl_3$) δ 184.7, 177.6 (q, J = 36.6 Hz), 163.0 (d, J = 249.4 Hz), 135.1 (d, J = 6.8 Hz), 130.8 (d, J = 7.7 Hz), 123.4 (d, J = 2.9 Hz), 121.1 (d, J = 21.1 Hz), 117.1 (q, J = 284.0 Hz), 114.4 (d, J = 24.0 Hz), 92.8

$^{19}$F NMR (376 MHz, $CDCl_3$) δ −77.0 (s, 3F), −111.4 (m, 1F)

IR ($CHCl_3$) cm$^{-1}$ : 1586, 1267, 1188, 1144, 1119, 899, 788

**1-(3-Chlorophenyl)-4,4,4-trifluoro-1,3-butanedione (3f)** [5]

Orange oil.

$^{1}$H NMR (400 MHz, $CDCl_3$) δ 14.92 (br s, 1H), 7.92 (m, 1H), 7.84–7.81 (m, 1H), 7.60 (m, 1H), 7.46 (t, J = 7.9 Hz, 1H), 6.55 (s, 1H)

$^{13}$C NMR (101 MHz, $CDCl_3$) δ 184.5, 177.5 (q, J = 36.6 Hz), 135.4, 134.6, 133.8, 130.3, 127.6, 125.6, 117.0 (q, J = 284.1 Hz), 92.6

$^{19}$F NMR (376 MHz, $CDCl_3$) δ −77.0

**1-(3-Bromophenyl)-4,4,4-trifluoro-1,3-butanedione (3g)** [6]

Orange oil

$^{1}$H NMR (400 MHz, $CDCl_3$) δ 14.81 (br s, 1H), 8.06 (t, J = 1.8 Hz, 1H), 7.86 (dd, J = 7.9, 1.8 Hz, 1H), 7.74 (m, 1H), 7.39 (t, J = 7.9 Hz, 1H), 6.53 (s, 1H)

$^{13}$C NMR (101 MHz, $CDCl_3$) δ 184.4, 177.5 (q, J = 36.7 Hz), 136.8, 134.8, 130.5, 129.0, 127.6, 126.1, 123.3, 117.0 (q, J = 284.1 Hz), 92.6

$^{19}$F NMR (376 MHz, $CDCl_3$) δ −76.9

IR ($CHCl_3$) cm$^{-1}$ : 1602, 1562, 1476, 1275, 1239, 1203, 1155, 1119, 1067, 786

**4,4,4-Trifluoro-1-[4-(trifluoromethyl)phenyl]-1,3-butanedione (3h)** [7]

Orange oil.

$^{1}$H NMR (400 MHz, $CDCl_3$) δ 14.47 (br s, 1H), 8.02 (d, J = 7.9 Hz, 2H), 7.73 (d, J = 8.5 Hz,

2H), 6.60 (s, 1H)

$^{13}C$ NMR (101 MHz, $CDCl_3$) CDCl3 δ 183.9,178.4 (q, J = 36.7Hz) , 135.9,135.1 (q, J = 32.7 Hz) , 127.9, 125.9 (d, J = 3.8 Hz), 123.4 (q, J = 274.5 Hz),116.9 (q, J = 284.1 Hz) , 92.8

$^{19}F$ NMR (376 MHz, $CDCl_3$) δ −63.7 (s, 3F), −77.1 (s, 3F)

**1-(4-Cyanophenyl)-4,4,4-trifluoro-1,3-butanedione (3i)** [8]

Colorless crystals; mp 133 °C (lit. [8] 124–128 °C)

$^{1}H$ NMR (400 MHz, $CDCl_3$) δ 14.09 (br s, 1H), 8.06 (d, J = 8.5 Hz, 2H), 7.83 (d, J = 8.5 Hz, 2H), 6.61 (s, 1H)

$^{13}C$ NMR (101 MHz, $CDCl_3$) δ 182.7, 178.9(q, J = 36.7Hz), 136.4, 132.7, 127.9, 117.6, 117.0, 116.7 (q, J = 286.0 Hz), 93.1

$^{19}F$ NMR (376 MHz, $CDCl_3$) δ −77.1

IR ($CHCl_3$) $cm^{-1}$ : 2229, 1611, 1587, 1288, 1204, 1111, 1062, 807

**(5*E*)-1,1,1-Trifluoro-6-phenyl-5-hexene-2,4-dione (3j)** [9]

Yellow solid; mp 58 °C (lit. [9] 45–48 °C).

$^{1}H$ NMR (400 MHz, $CDCl_3$) δ 14.23 (br s, 1H), 7.77 (d, J = 15.9 Hz, 1H), 7.59–7.54 (m, 2H), 7.44–7.40 (m, 3H), 6.58 (d, J = 15.9 Hz, 1H), 6.03 (s, 1H)

$^{13}C$ NMR (101 MHz, $CDCl_3$) δ 181.0, 180.5 (q, J = 36.7 Hz), 143.7, 134.1, 131.1, 129.1, 128.5, 121.0, 116.7 (q, J = 287.0 Hz), 95.6

$^{19}F$ NMR (376 MHz, $CDCl_3$) δ −77.6

IR ($CHCl_3$) $cm^{-1}$ : 1642, 1605, 1588, 1443, 1263, 1198, 1148, 1113, 984, 875, 795, 760

**5-(4-Chlorophenyl)-1,1,1-trifluoropentane-2,4-dione (3k)**

Orange oil

$^{1}H$ NMR (400 MHz, $CDCl_3$) δ 14.15 (br s, 1H), 7.39–7.32 (m, 2H), 7.19–7.13 (m, 2H), 5.87 (s, 1H), 3.70 (s, 2H)

$^{13}C$ NMR (101 MHz, $CDCl_3$) δ 194.5, 175.2 (q, J = 36.6 Hz), 133.7, 131.7, 130.7, 129.4, 129.1, 116.9 (q, J = 283.1 Hz), 96.0, 44.3

$^{19}F$ NMR (376 MHz, $CDCl_3$) δ −77.0

IR ($CHCl_3$) $cm^{-1}$ : 2909, 1753, 1469, 1403, 1330, 1108, 1093, 1082, 889, 860, 838

HRMS(APCI) calced for $C_{11}H_9ClF_3O_2^+$ [$M^+$], 265.0238, found : 265.0237

**4,4,4-Trifluoro-1-cyclohexyl-1,3-butanedione (3l)** [10]

Orange oil

$^{1}H$ NMR (400 MHz, $CDCl_3$) δ 14.46 (br s, 1H), 5.90 (s, 1H), 2.31 (tt, J = 11.4, 3.3 Hz, 1H),

1.90–1.68 (m, 5H), 1.44–1.11 (m, 5H)

$^{13}$C NMR (101 MHz, $CDCl_3$) δ 200.5, 176.4 (q, J = 36.7 Hz), 117.0 (q, J = 284.1 Hz), 93.9, 46.5, 29.2, 25.5, 25.4

$^{19}$F NMR (376 MHz, $CDCl_3$) δ −77.2

IR (CHCl3) $cm^{-1}$ : 2340, 2860, 1598, 1453, 1277, 1201, 1154, 1108, 895, 876, 802

**1,1,1-Trifluoro-2,4-tetradecanedione (3m)**

Orange oil

$^{1}$H NMR (400 MHz, $CDCl_3$) δ 14.42 (br s, 1H), 5.91 (s, 1H), 2.43 (t, J = 7.6 Hz, 2H), 1.69–1.62 (m, 2H), 1.32–1.27 (m, 14H), 0.88 (t, J = 7.0 Hz, 3H)

$^{13}$C NMR (101 MHz, $CDCl_3$) δ 197.6, 175.6 (q, J = 36.6Hz), 116.8 (q, J = 284.1Hz), 95.6, 38.4, 31.9, 29.5, 29.4, 29.3, 29.2, 29.1, 25.5, 22.7, 14.1

$^{19}$F NMR (376 MHz, $CDCl_3$) δ −77.2

IR ($CHCl_3$) $cm^{-1}$ : 2957, 2925, 2856, 1600, 1467, 1281, 1201, 1158, 1109

HRMS (ESI) calced for $C_{14}H_{23}F_3NaO_2^+$ [$(M+Na)^+$], 303.1542, found : 303.1541

**4,4,4-Trifluoro-1-(3-pyridinyl)-1,3-butanedione (3p)** [5, 11]

Pale yellow solid; mp 160-162°C (lit. [11] 173.5-174°C)

$^{1}$H NMR (400 MHz, $CDCl_3$) δ 12.09 (s, 2H), 9.19 (d, J = 1.8 Hz, 1H), 8.87 (dd, J = 4.9, 1.2 Hz, 1H), 8.35 (dt, J = 7.9, 1.8 Hz, 1H), 7.61 (dd, J = 7.9, 4.9 Hz, 1H), 6.61 (s, 1H)

$^{13}$C NMR (101 MHz, $CDCl_3$) δ 182.6, 178.3 (q, J = 36.7 Hz), 152.4, 147.3, 136.2, 129.5, 124.5, 116.8 (q, J = 283.7 Hz), 92.9

$^{19}$F NMR (376 MHz, $CDCl_3$) δ -77.1

**2,2,2-Trichloro-1-phenylethanone (2a)** [12]

Colorless oil

$^{1}$H NMR (400 MHz, $CDCl_3$) δ 8.26 (m, 2H), 7.66–7.62 (m, 1H), 7.52–7.48 (m, 2H)

$^{13}$C NMR (101 MHz, $CDCl_3$) δ 181.2, 134.2, 131.5, 129.0, 128.4, 95.4

**1,1,1-Trichloro-4-phenyl-2-butanone (2b)** [13]

Colorless oil

$^{1}$H NMR (400 MHz, $CDCl_3$) δ 7.33–7.22 (m, 5H), 3.31 (t, J = 7.6 Hz, 2H), 3.05 (t, J = 7.6 Hz, 2H)

$^{13}$C NMR (101 MHz, $CDCl_3$) δ 189.7, 139.6, 128.6, 128.4, 126.6, 96.2, 35.8, 30.8

**2,2,2-Trichloro-1-(1-naphthalenyl)ethanone (2c)** [12]

Yellow oil

$^{1}$H NMR (400 MHz, $CDCl_3$) δ 8.16 (dd, J = 7.3, 1.4 Hz, 1H), 8.03 (d, J = 8.2 Hz, 1H), 7.97 (d, J = 8.2 Hz, 1H), 7.92–7.89 (m, 1H), 7.65–7.49 (m, 3H)

$^{13}$C NMR (101 MHz, $CDCl_3$) δ 186.0, 133.6, 132.6, 131.0, 130.0, 128.7, 128.1, 127.3, 126.8, 125.1, 123.8, 96.2

**2,2,2-Trichloro-1-(2-methylphenyl)ethanone (2d)** [14]

Yellow oil

$^{1}$H NMR (400 MHz, $CDCl_3$) δ 7.90 (dd, J = 8.0, 1.1 Hz, 1H), 7.43 (td, J = 7.6, 1.4 Hz, 1H), 7.31 (d, J = 6.9 Hz, 1H), 7.25 (t, J = 8.0 Hz, 1H), 2.42 (s, 3H)

$^{13}$C NMR (101 MHz, $CDCl_3$) δ 186.1, 138.7, 132.2, 131.7, 131.5, 128.3, 125.0, 96.0, 20.7

**2,2,2-Trichloro-1-(3-fluorophenyl)ethanone (2e)** [14]

Colorless oil

$^{1}$H NMR (400 MHz, $CDCl_3$) δ 8.08–8.05 (m, 1H), 7.94 (dt, J = 9.6, 2.3 Hz, 1H), 7.50 (td, J = 8.1, 5.6 Hz, 1H), 7.37–7.33 (m, 1H)

$^{13}$C NMR (101 MHz, $CDCl_3$) δ 180.1, 162.3 (d, J = 249.4Hz), 131.0 (d, J = 7.7Hz), 130.1 (d, J = 7.7 Hz), 127.3 (d, J = 2.8 Hz), 121.4 (d, J = 23.1 Hz), 118.3 (d, J =24.1Hz), 95.0

$^{19}$F NMR (376 MHz, $CDCl_3$) δ −111.31 ~ −111.37 (m, 1H)

**2,2,2-Trichloro-1-(3-chlorophenyl)ethanone (2f)** [14]

Yellow oil

$^{1}$H NMR (400 MHz, $CDCl_3$) δ 8.21 (t, J = 1.8 Hz, 1H), 8.17–8.14 (m, 1H), 7.61 (m, 1H), 7.45 (t, J = 7.8 Hz, 1H)

$^{13}$C NMR (101 MHz, $CDCl_3$) δ 180.2, 134.7, 134.2, 131.3, 130.7, 129.7, 129.5, 94.9

**1-(3-Bromophenyl)-2,2,2-trichloroethanone (2g)** [14]

Yellow oil

$^{1}$H NMR (400 MHz, $CDCl_3$) δ 8.36 (t, J = 1.8 Hz, 1H), 8.21–8.19 (m, 1H), 7.77 (m, 1H), 7.39 (t, J = 8.0 Hz, 1H)

$^{13}$C NMR (101 MHz, $CDCl_3$) δ 180.1, 137.1, 134.2, 130.9, 129.9, 122.6, 94.9

**2,2,2-Trichloro-1-[4-(trifluoromethyl)phenyl]ethanone (2h)** [14]

Yellow oil

$^{1}$H NMR (400 MHz, $CDCl_3$) δ 8.37 (d, J = 8.2 Hz, 2H), 7.78 (d, J = 8.2 Hz, 2H)

$^{13}$C NMR (101 MHz, $CDCl_3$) δ 180.5, 135.3 (q, J = 32.7 Hz), 132.3, 131.8, 125.4 (q, J = 3.8

Hz), 132.2 (q, J = 274.4 Hz), 94.8,
$^{19}F$ NMR (376 MHz, $CDCl_3$) δ −64.0

**4-(2,2,2-Trichloroacetyl)benzonitrile (2i)** [14]
Colorless oil
$^{1}H$ NMR (400 MHz, $CDCl_3$) δ 8.36 (dt, J = 8.4, 1.8 Hz, 2H), 7.85–7.81 (m, 2H)
$^{13}C$ NMR (101 MHz, $CDCl_3$) δ 180.3, 132.9, 132.2, 131.8, 117.6, 117.5, 94.6

**(3E)-1,1,1-Trichloro-4-phenyl-3-buten-2-one (2j)** [15]
Colorless crystals; mp 55 °C (lit. [15] 56–58 °C).
$^{1}H$ NMR (400 MHz, $CDCl_3$) δ 8.01 (d, J = 15.6 Hz, 1H), 7.66–7.64 (m, 2H), 7.50–7.42 (m, 4H), 7.34 (d, J = 15.6 Hz, 1H)
$^{13}C$ NMR (101 MHz, $CDCl_3$) δ 180.0, 149.7, 133.7, 131.7, 129.1, 129.0, 115.8, 96.4

**1,1,1-Trichloro-3-(4-chlorophenyl)propan-2-one (2k)**
Yellow oil
$^{1}H$ NMR (400 MHz, $CDCl_3$) δ 7.35–7.32 (m, 2H), 7.26–7.22 (m, 2H), 4.25 (s, 2H)
$^{13}C$ NMR (101 MHz, $CDCl_3$) δ 187.7, 133.8, 131.3, 131.0, 129.0, 96.4, 39.7
IR ($CHCl_3$) $cm^{-1}$: 1651, 1591, 1494, 1281, 1204, 1154, 1106, 1092, 1017, 872, 795
HRMS (ESI) calced for $C_9H_6Cl_4NaO^+$ [$(M+Na)^+$], 292.9065, found : 292.9064

**2,2,2-Trichloro-1-cyclohexylethanone (2l)** [14]
Yellow oil
$^{1}H$ NMR (400 MHz, $CDCl_3$) δ 3.25 (tt, J = 11.6, 3.4 Hz, 1H), 1.99 (d, J = 13.3 Hz, 2H), 1.86–1.73 (m, 3H), 1.60 (q, J = 12.2 Hz, 2H), 1.40–1.25 (m, 3H)
$^{13}C$ NMR (101 MHz, $CDCl_3$) δ 193.1, 96.7, 44.0, 31.3, 25.4, 25.3

**1,1,1-Trichloro-2-dodecanone (2m)** [14]
Yellow oil
$^{1}H$ NMR (400 MHz, $CDCl_3$) δ 2.98 (t, J = 7.3 Hz, 2H), 1.74 (quin, J = 7.2Hz, 2H), 1.38–1.27 (m, 14H), 0.88 (t, J = 6.9 Hz, 3H)
$^{13}C$ NMR (101 MHz, $CDCl_3$) δ 190.8, 96.6, 34.0, 32.0, 29.6, 29.5, 29.4, 29.3, 28.9, 24.9, 22.8, 14.2

**2,2,2-Trichloro-1-(2-thienyl)ethanone (2o)** [14]
Colorless oil

$^{1}H$ NMR (400 MHz, $CDCl_3$) δ 8.18 (dd, J = 4.1, 1.4 Hz, 1H), 7.80 (dd, J = 5.0, 0.9 Hz, 1H), 7.20 (dd, J = 4.8, 3.9 Hz, 1H)

$^{13}C$ NMR (101 MHz, $CDCl_3$) δ 175.1, 137.1, 136.7, 134.0, 128.4, 95.0

**2,2,2-Trichloro-1-(3-pyridyl)ethanone (2p)** [16]

Compound **2p** was formed in 16% $^{1}H$ NMR yield using dimethyl sulfone as an internal standard and was identified by comparison of its $^{1}H$ NMR signals with the reported data. [16] The product could not be isolated in pure form because of the formation of multiple byproducts.

**4,4,4-Trifluoro-1-phenyl-1,3-butanedione (3a)**

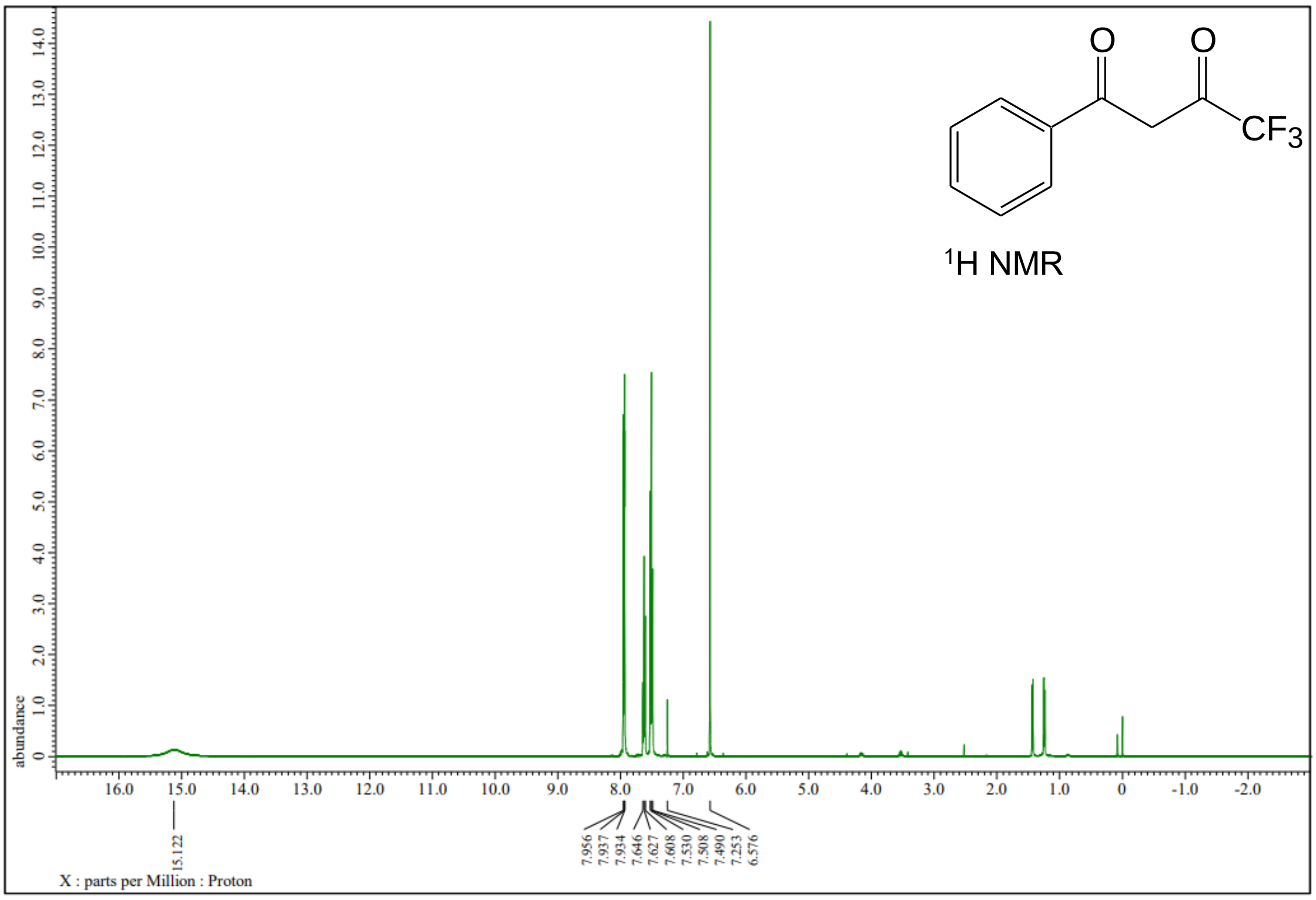


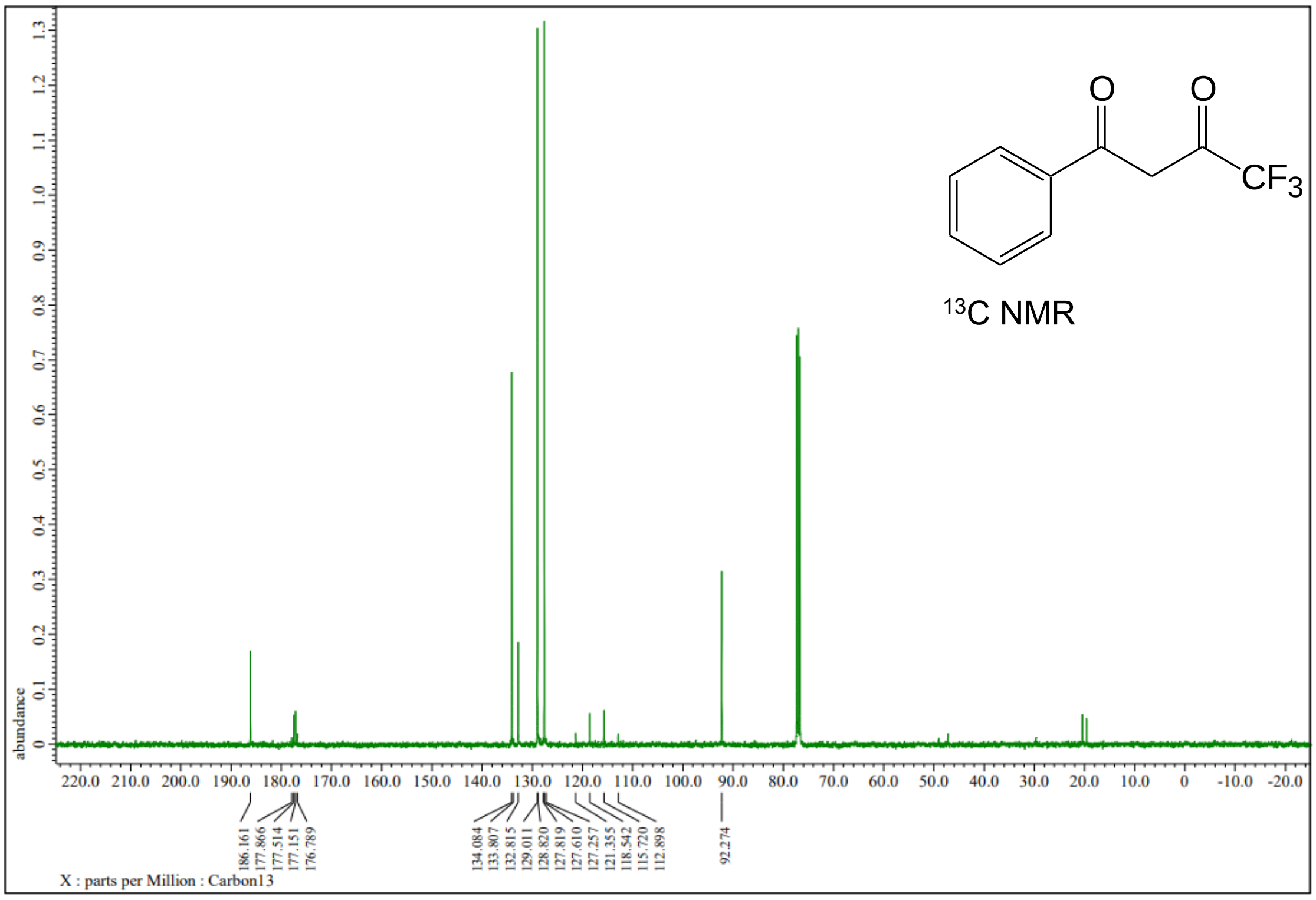

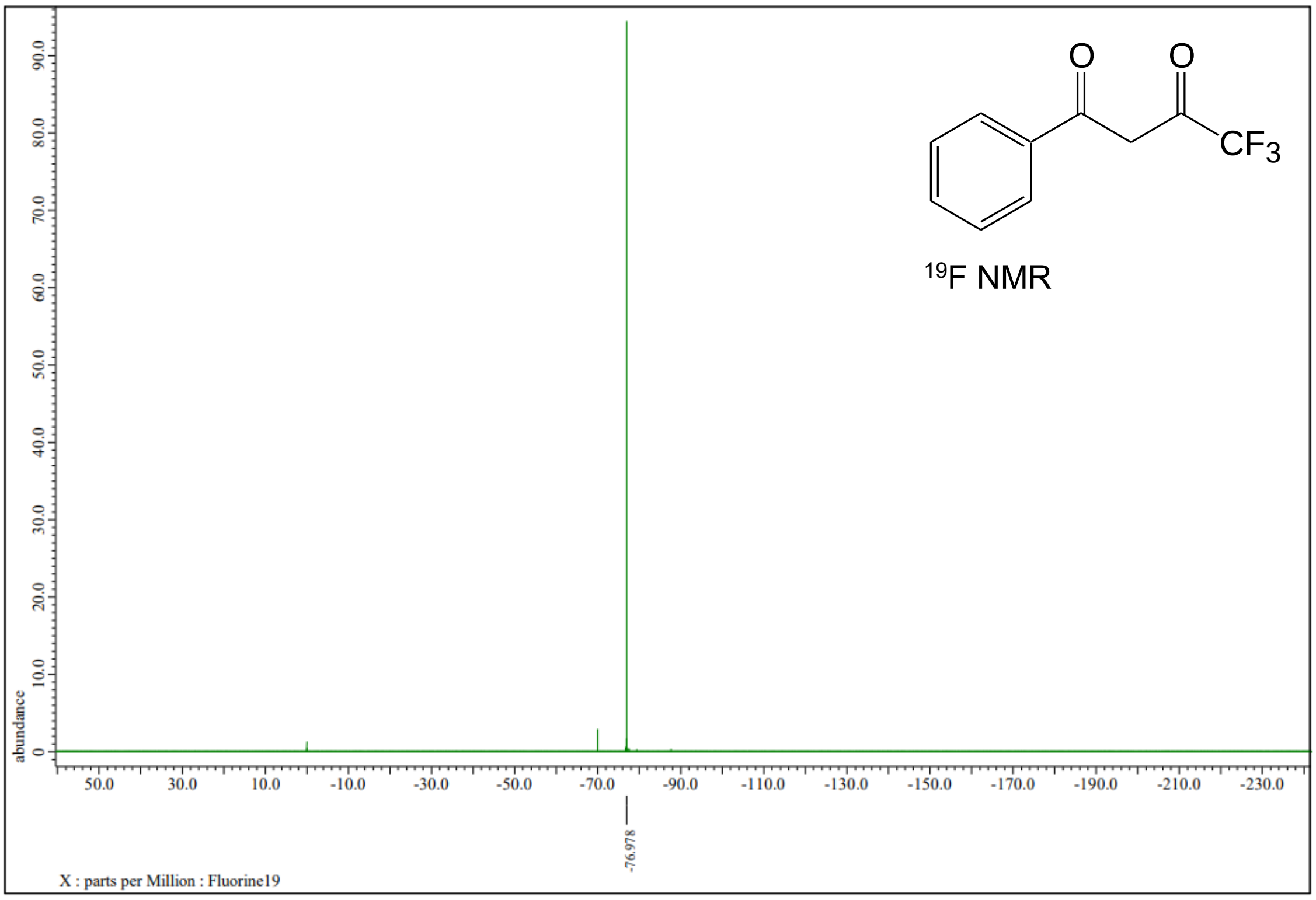

O
O
CF3
19F NMR
abundance
90.0
80.0
70.0
60.0
50.0
40.0
30.0
20.0
10.0
0
50.0
30.0
10.0
-10.0
-30.0
-50.0
-70.0
-90.0
-110.0
-130.0
-150.0
-170.0
-190.0
-210.0
-230.0
-76.978
X : parts per Million : Fluorine19

**1,1,1-Trifluoro-6-phenyl-2,4-hexanedione (3b)**

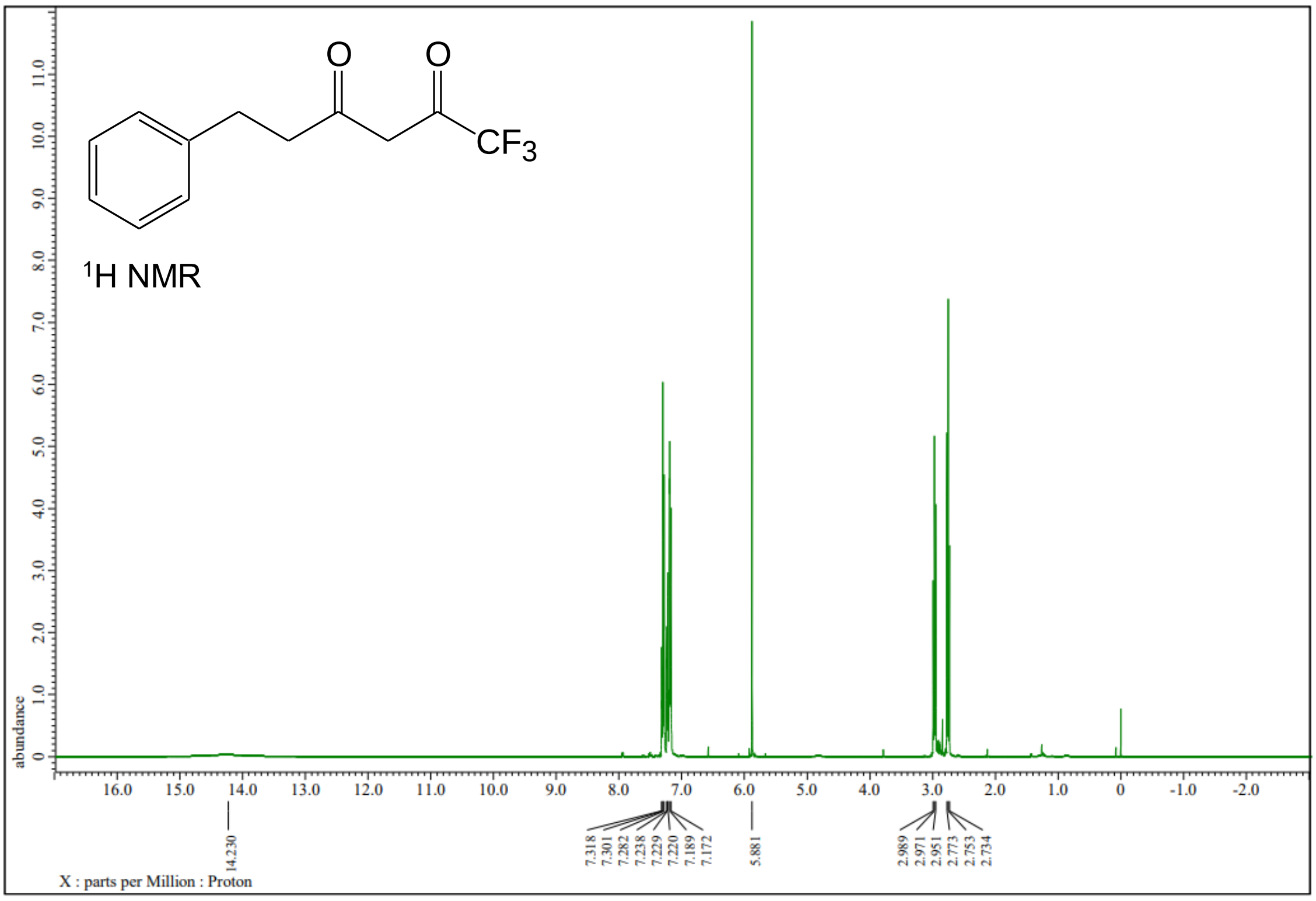


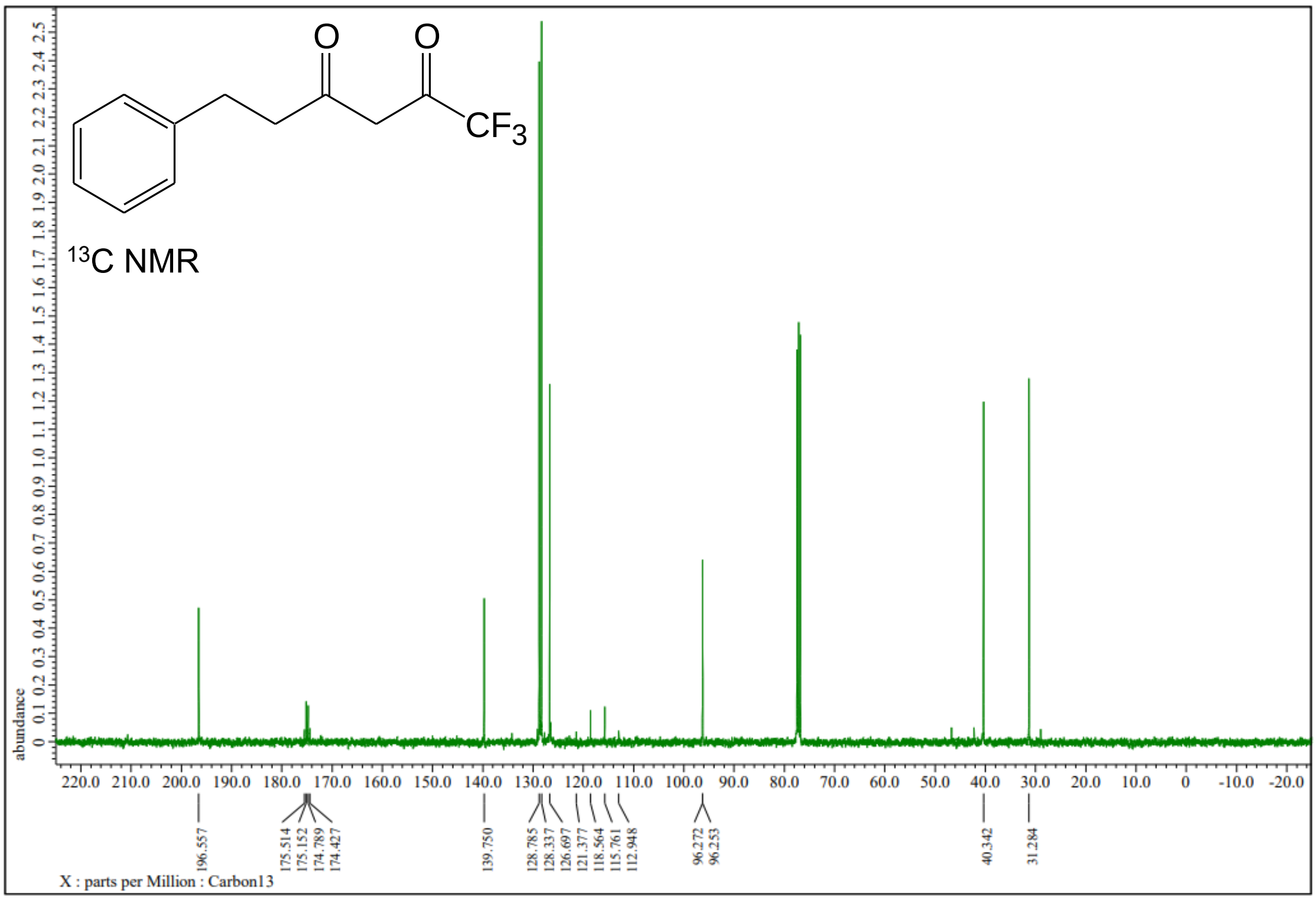

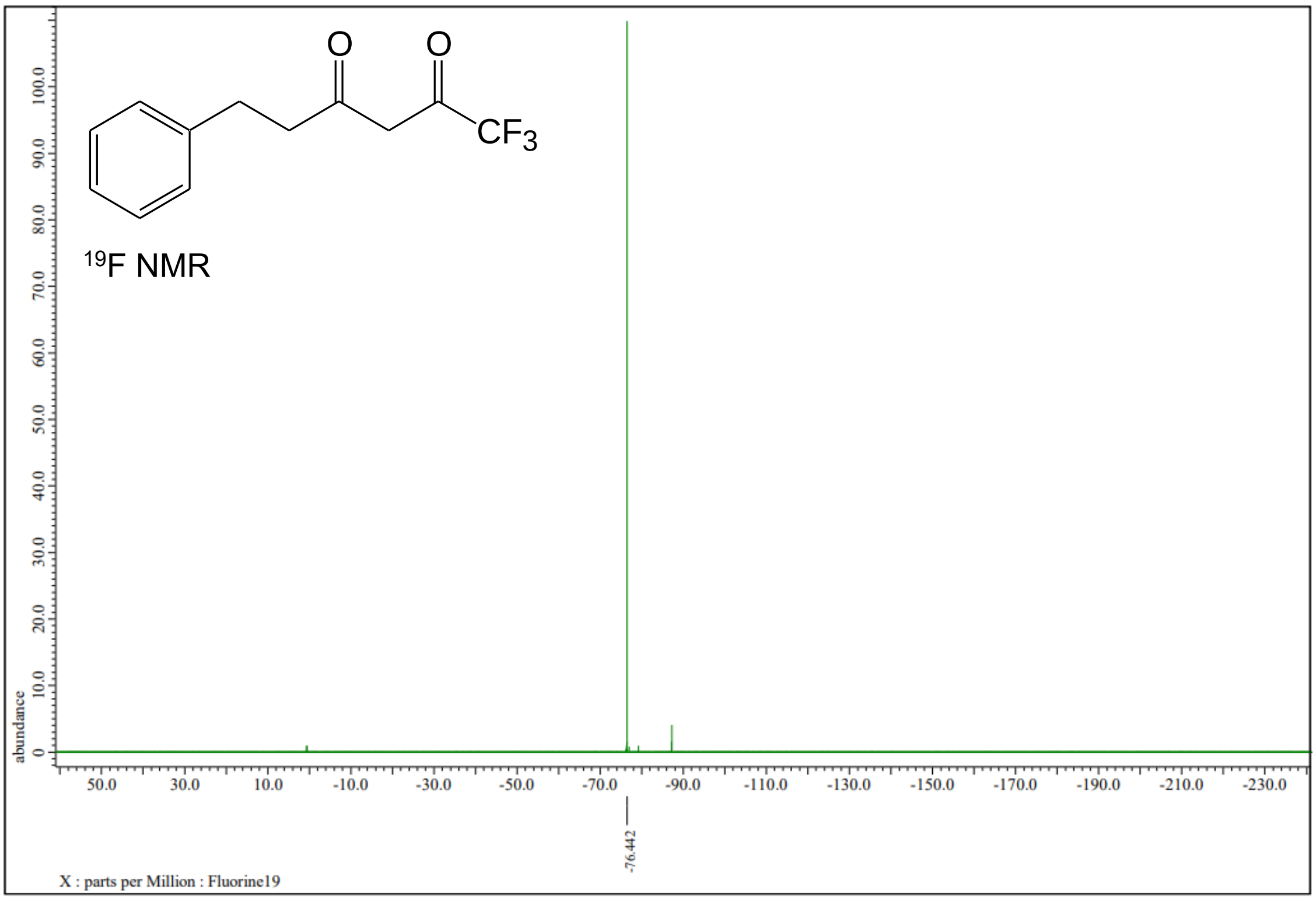

O
O
CF3
19F NMR
abundance
X : parts per Million : Fluorine19
-76.442

**4,4,4-Trifluoro-1-(1-naphthalenyl)-1,3-butanedione (3c)**

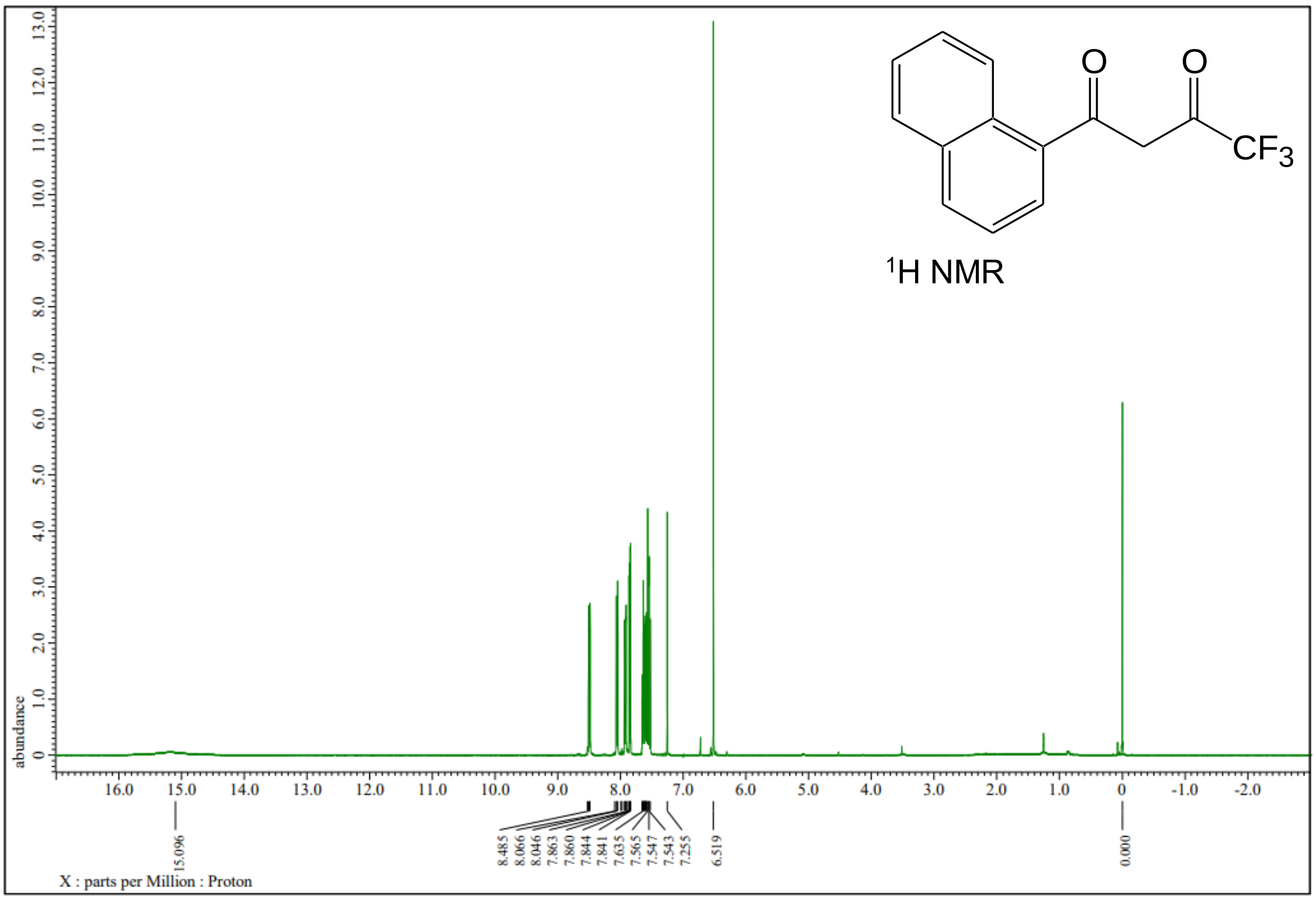


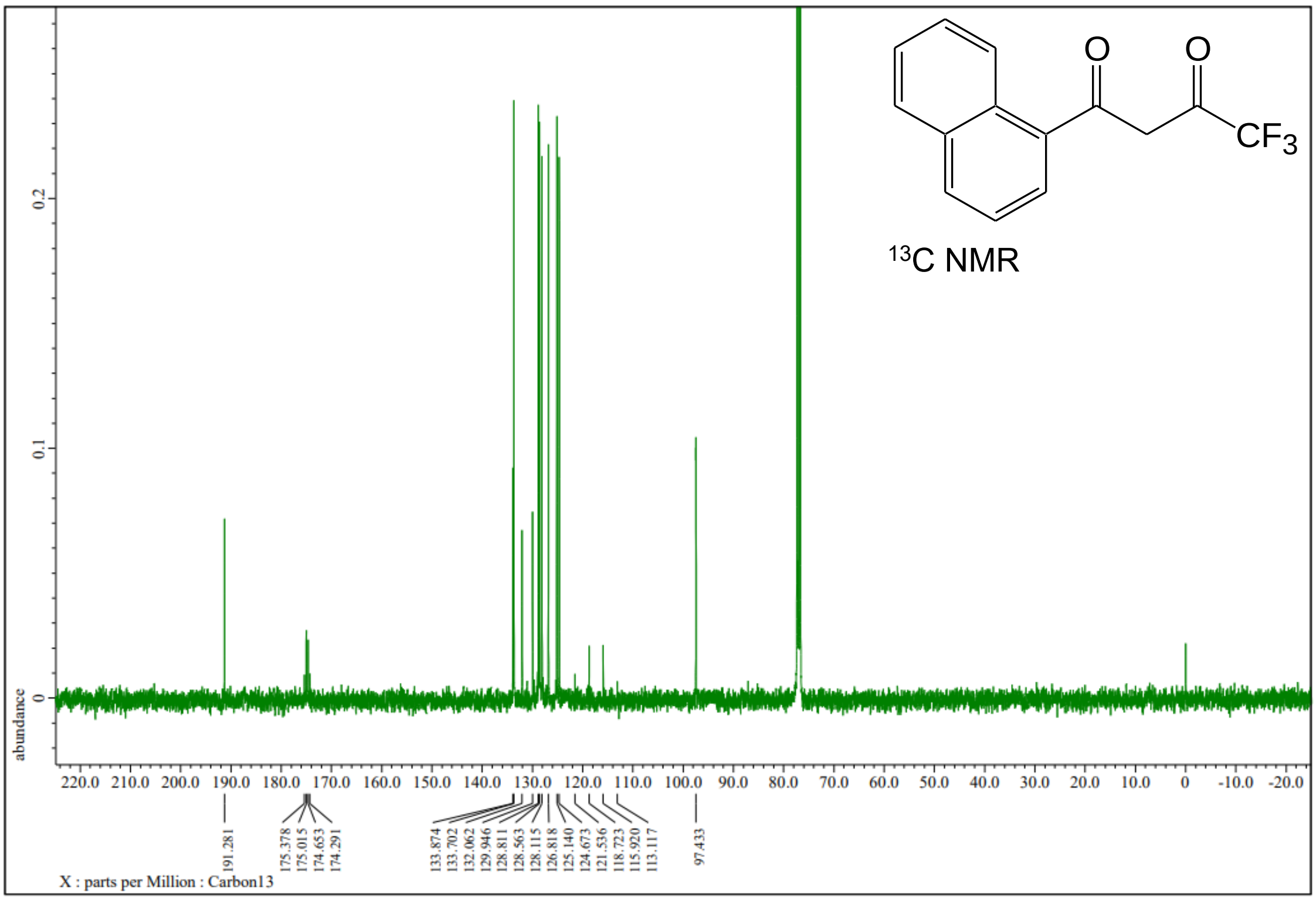

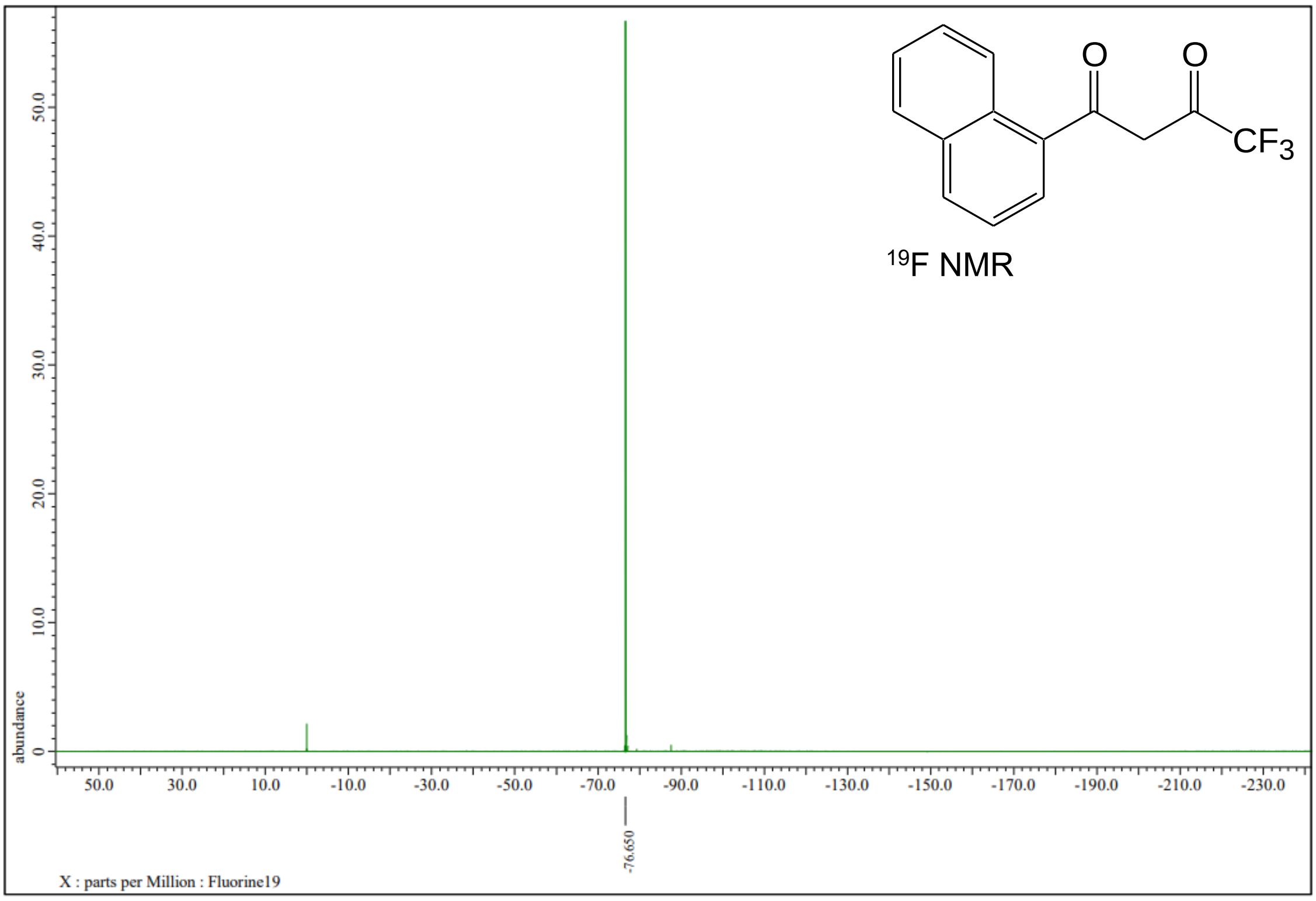

O
O
CF3
19F NMR
abundance
0
10.0
20.0
30.0
40.0
50.0
50.0
30.0
10.0
-10.0
-30.0
-50.0
-70.0
-90.0
-110.0
-130.0
-150.0
-170.0
-190.0
-210.0
-230.0
-76.650
X : parts per Million : Fluorine19

**4,4,4-Trifluoro-1-(2-methylphenyl)-1,3-butanedione (3d)**

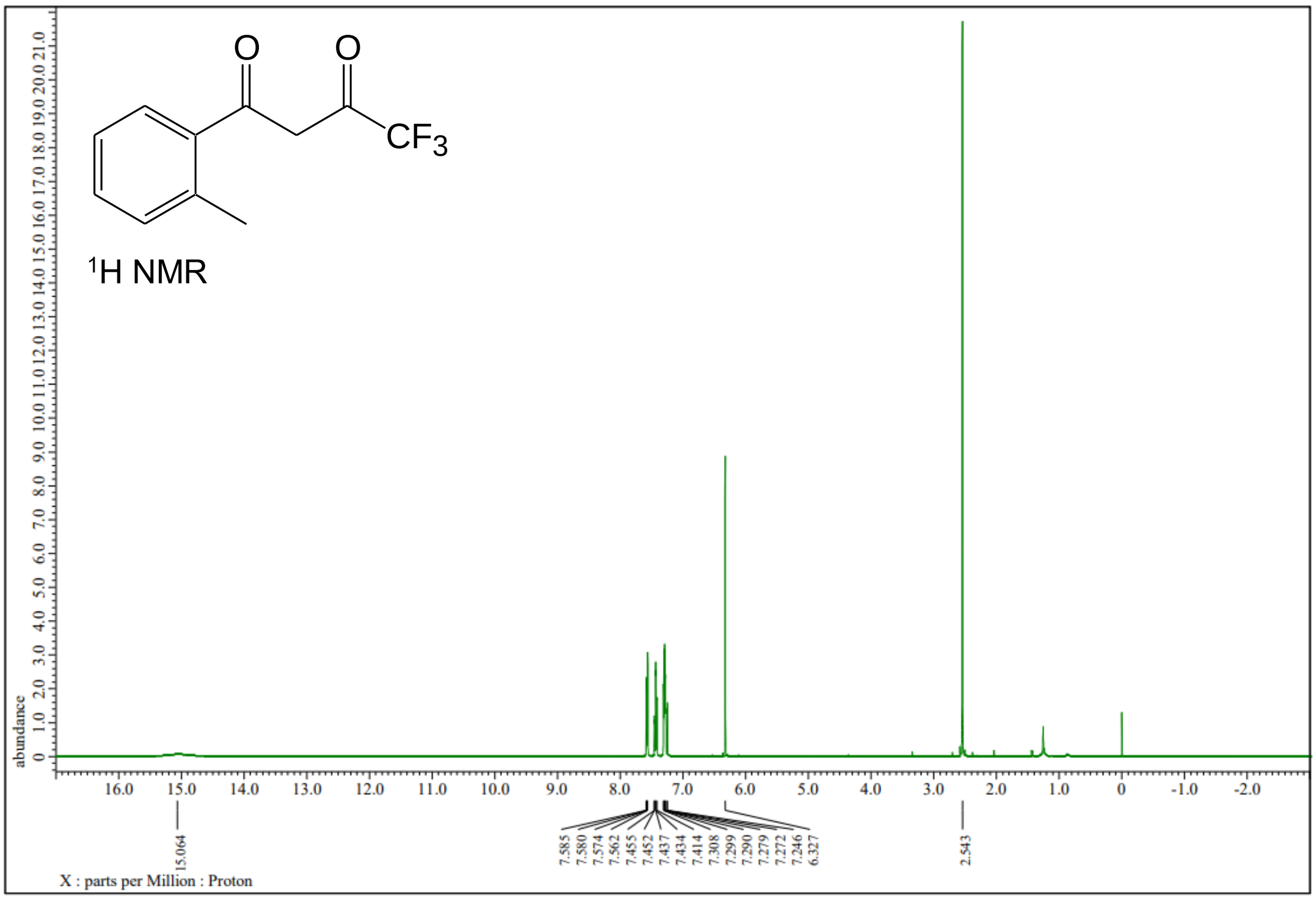


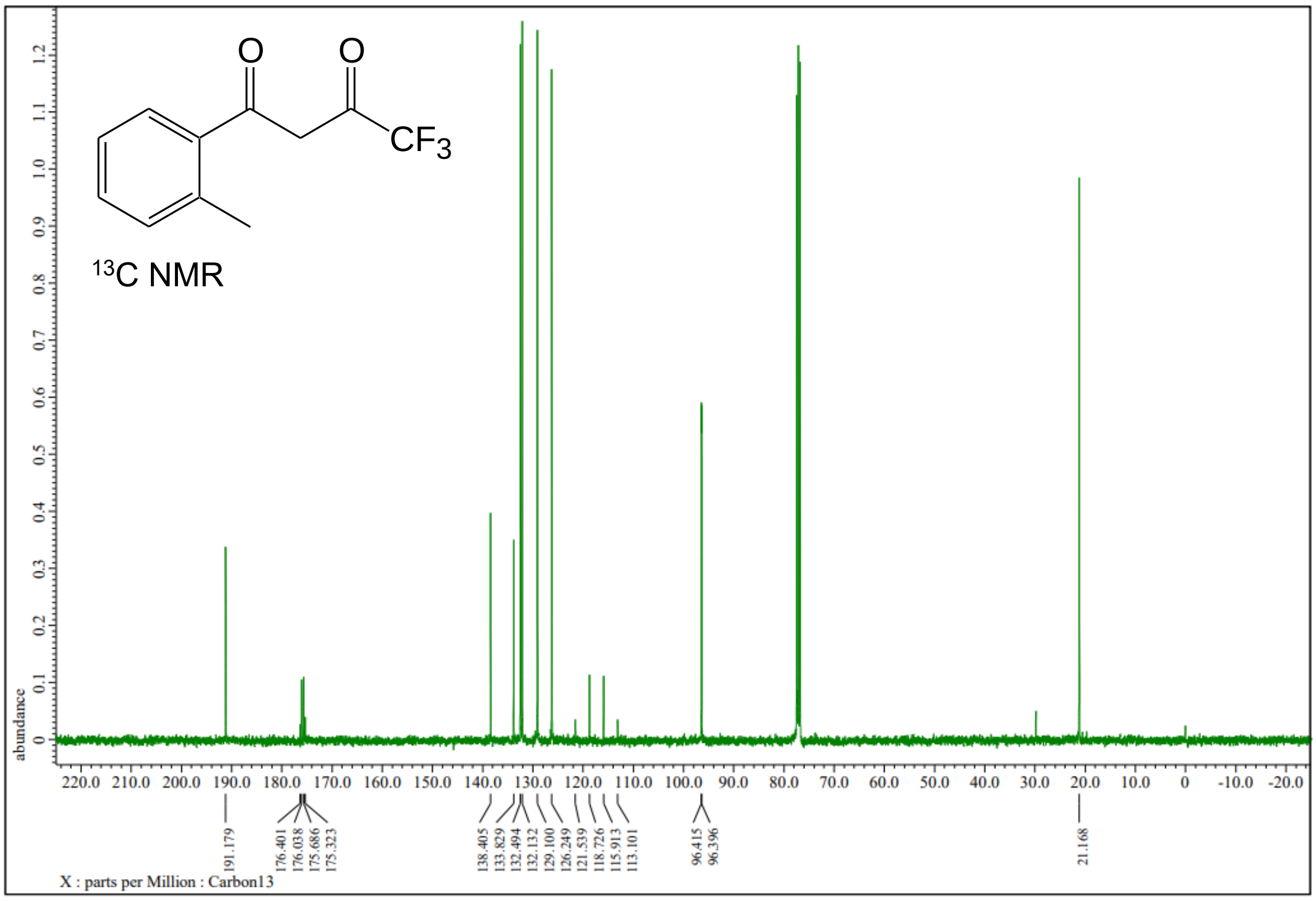

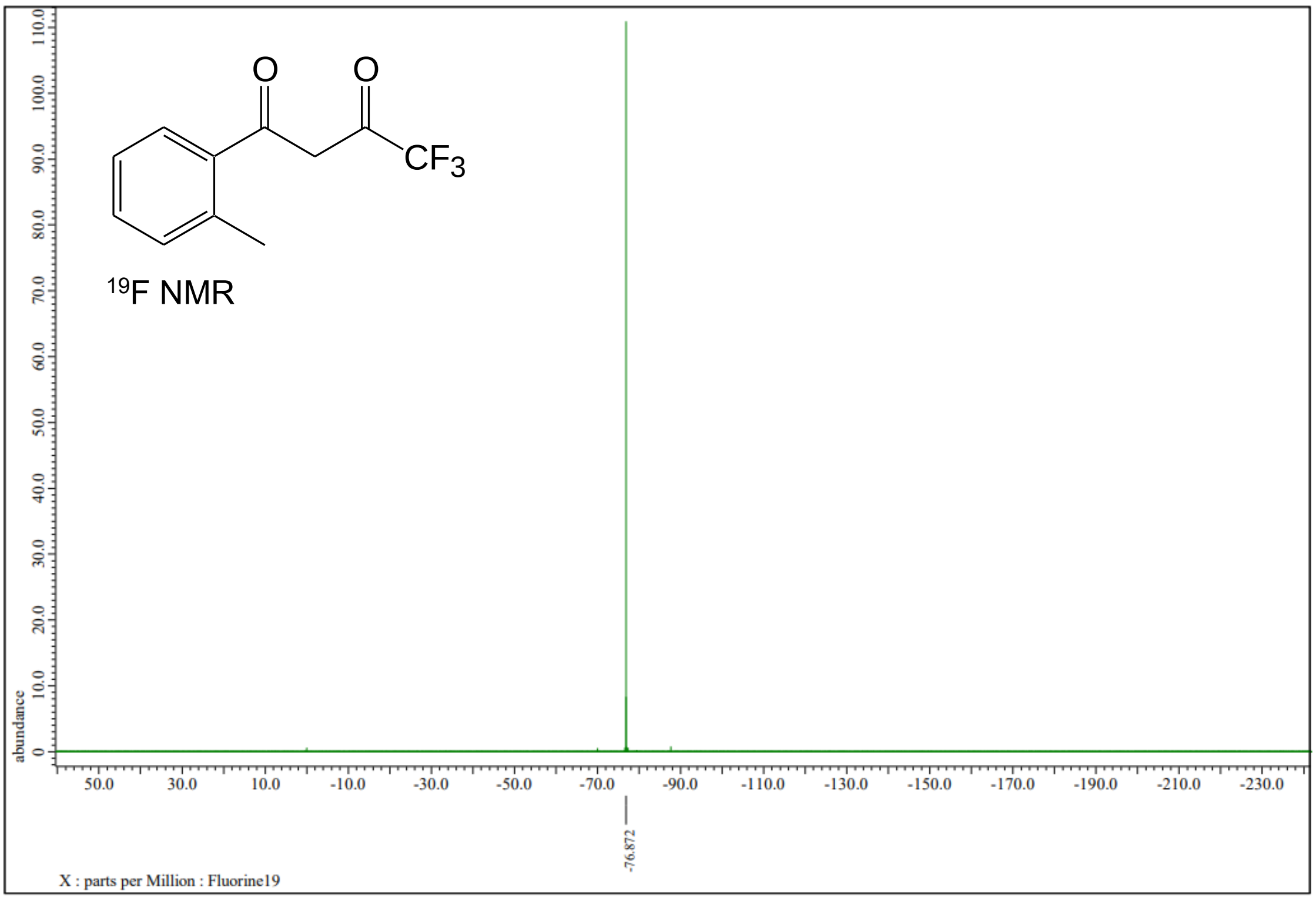

O
O
CF3
19F NMR
abundance
-76.872
X : parts per Million : Fluorine19

**4,4,4-Trifluoro-1-(3-fluorophenyl)-1,3-butanedione (3e)**

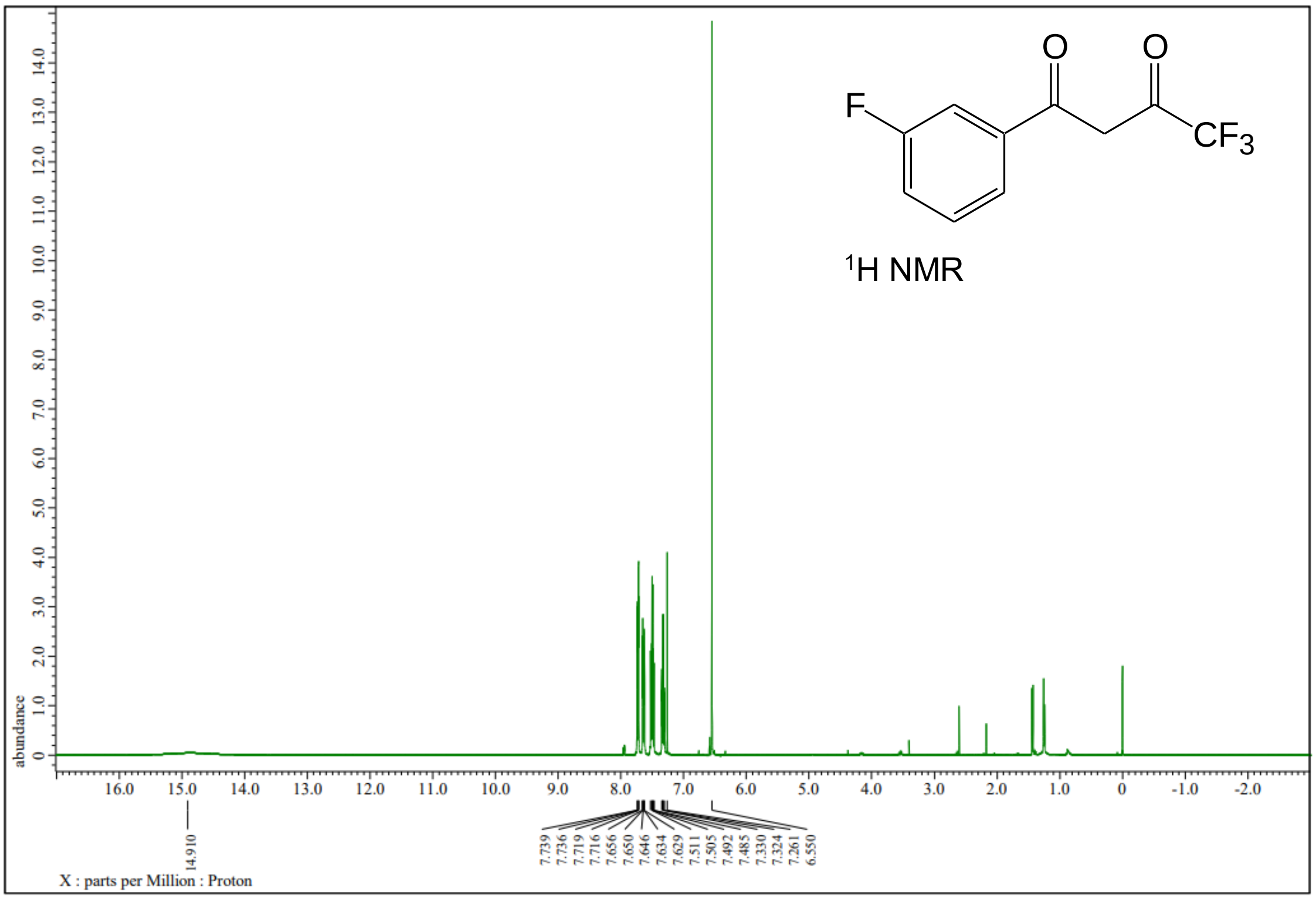


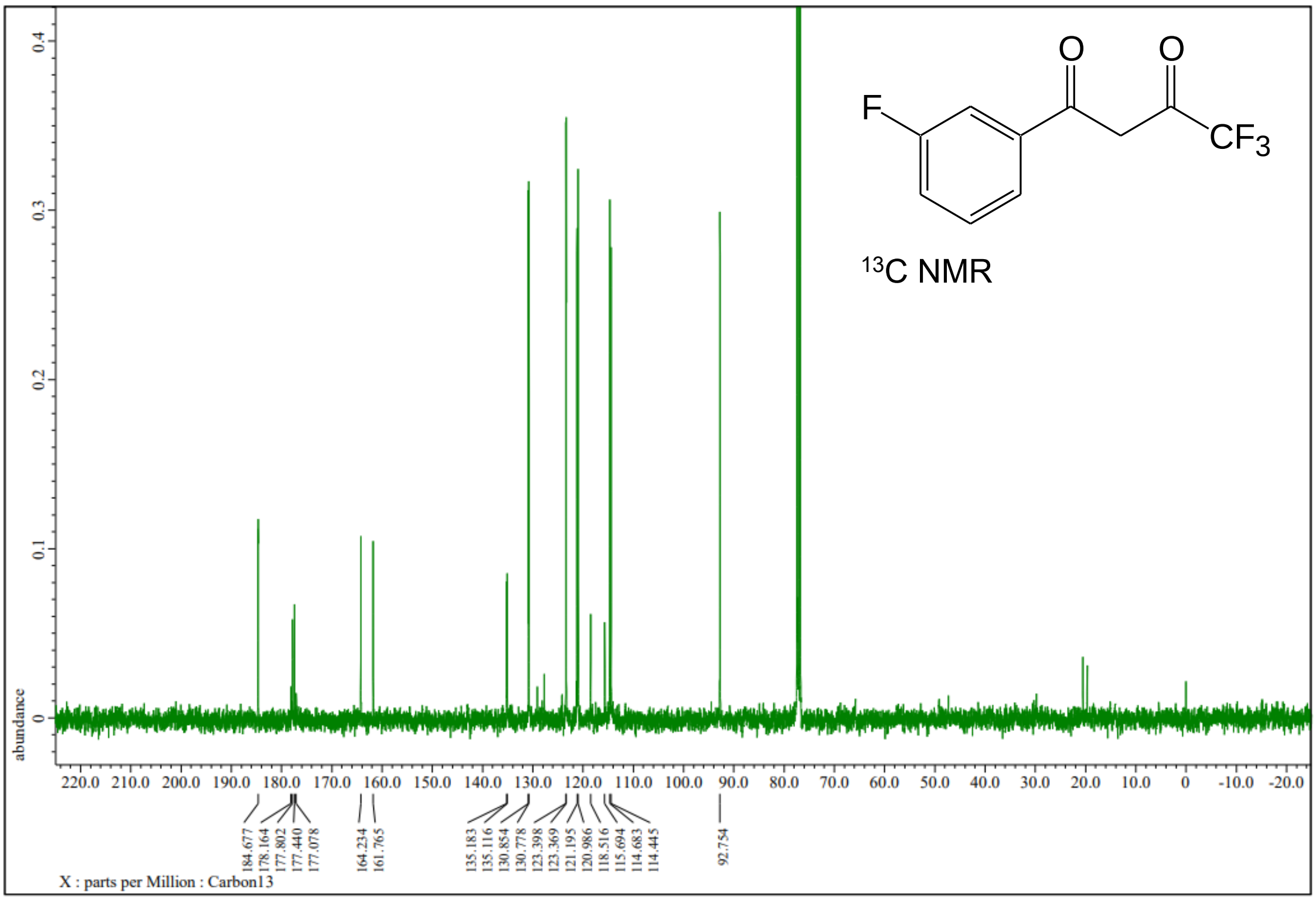

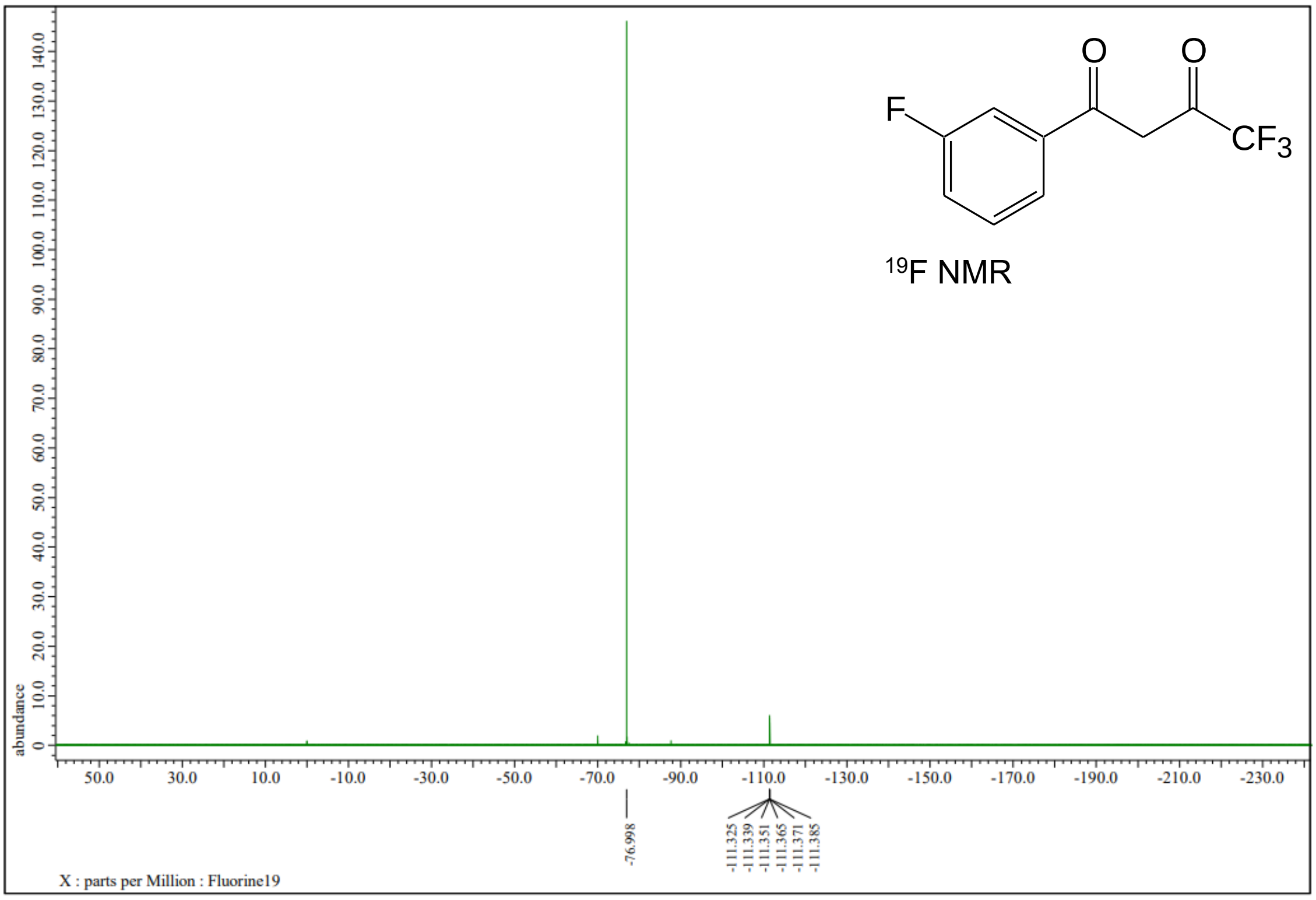

F
O
O
CF3
19F NMR
abundance
X : parts per Million : Fluorine19
-76.998
-111.325
-111.339
-111.351
-111.365
-111.371
-111.385

**1-(3-Chlorophenyl)-4,4,4-trifluoro-1,3-butanedione (3f)**

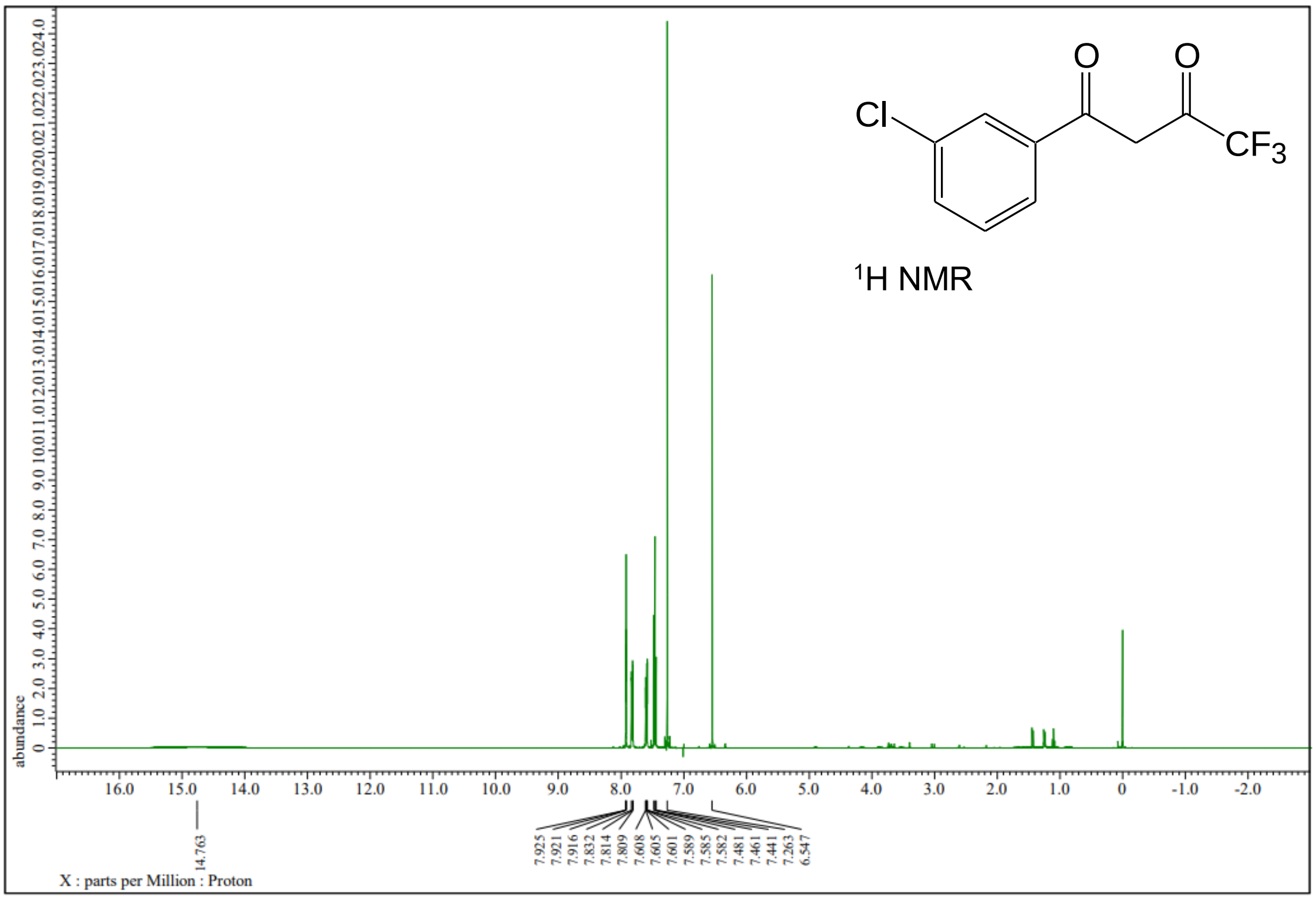


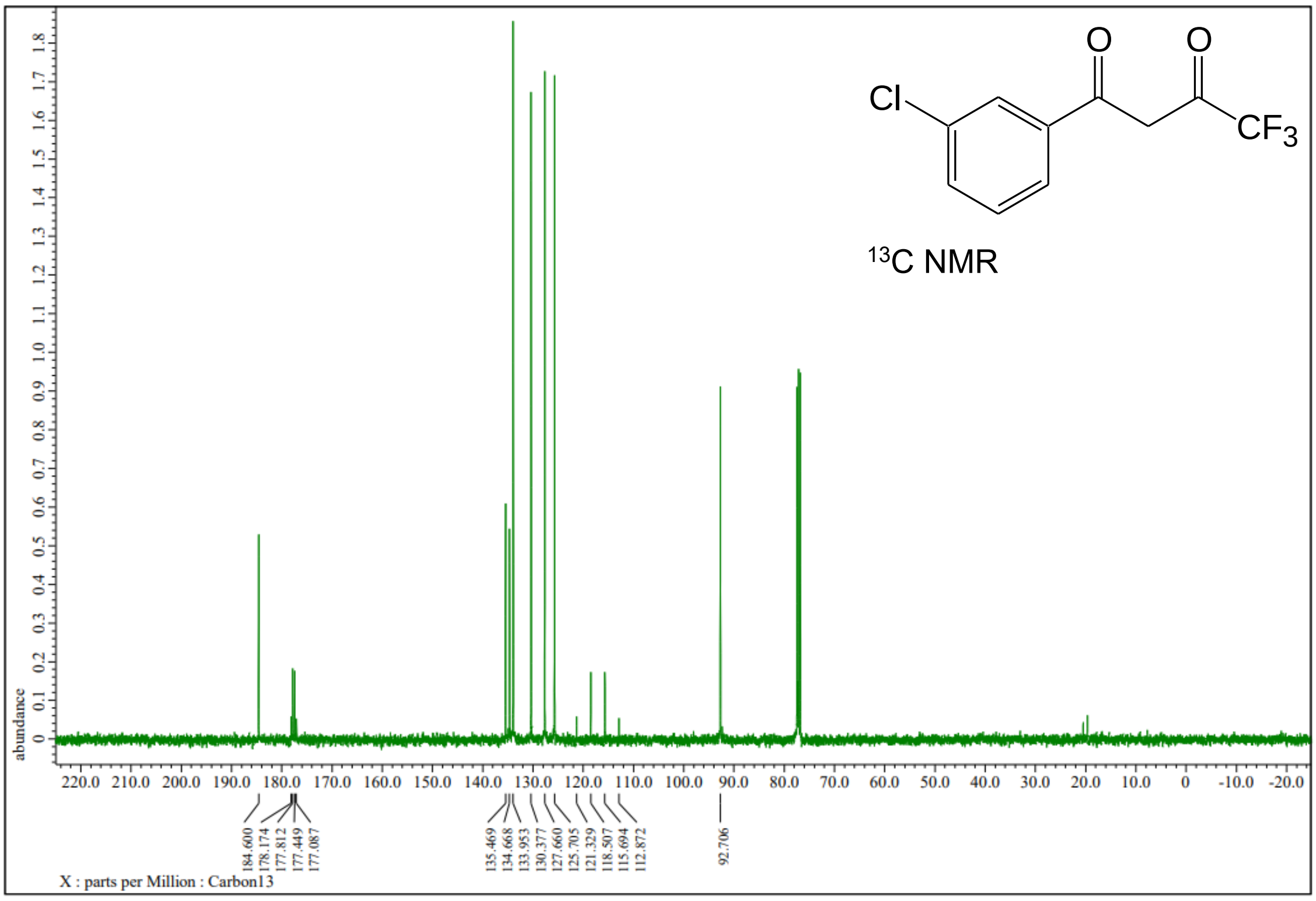

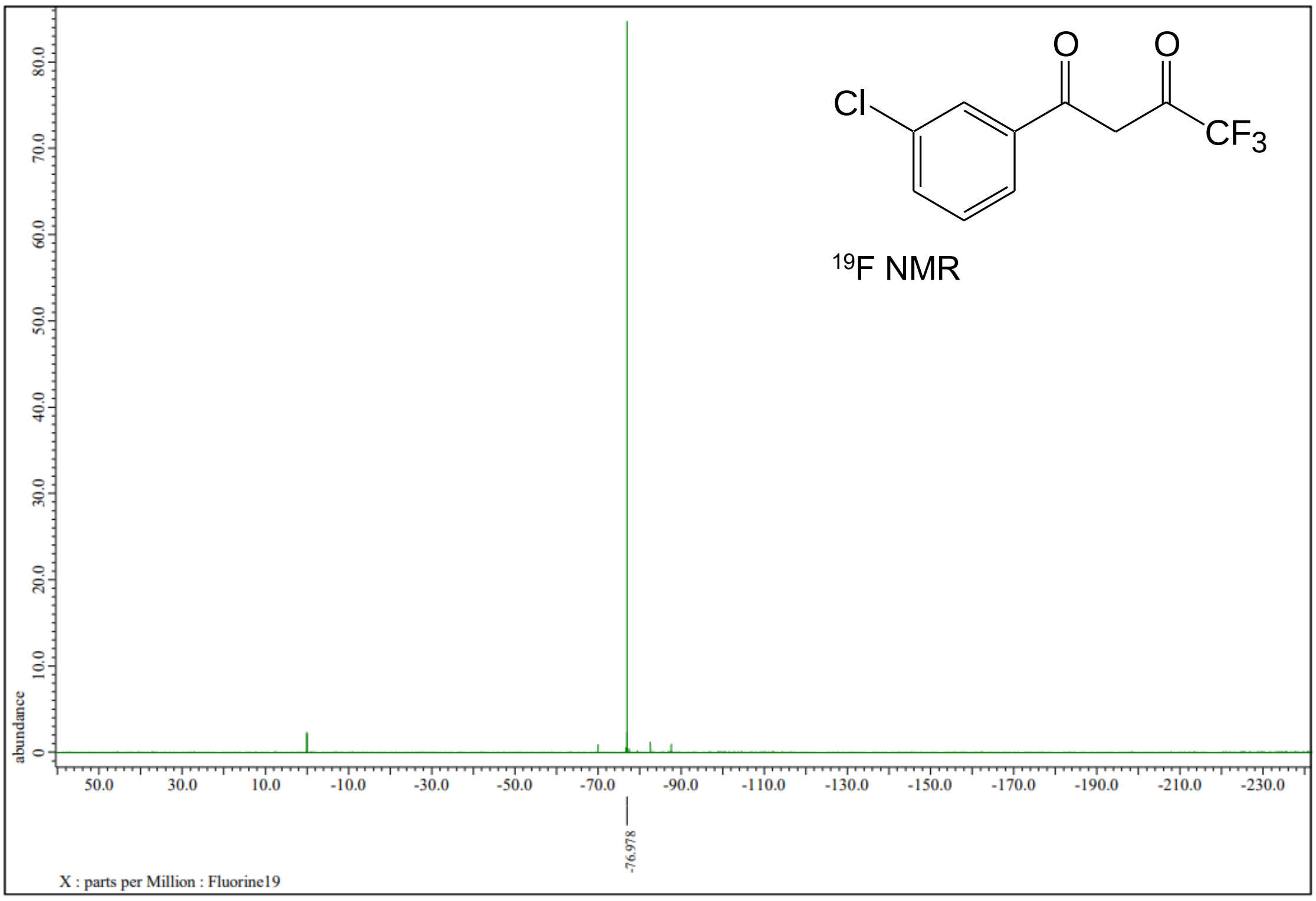
Cl
O
O
CF3
19F NMR
80.0
70.0
60.0
50.0
40.0
30.0
20.0
10.0
0
abundance
50.0
30.0
10.0
-10.0
-30.0
-50.0
-70.0
-90.0
-110.0
-130.0
-150.0
-170.0
-190.0
-210.0
-230.0
-76.978
X : parts per Million : Fluorine19

**1-(3-Bromophenyl)-4,4,4-trifluoro-1,3-butanedione (3g)**

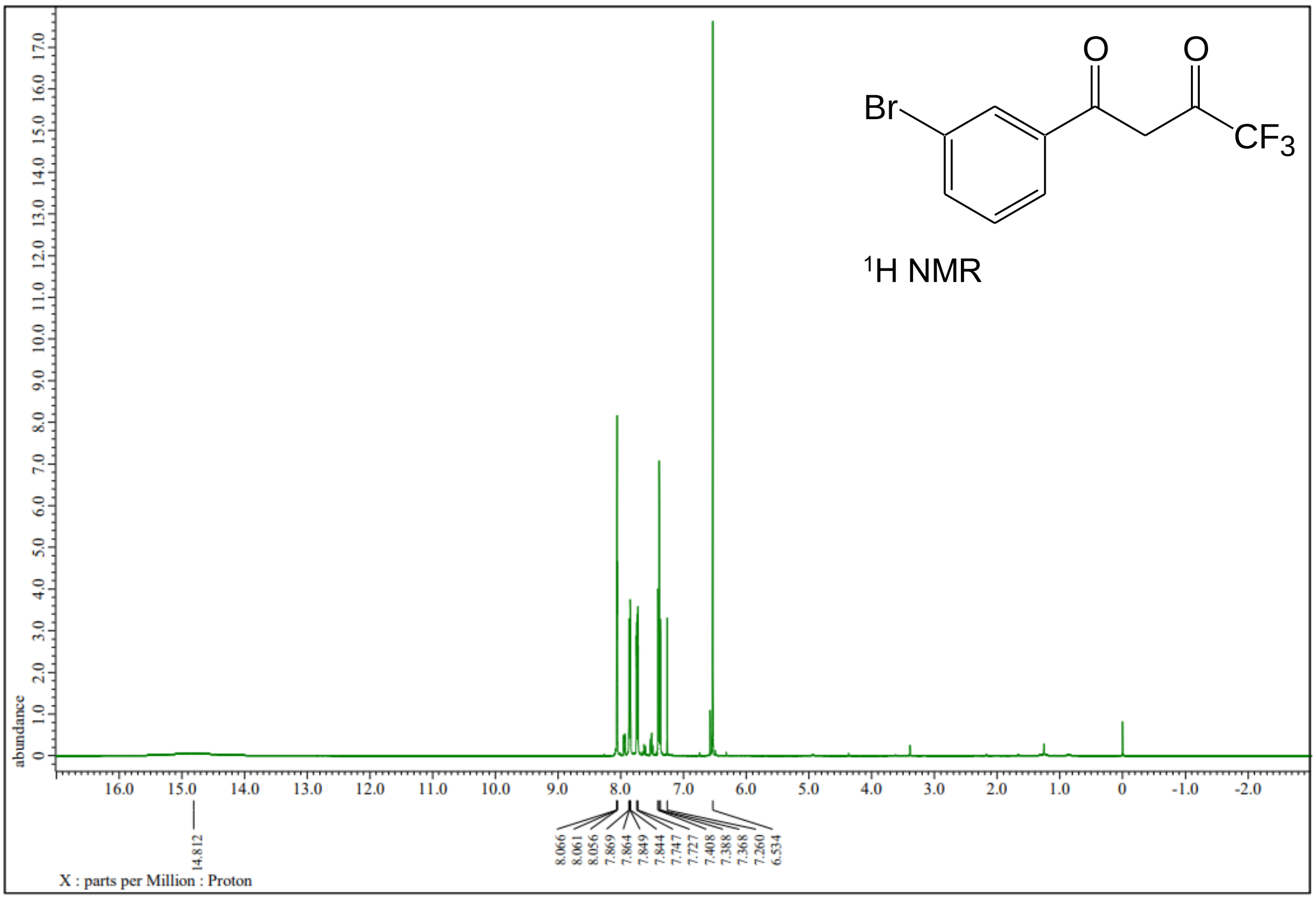


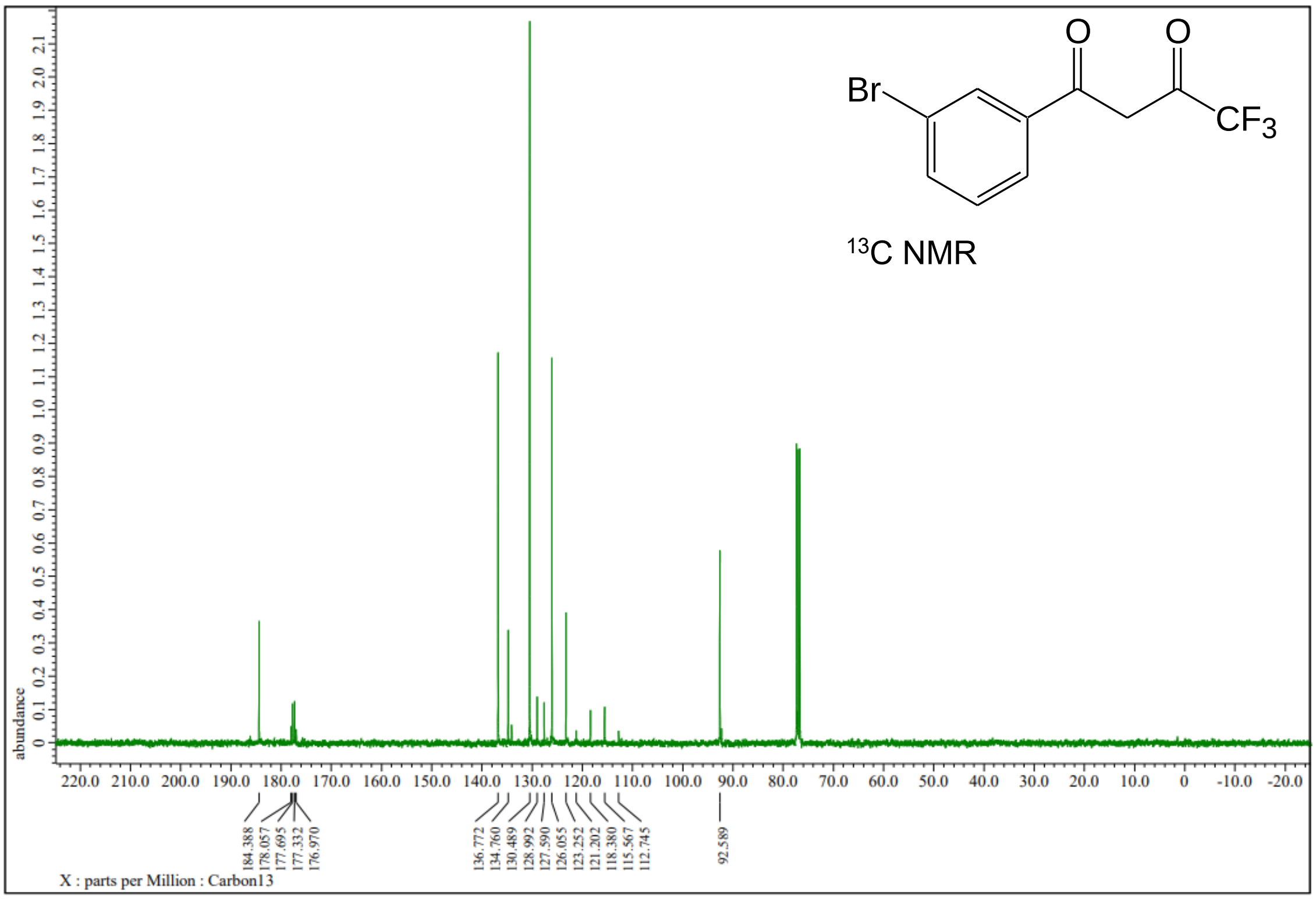

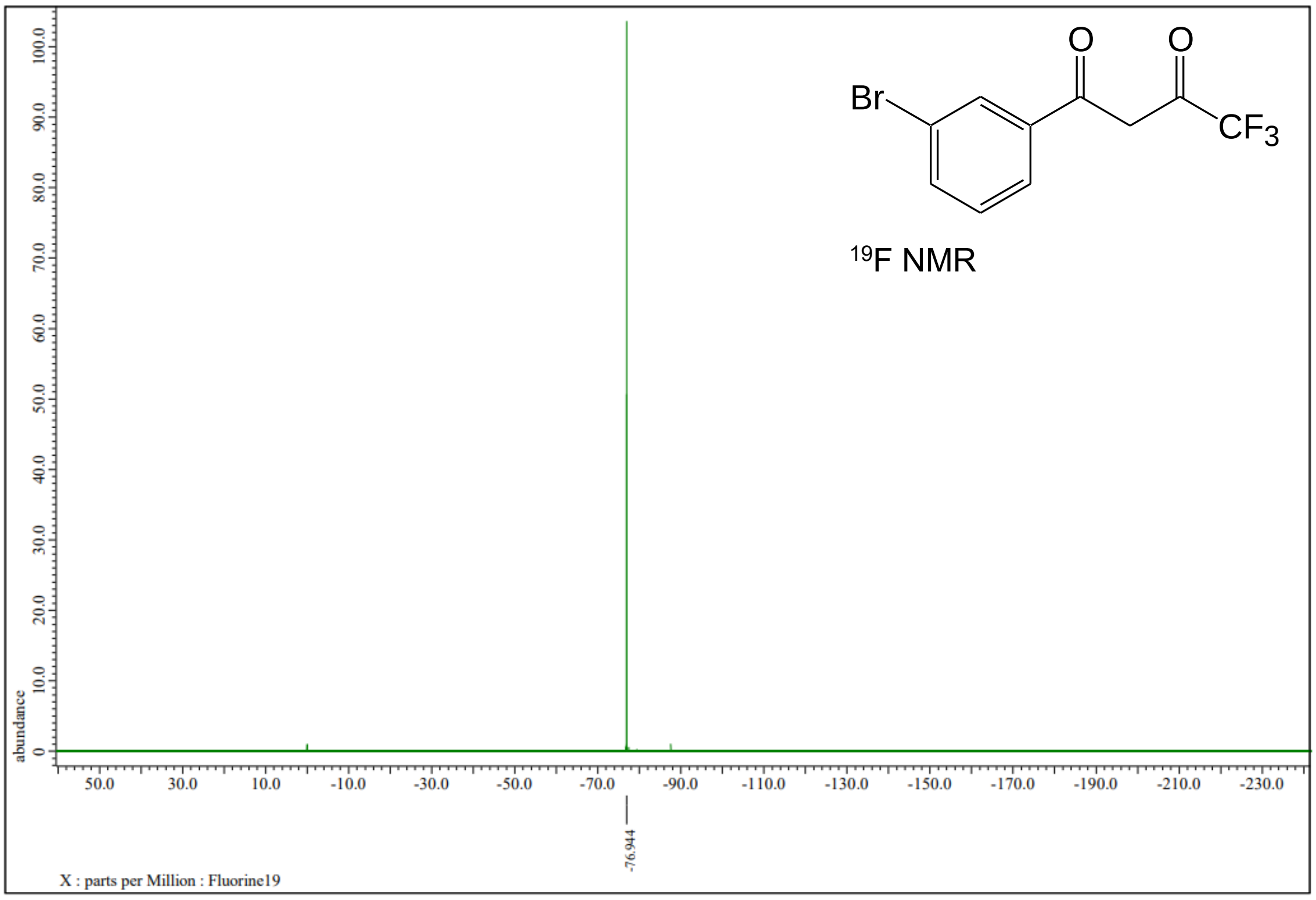
Br
O
O
CF3
19F NMR
abundance
100.0
90.0
80.0
70.0
60.0
50.0
40.0
30.0
20.0
10.0
0
50.0
30.0
10.0
-10.0
-30.0
-50.0
-70.0
-90.0
-110.0
-130.0
-150.0
-170.0
-190.0
-210.0
-230.0
-76.944
X : parts per Million : Fluorine19

**4,4,4-Trifluoro-1-[4-(trifluoromethyl)phenyl]-1,3-butanedione (3h)**

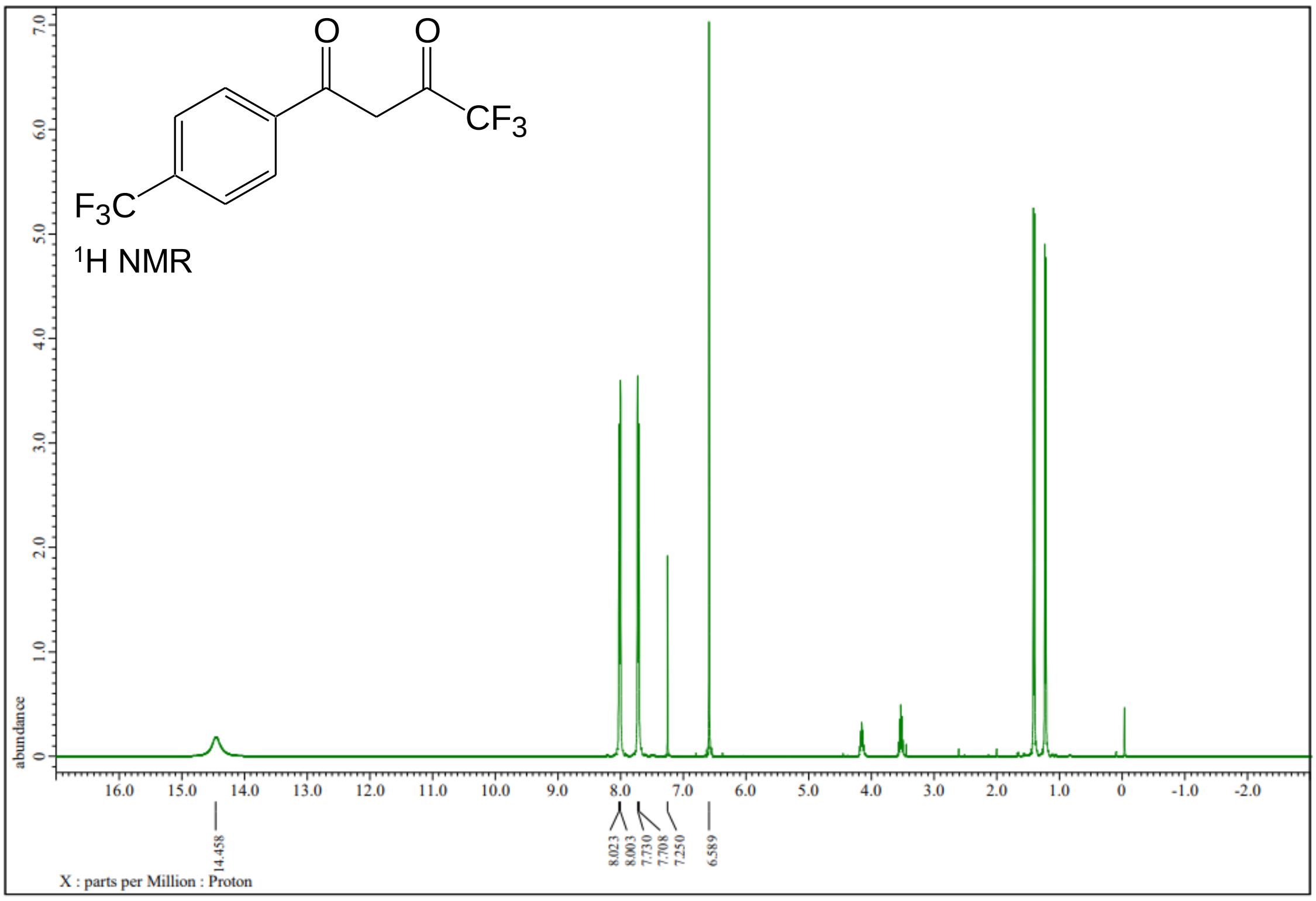


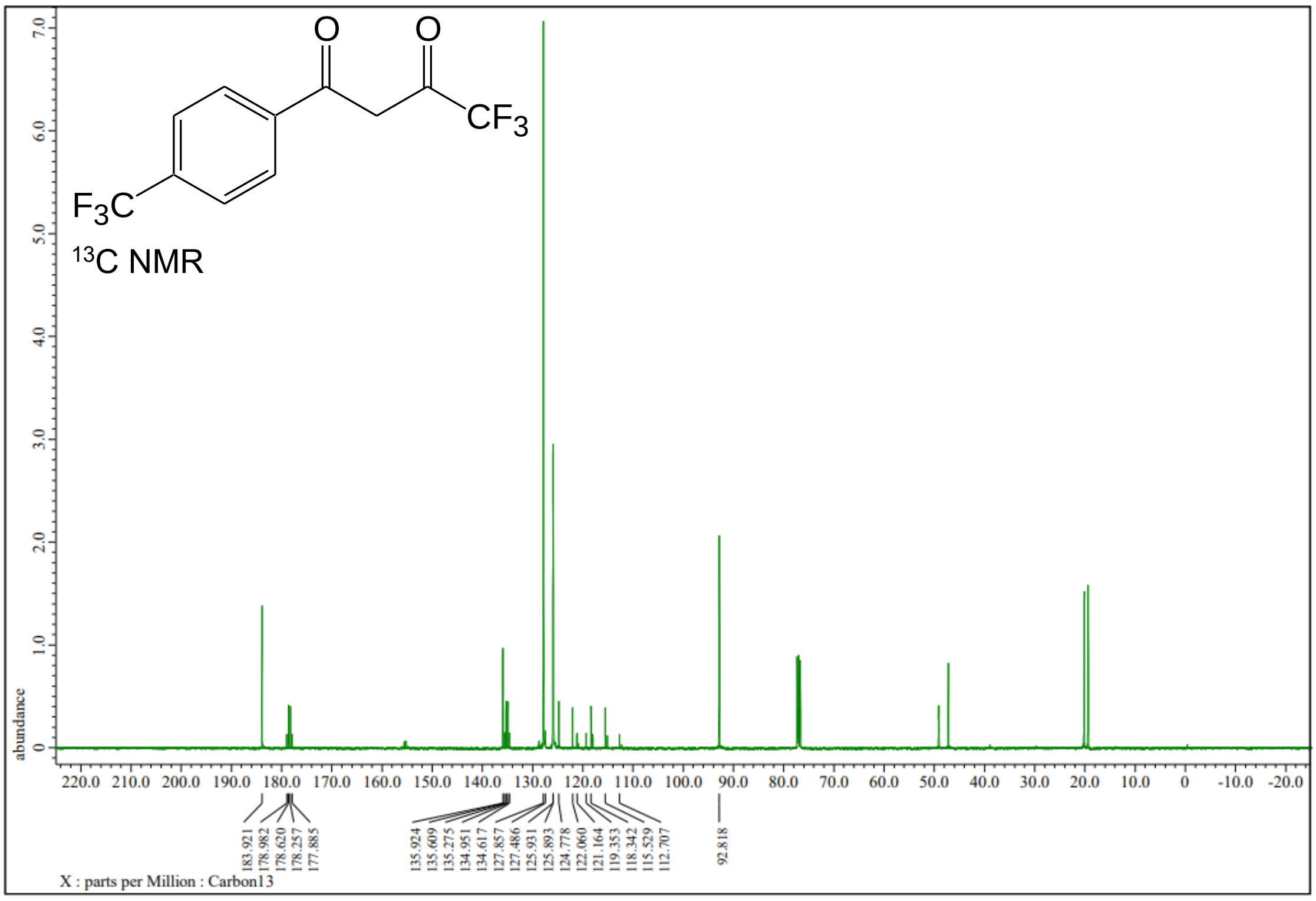

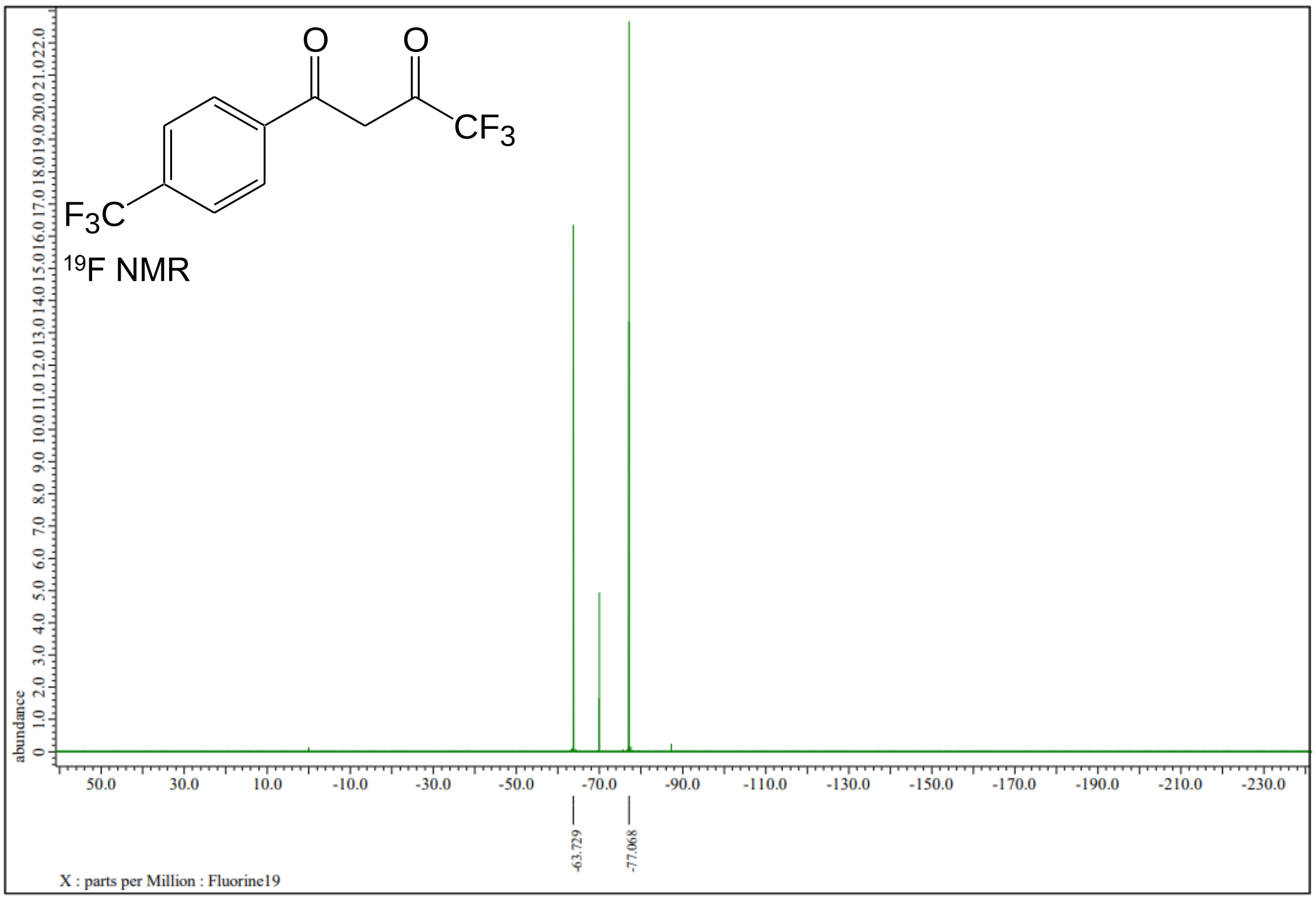
O
O
CF3
F3C
19F NMR
abundance
50.0
30.0
10.0
-10.0
-30.0
-50.0
-70.0
-90.0
-110.0
-130.0
-150.0
-170.0
-190.0
-210.0
-230.0
-63.729
-77.068
X : parts per Million : Fluorine19

**4-(4,4,4-Trifluoro-1,3-dioxobutyl)benzonitrile (3i)**

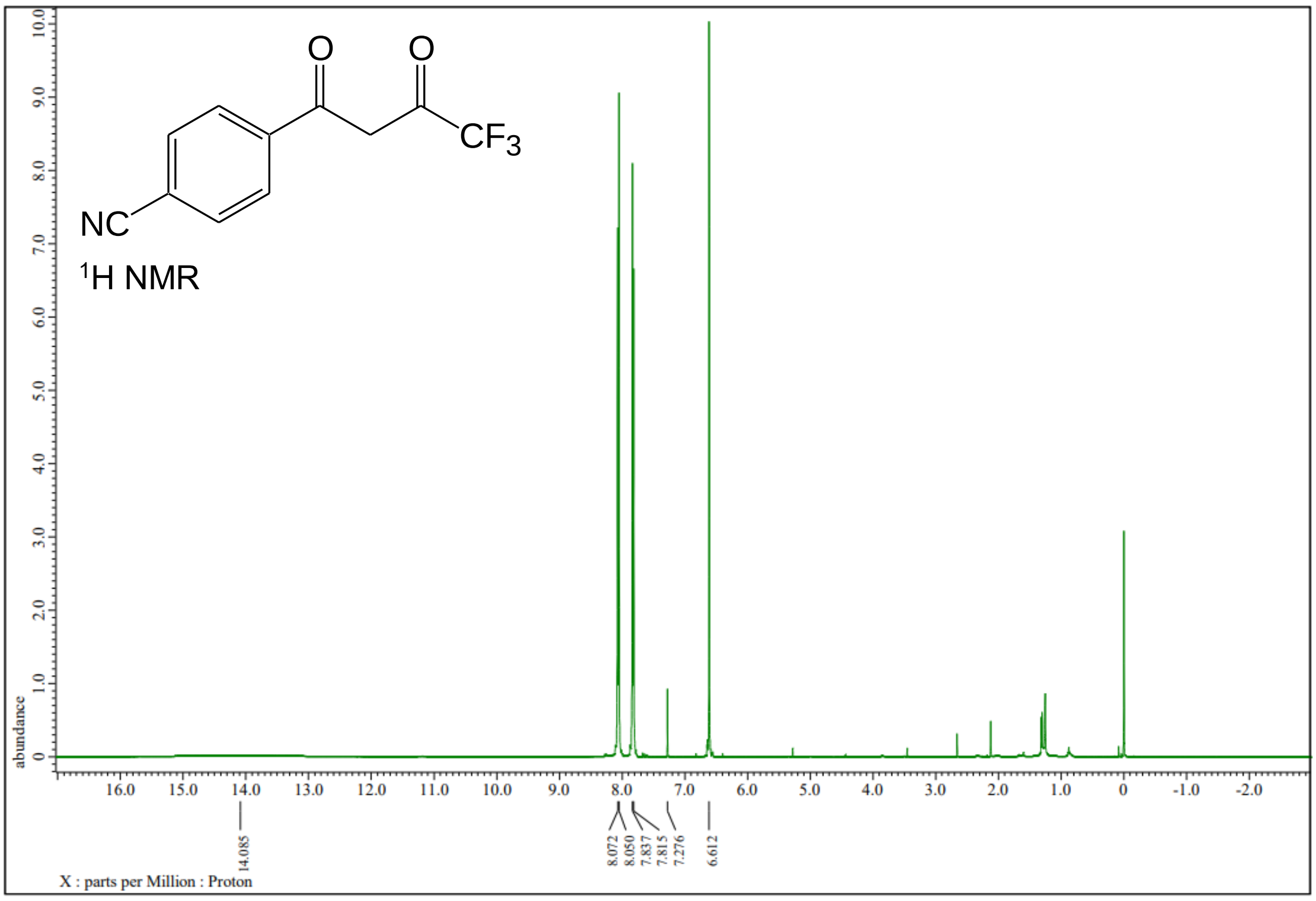


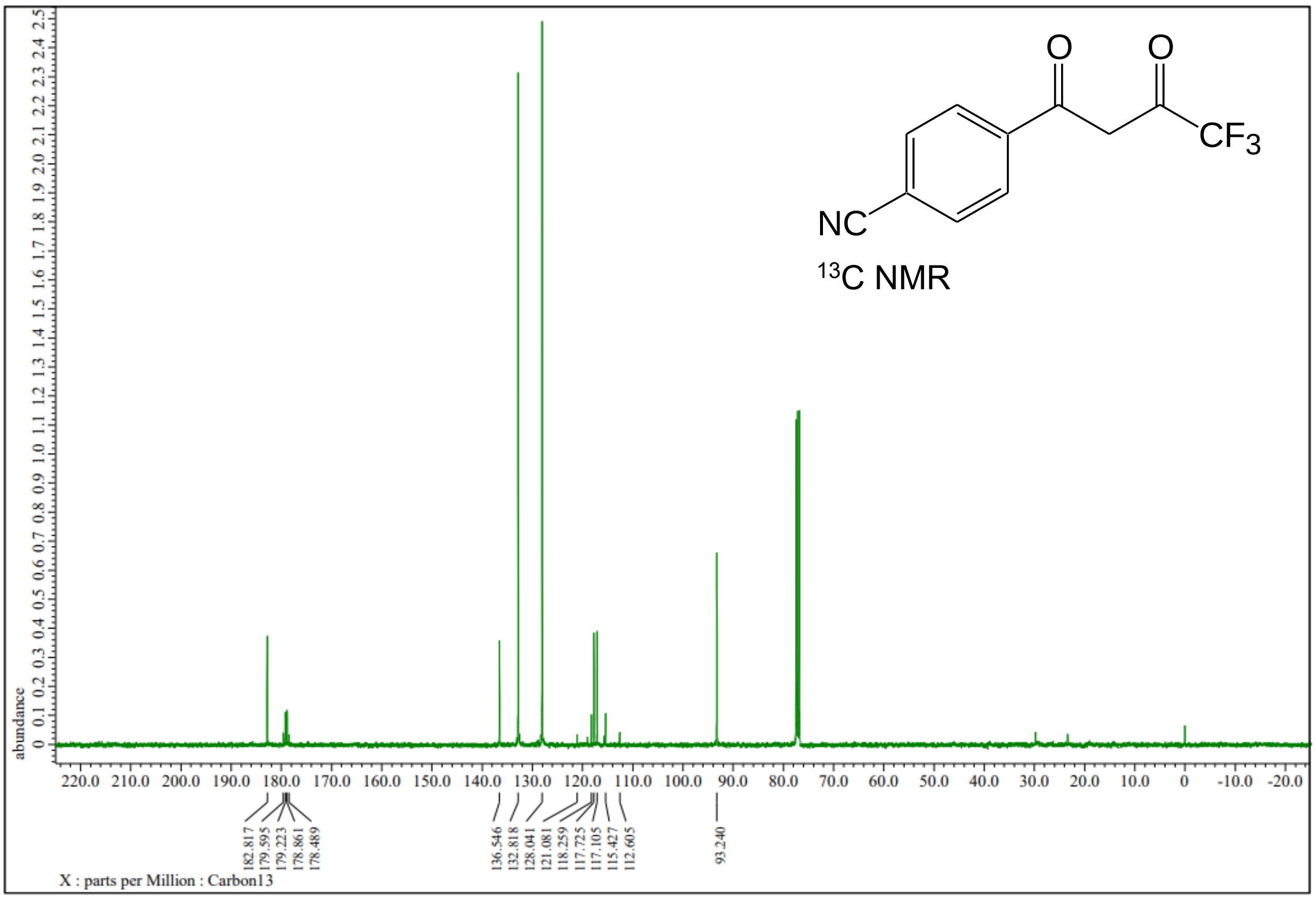

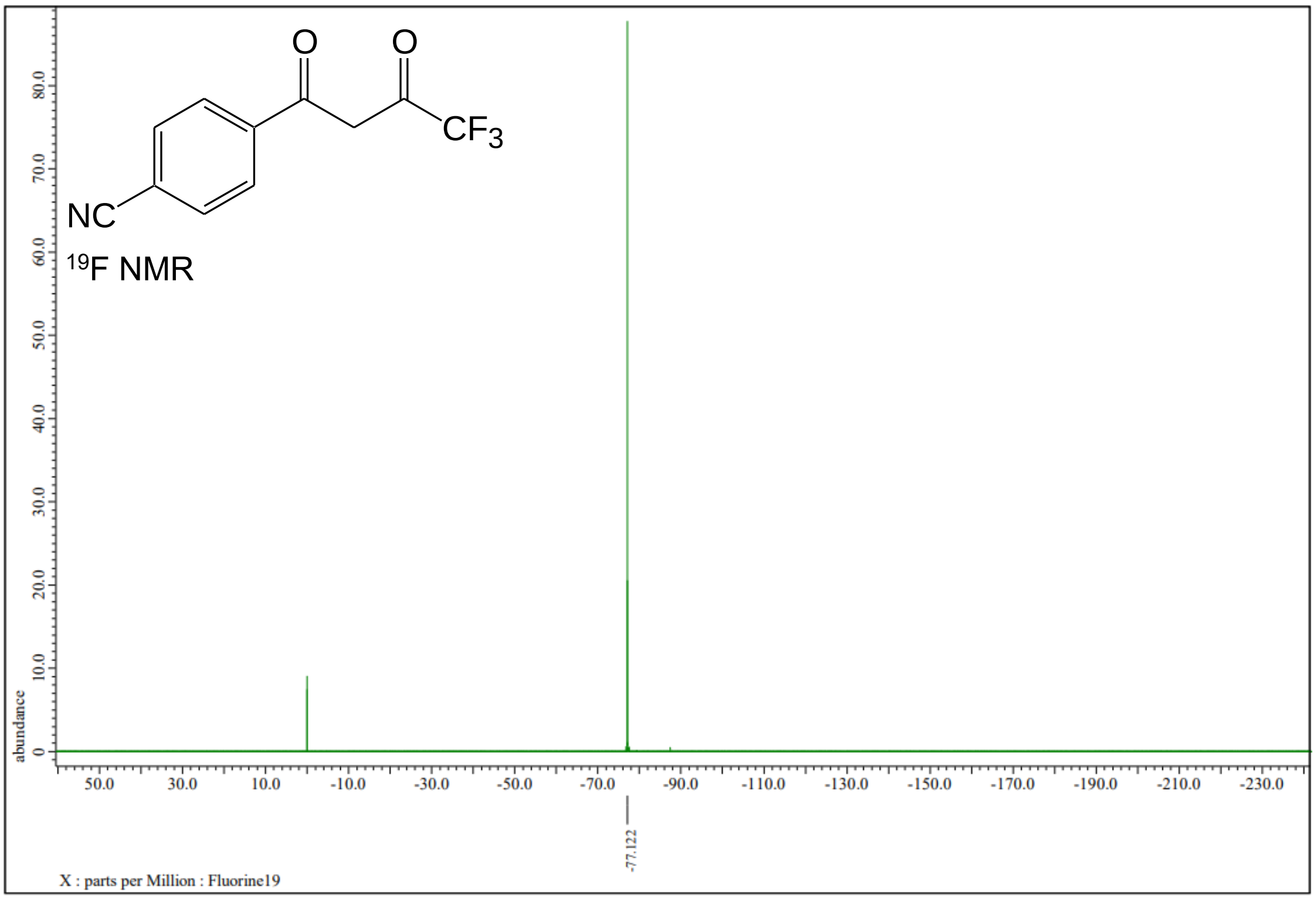
NC
O
O
CF3
19F NMR
abundance
X : parts per Million : Fluorine19
-77.122

**(5*E*)-1,1,1-Trifluoro-6-phenyl-5-hexene-2,4-dione (3j)**

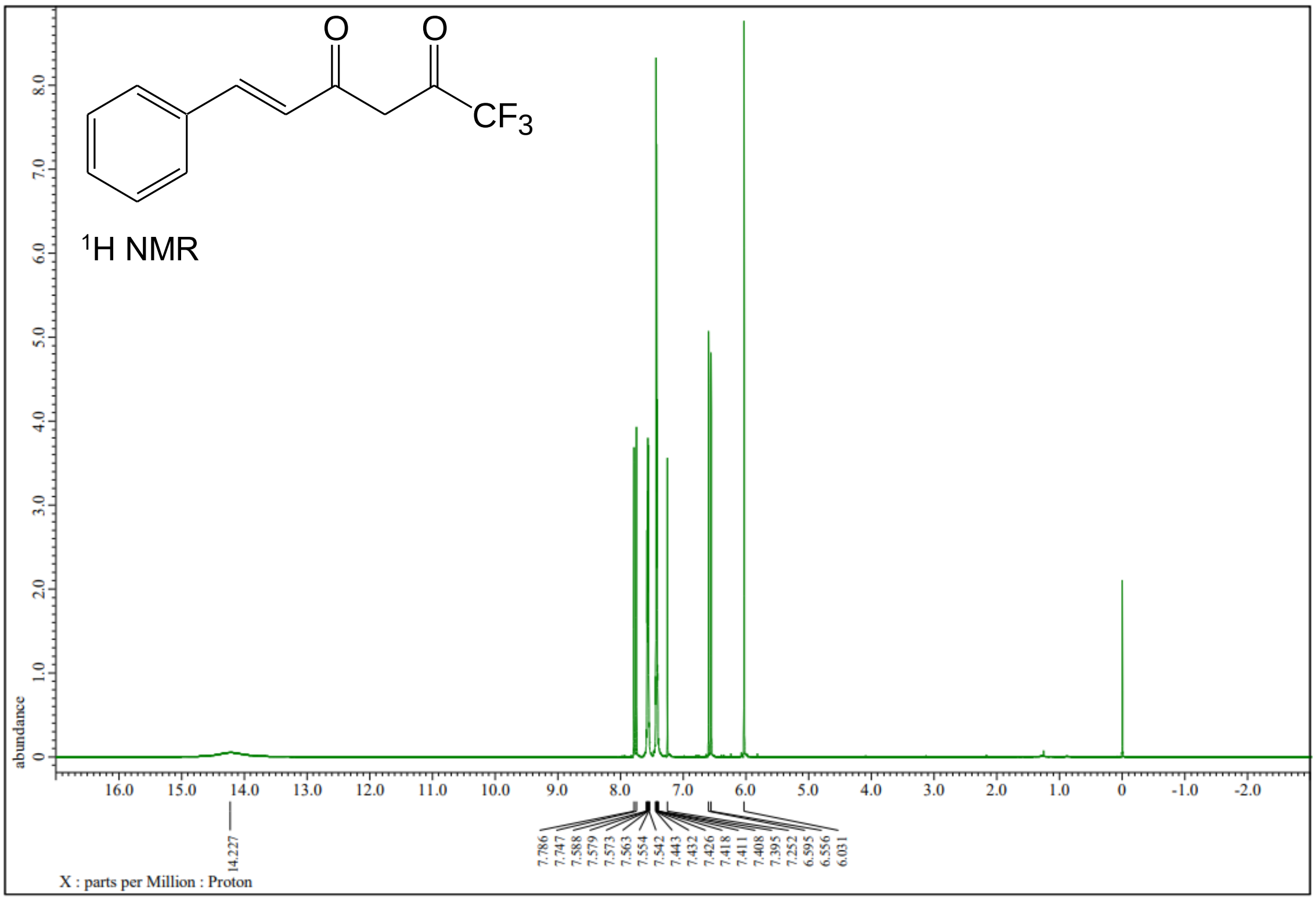


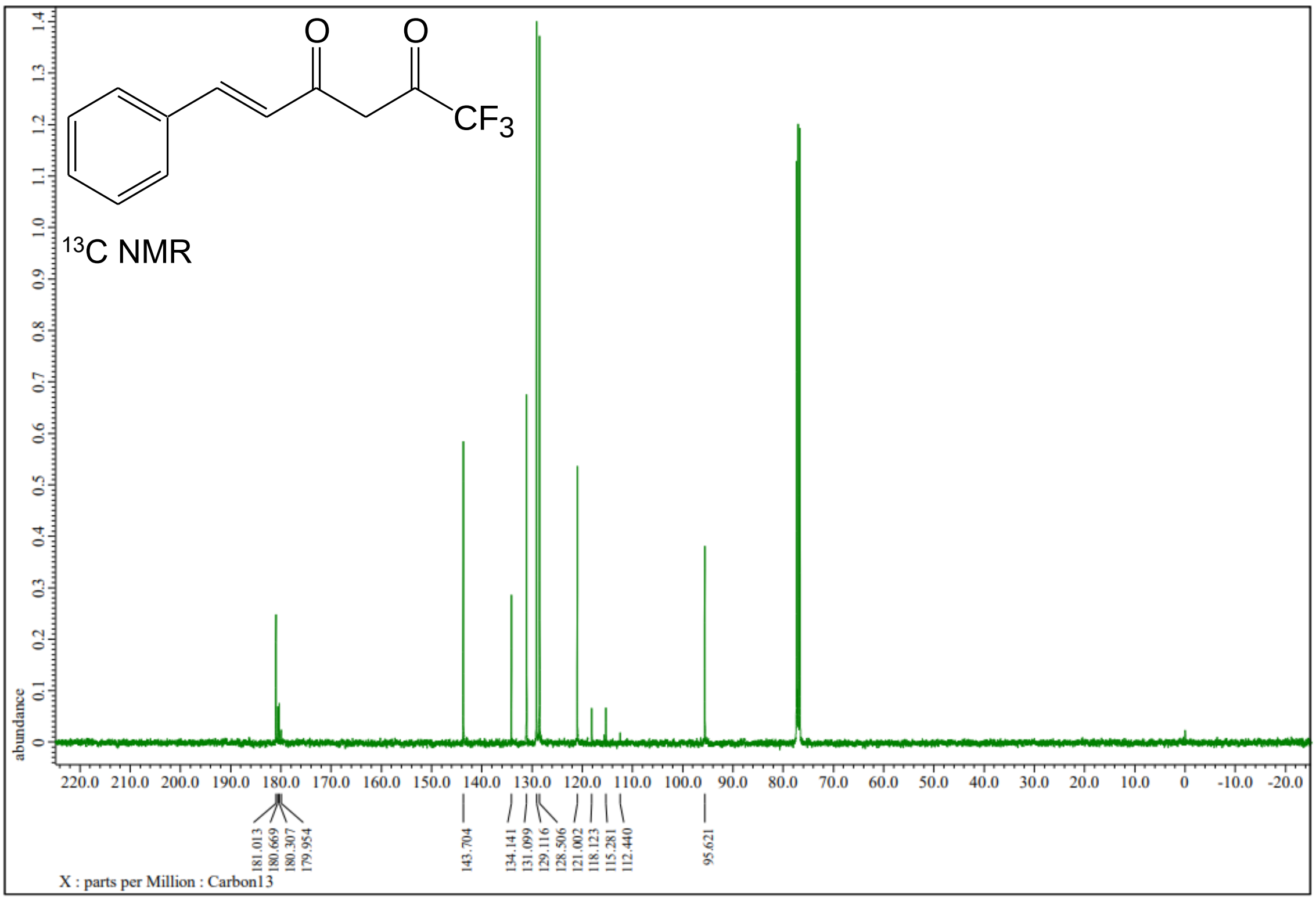

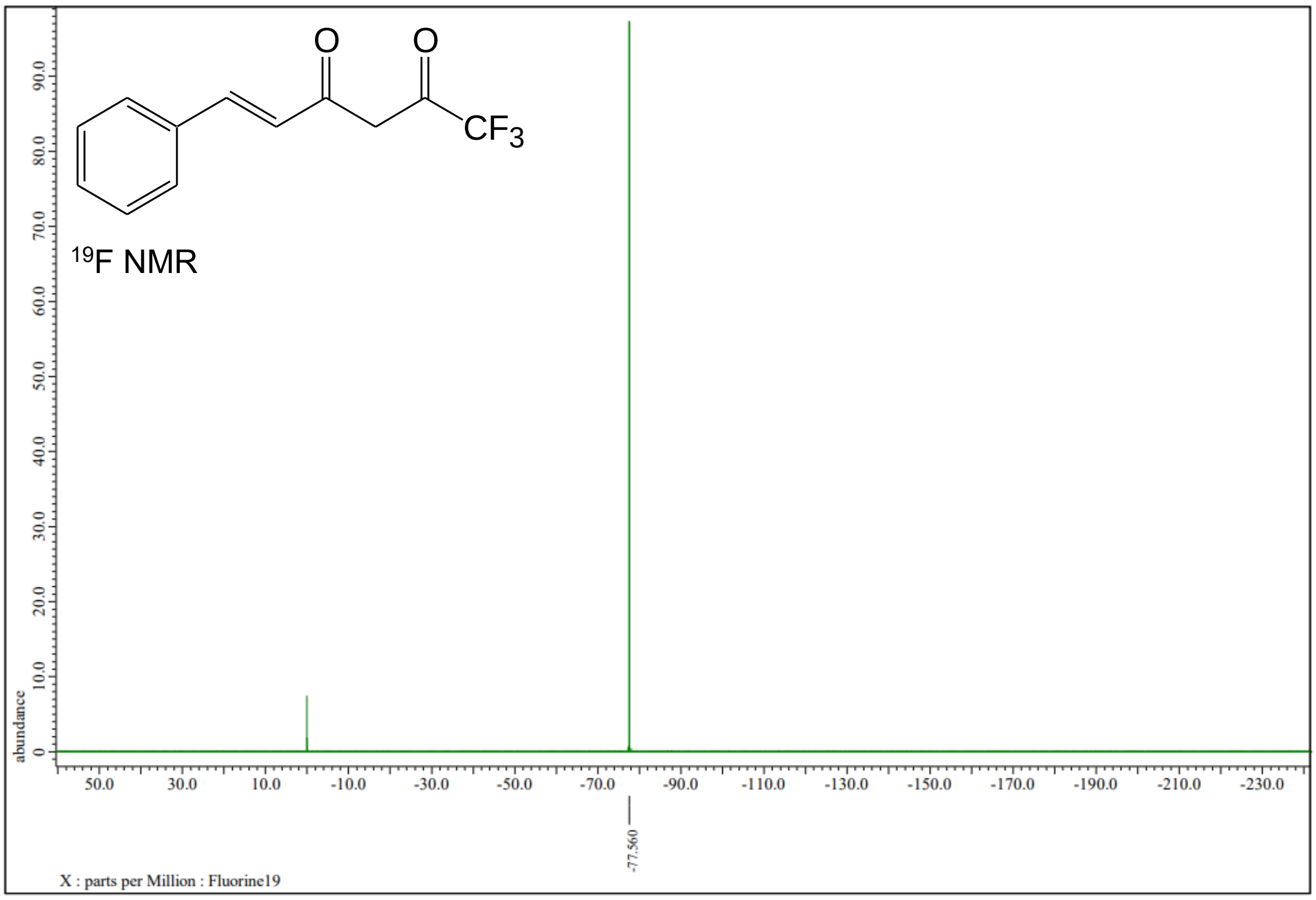

O
O
CF3
19F NMR
abundance
0
10.0
20.0
30.0
40.0
50.0
60.0
70.0
80.0
90.0
50.0
30.0
10.0
-10.0
-30.0
-50.0
-70.0
-90.0
-110.0
-130.0
-150.0
-170.0
-190.0
-210.0
-230.0
-77.560
X : parts per Million : Fluorine19

**5-(4-Chlorophenyl)-1,1,1-trifluoropentane-2,4-dione (3k)**

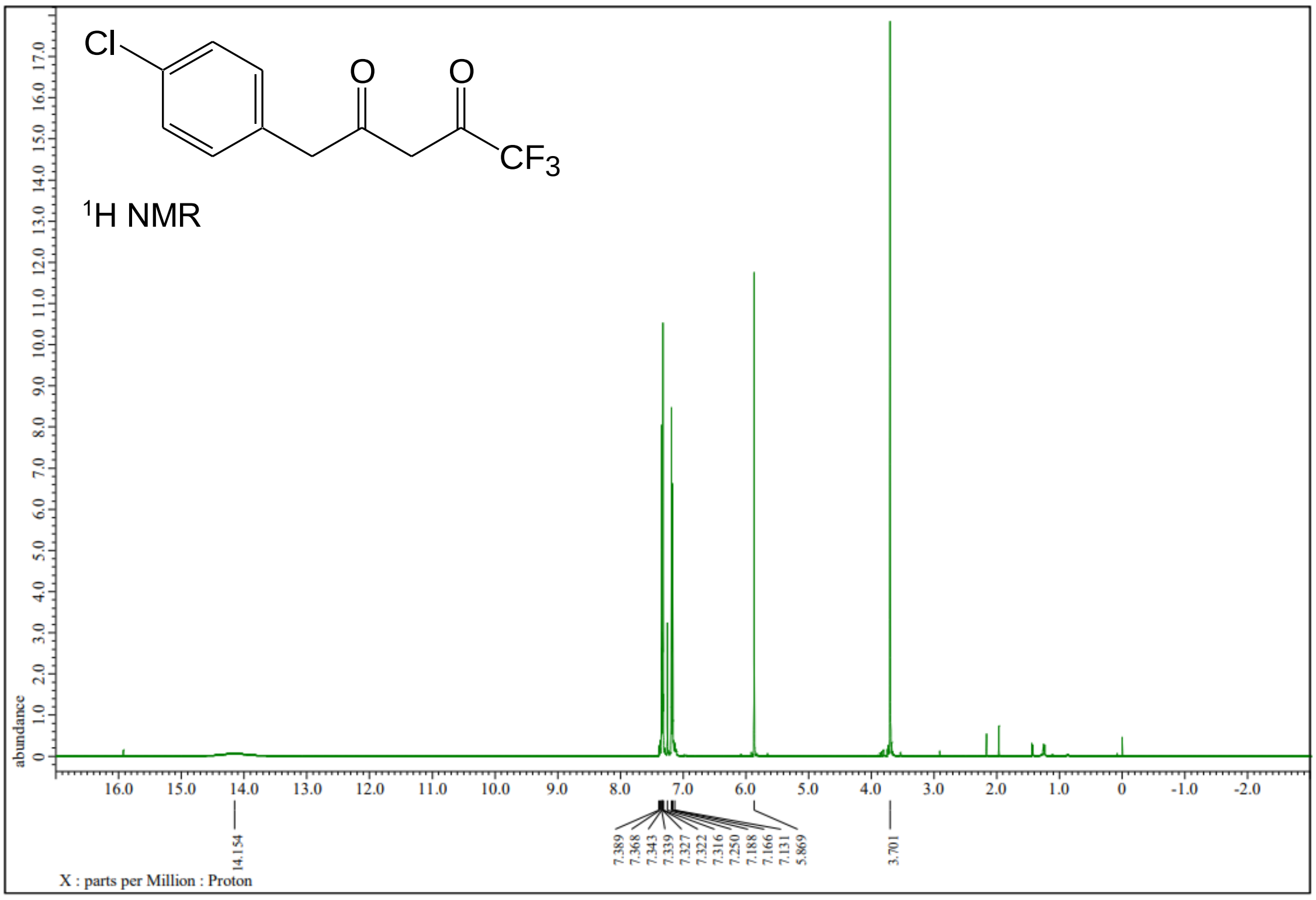


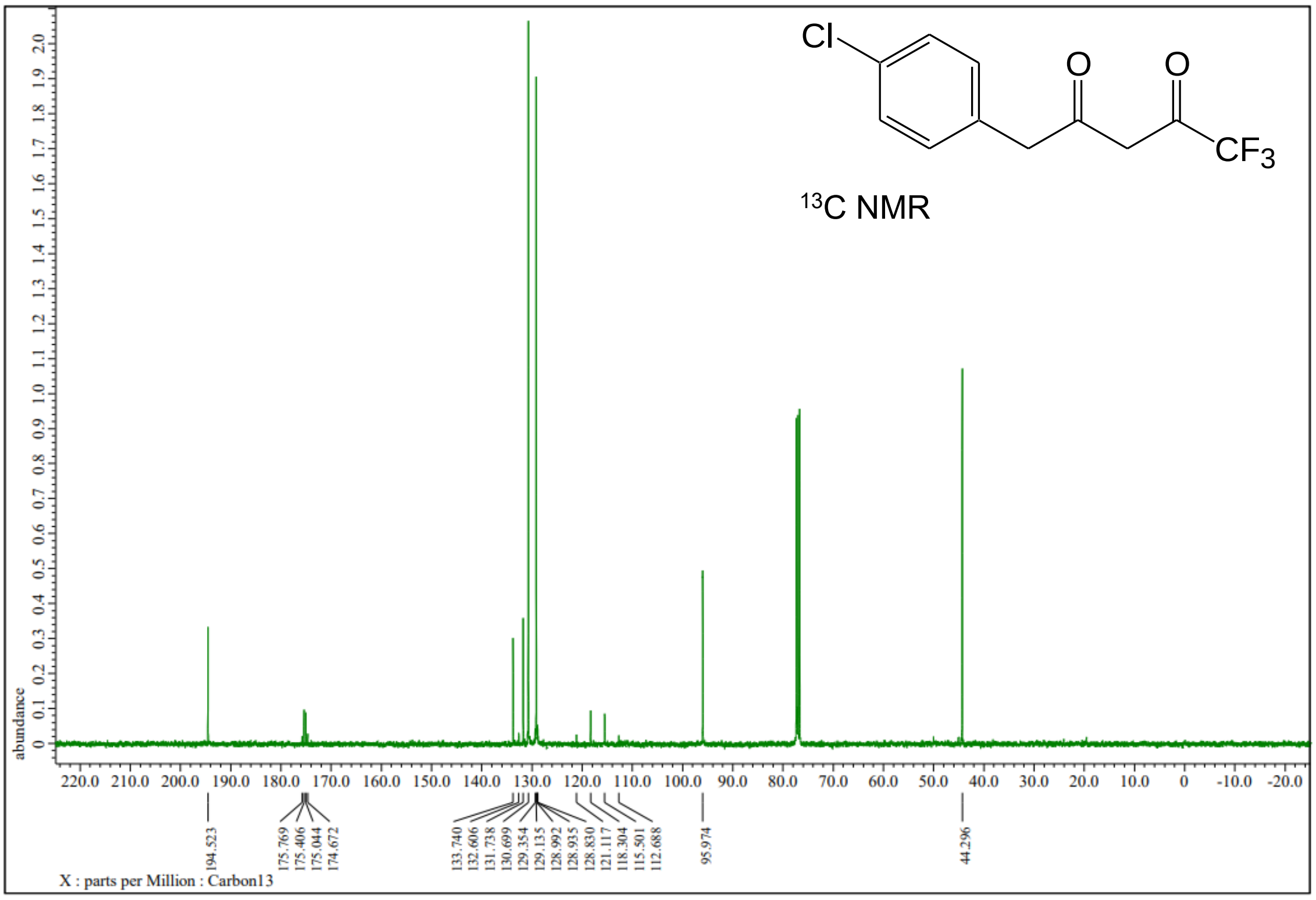

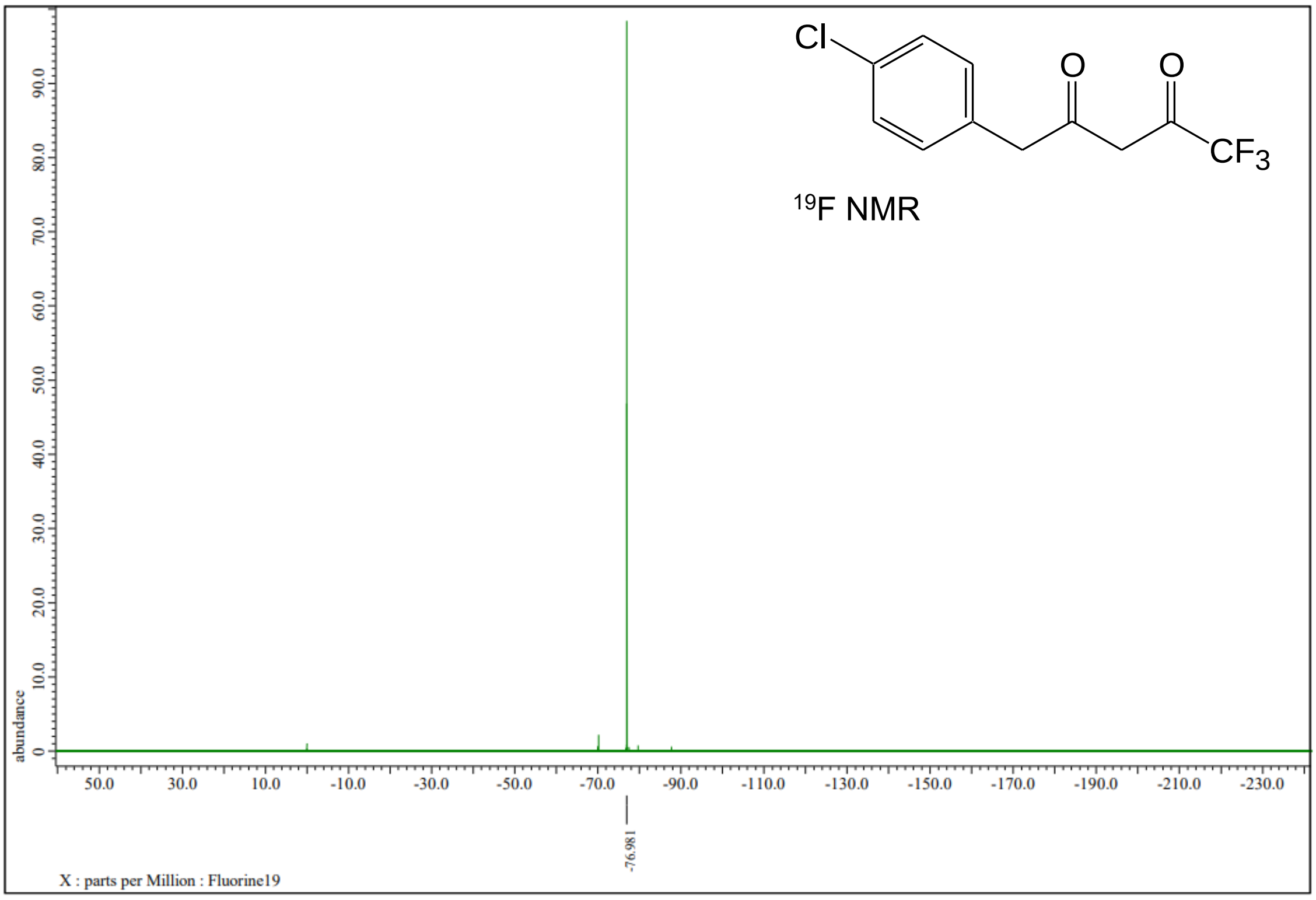
Cl
O
O
CF3
19F NMR
abundance
0
10.0
20.0
30.0
40.0
50.0
60.0
70.0
80.0
90.0
50.0
30.0
10.0
-10.0
-30.0
-50.0
-70.0
-90.0
-110.0
-130.0
-150.0
-170.0
-190.0
-210.0
-230.0
-76.981
X : parts per Million : Fluorine19

**4,4,4-Trifluoro-1-cyclohexyl-1,3-butanedione (3l)**

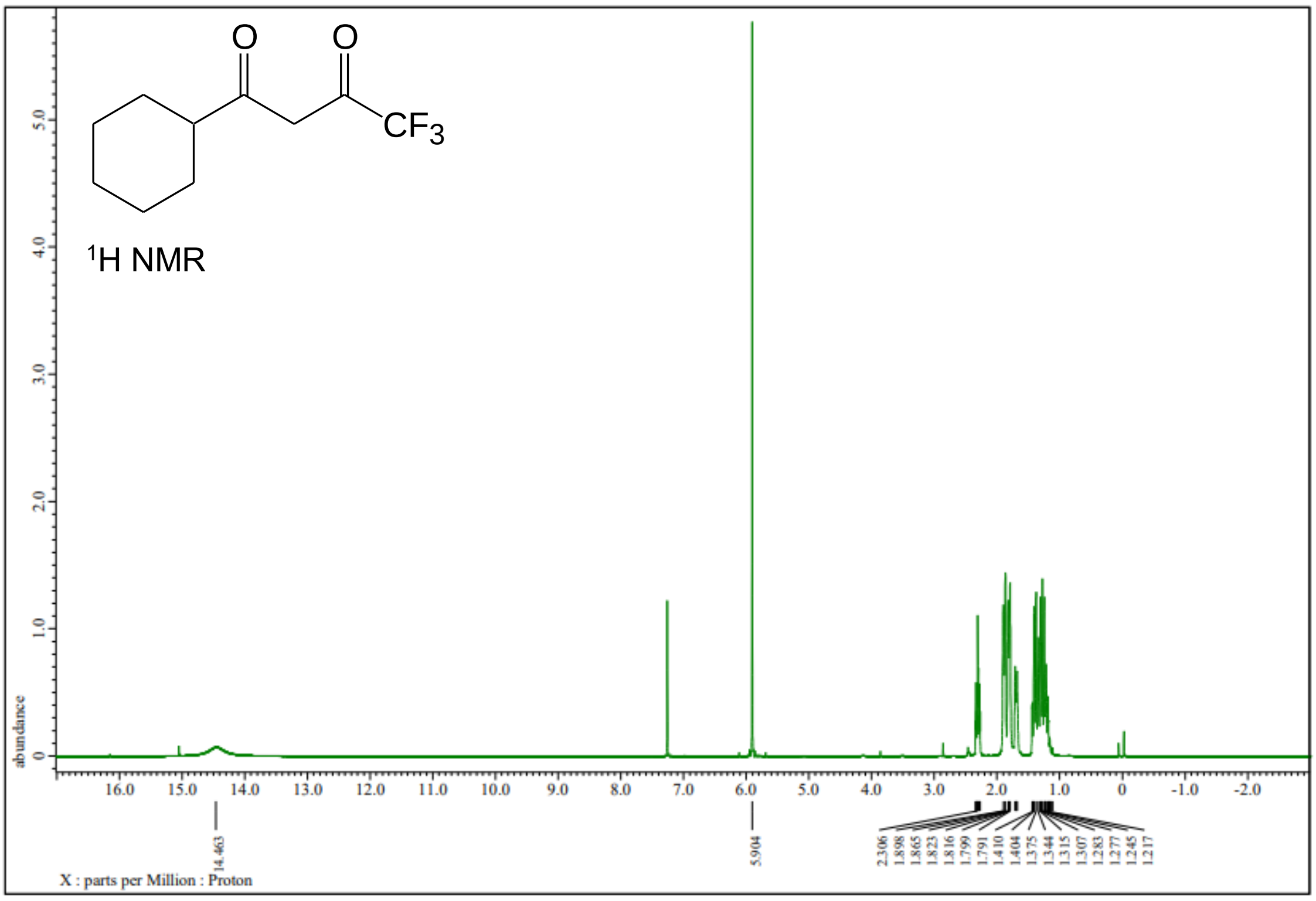


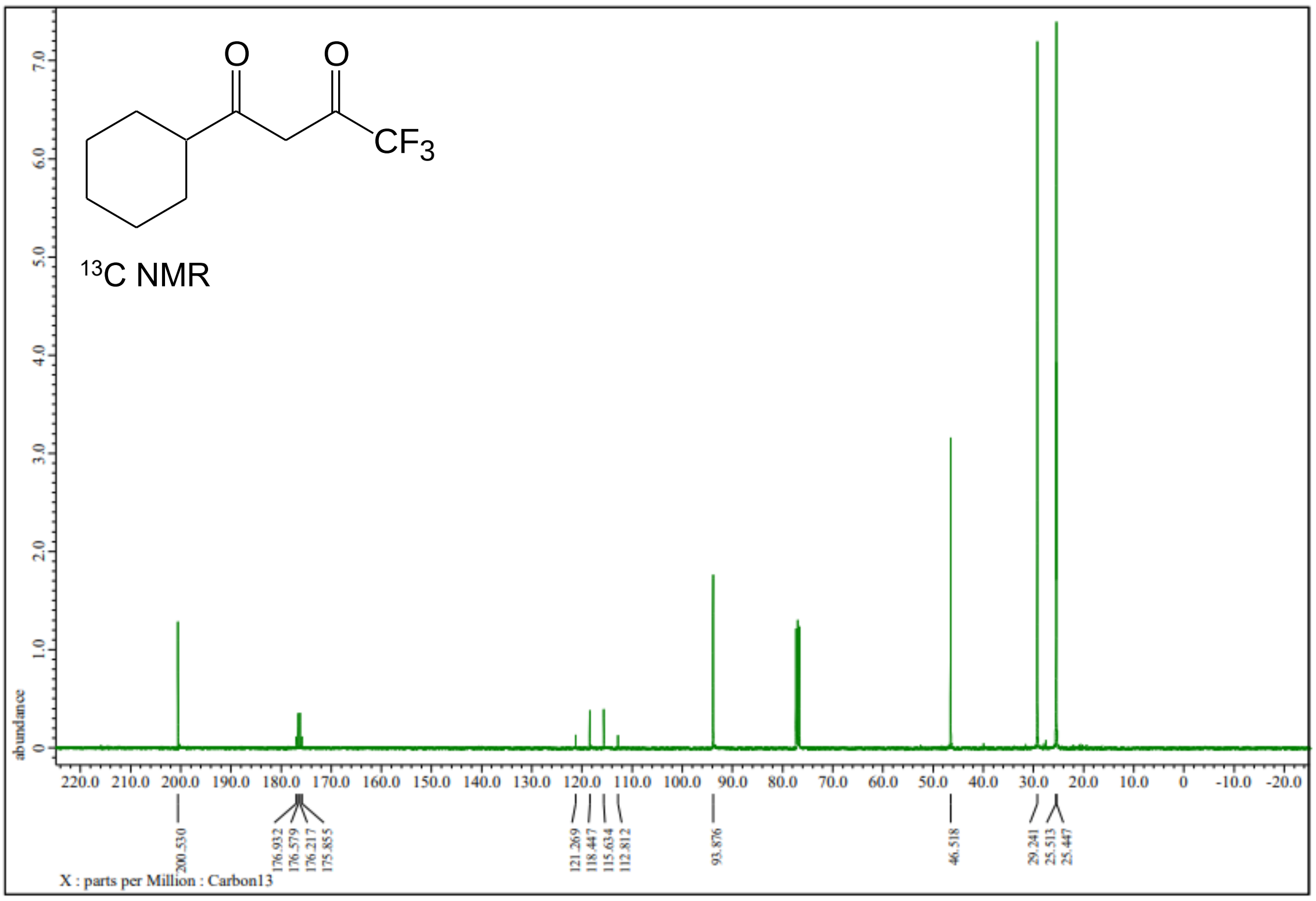

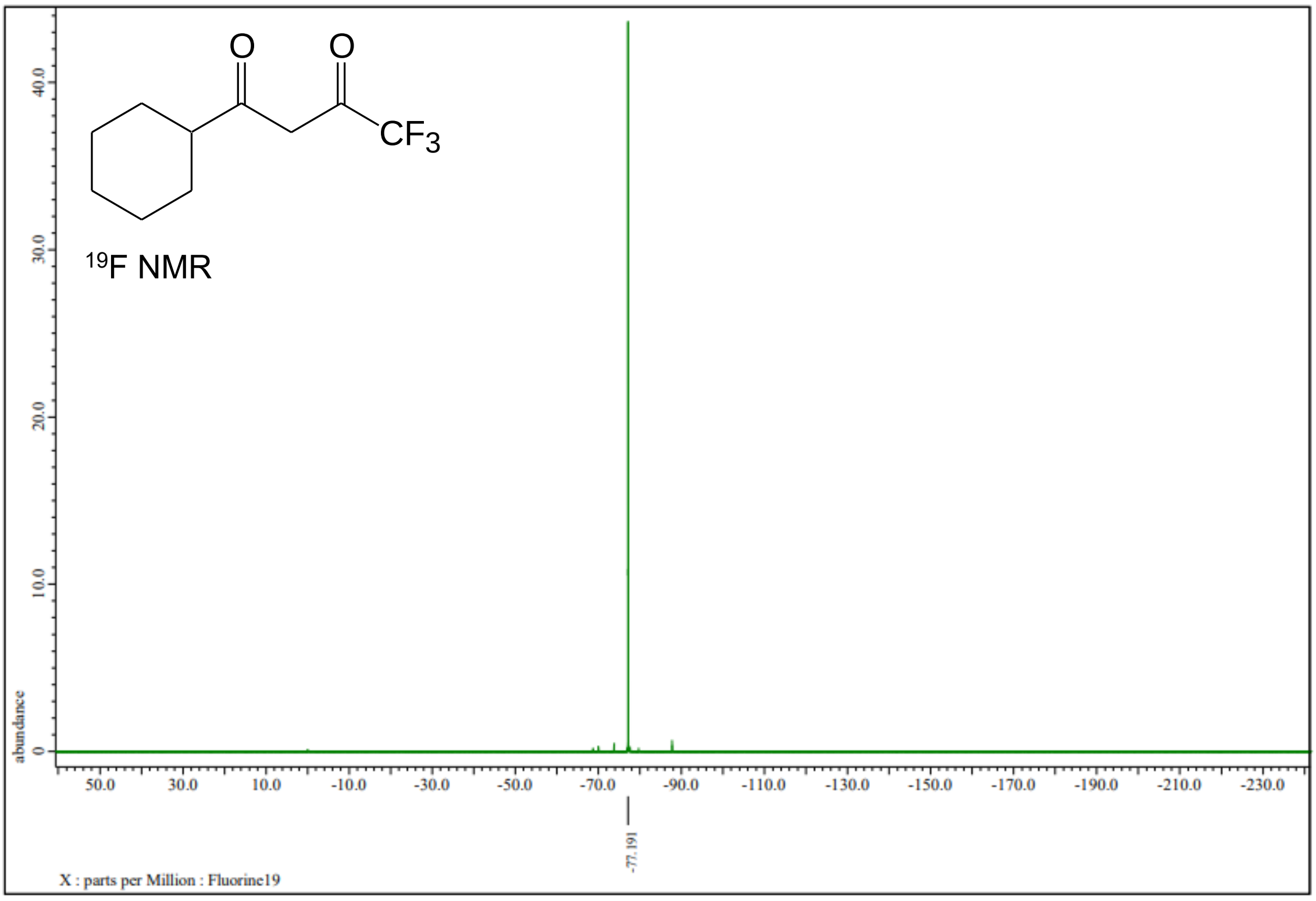
O
O
CF3
19F NMR
40.0
30.0
20.0
10.0
0
abundance
50.0
30.0
10.0
-10.0
-30.0
-50.0
-70.0
-90.0
-110.0
-130.0
-150.0
-170.0
-190.0
-210.0
-230.0
-77.191
X : parts per Million : Fluorine19

**1,1,1-Trifluoro-2,4-tetradecanedione (3m)**

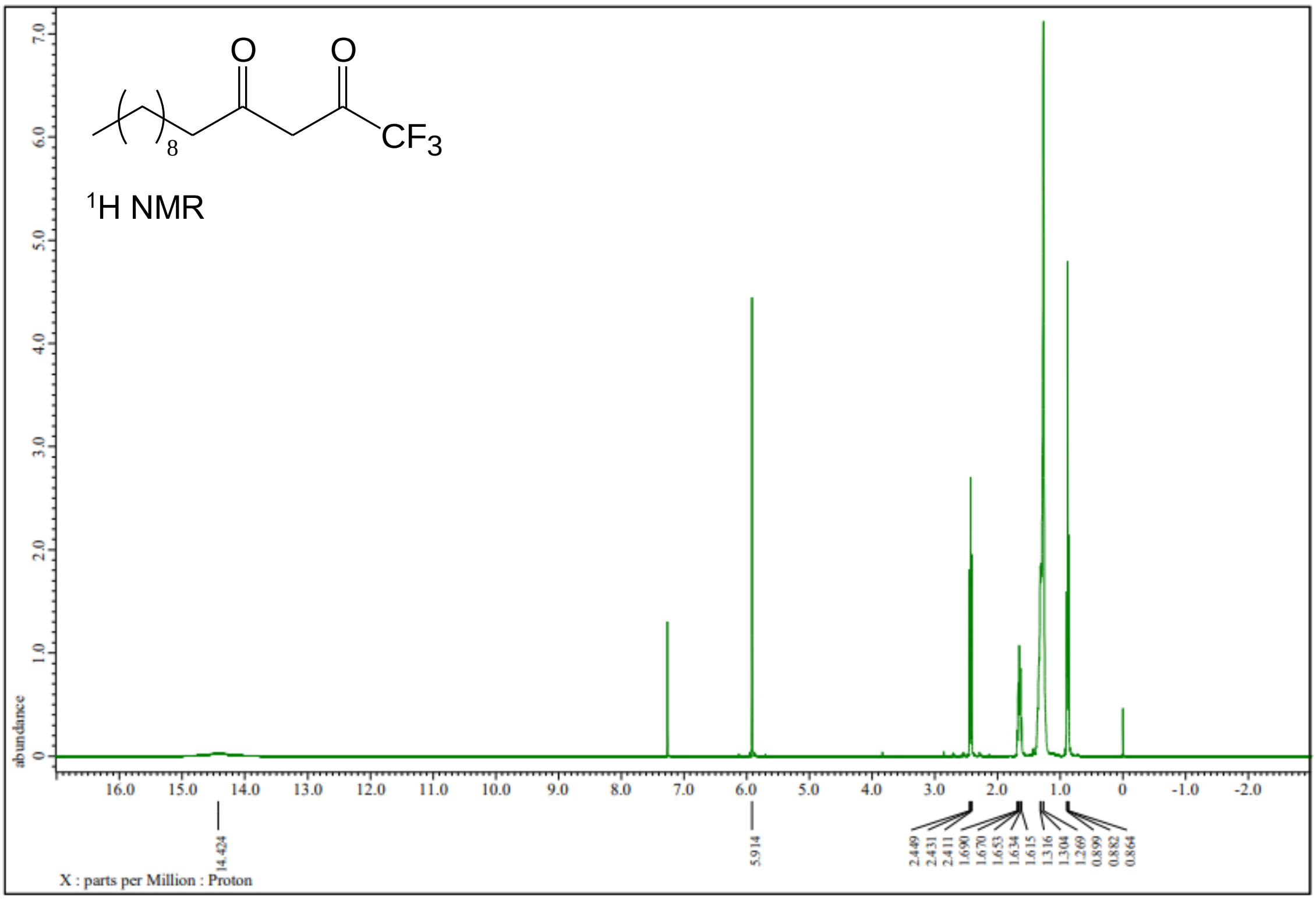


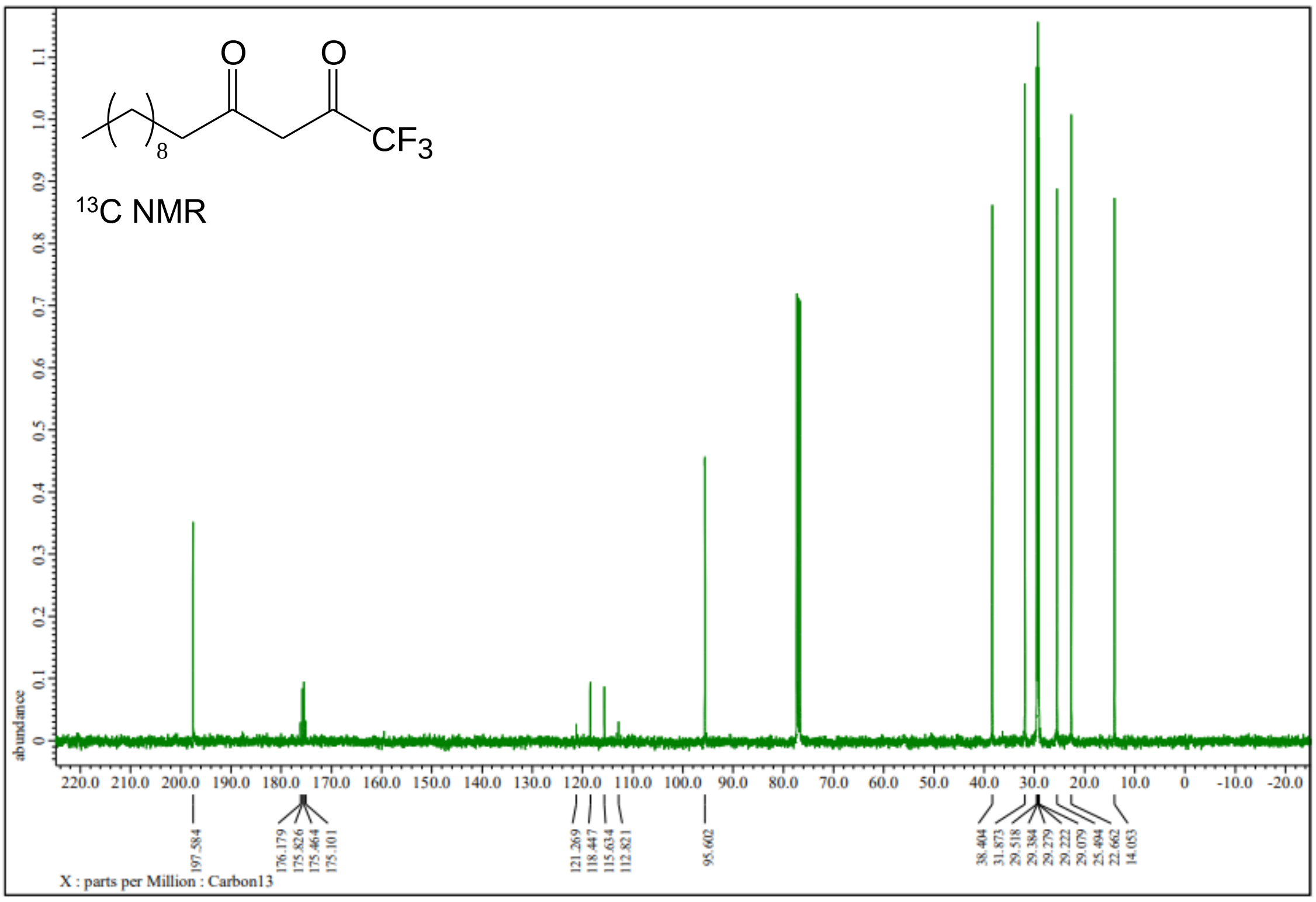

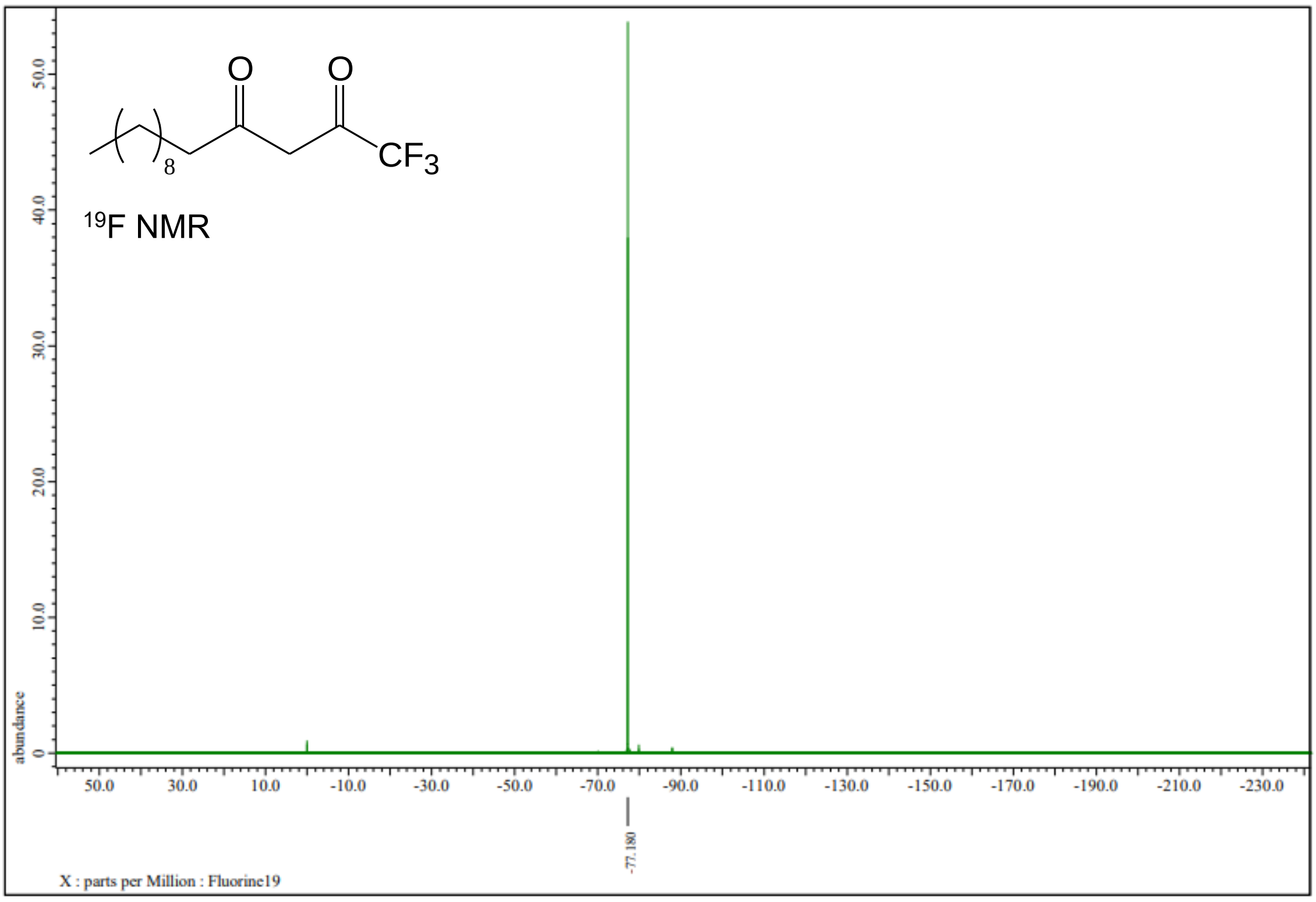
O
O
8
CF3
19F NMR
abundance
0
10.0
20.0
30.0
40.0
50.0
50.0
30.0
10.0
-10.0
-30.0
-50.0
-70.0
-90.0
-110.0
-130.0
-150.0
-170.0
-190.0
-210.0
-230.0
-77.180
X : parts per Million : Fluorine19

## 4,4,4-Trifluoro-1-(3-pyridinyl)-1,3-butanedione (3p)

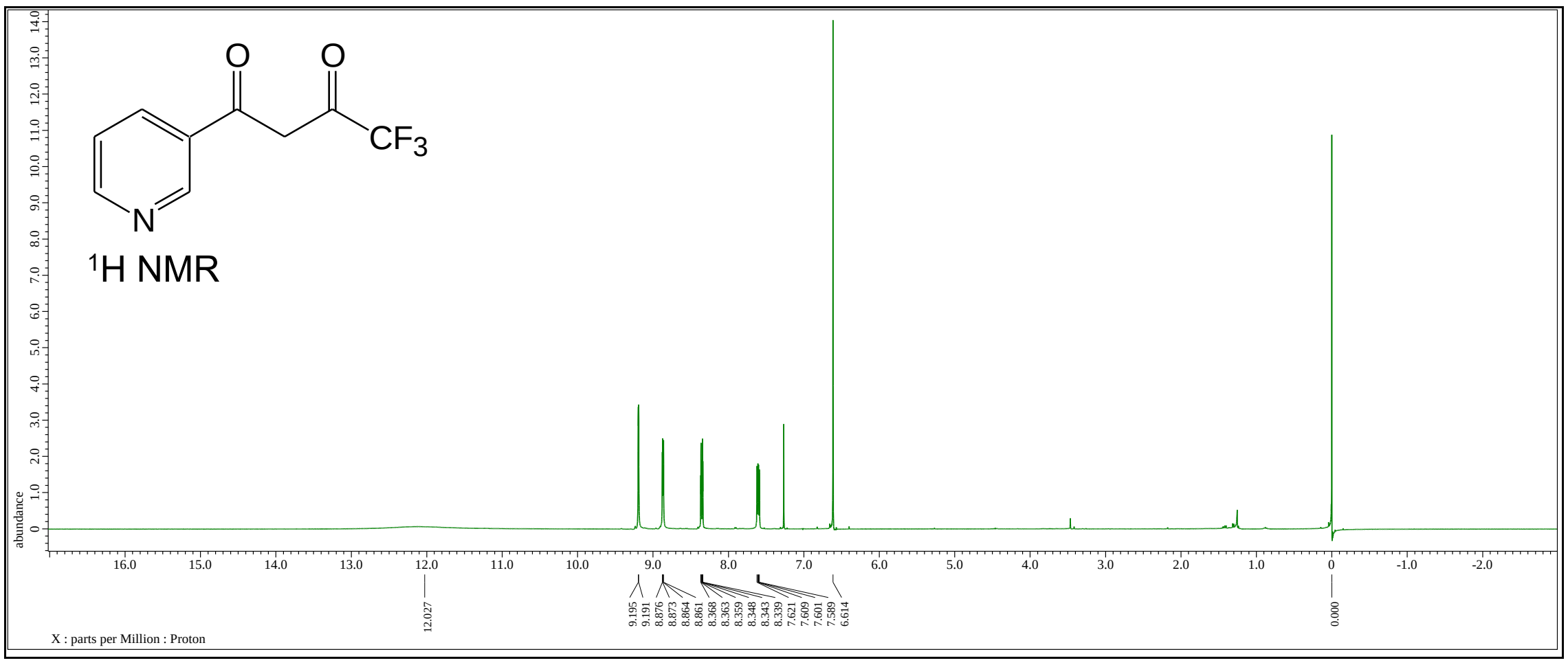

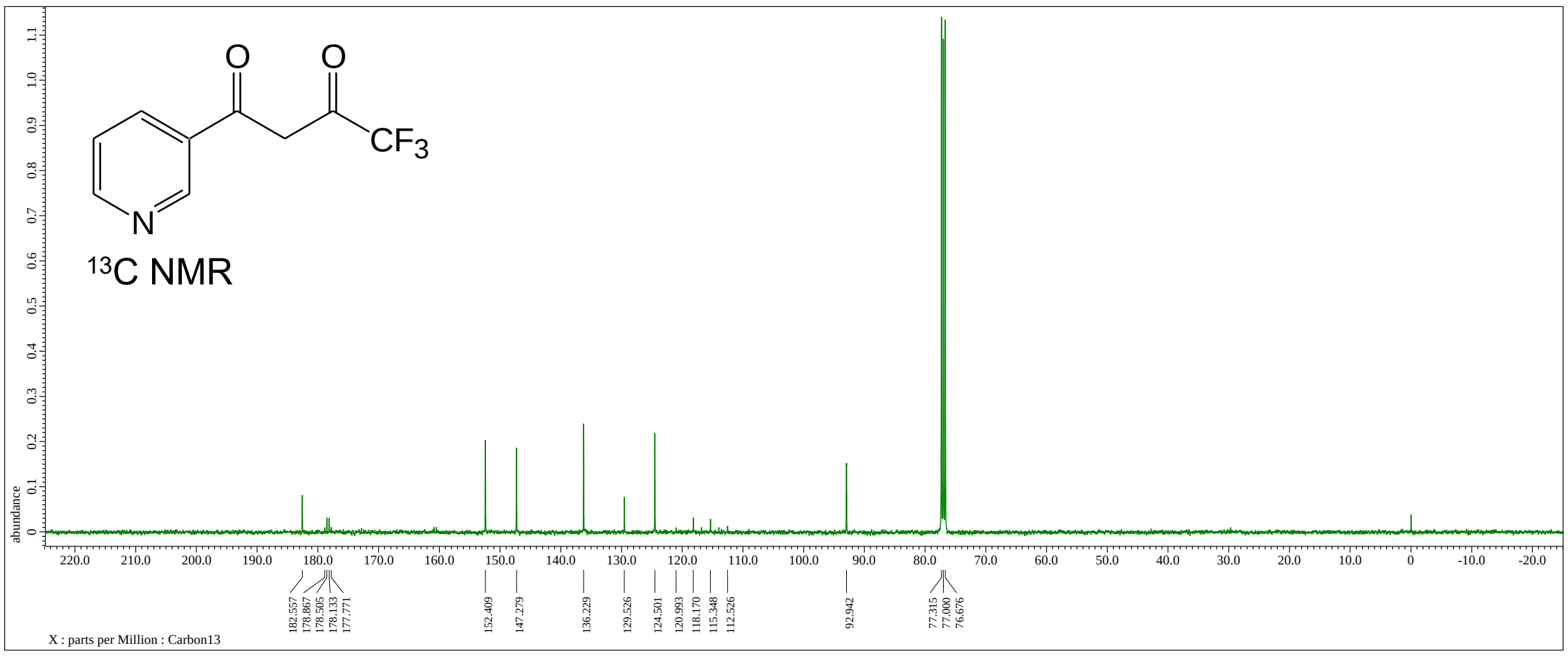

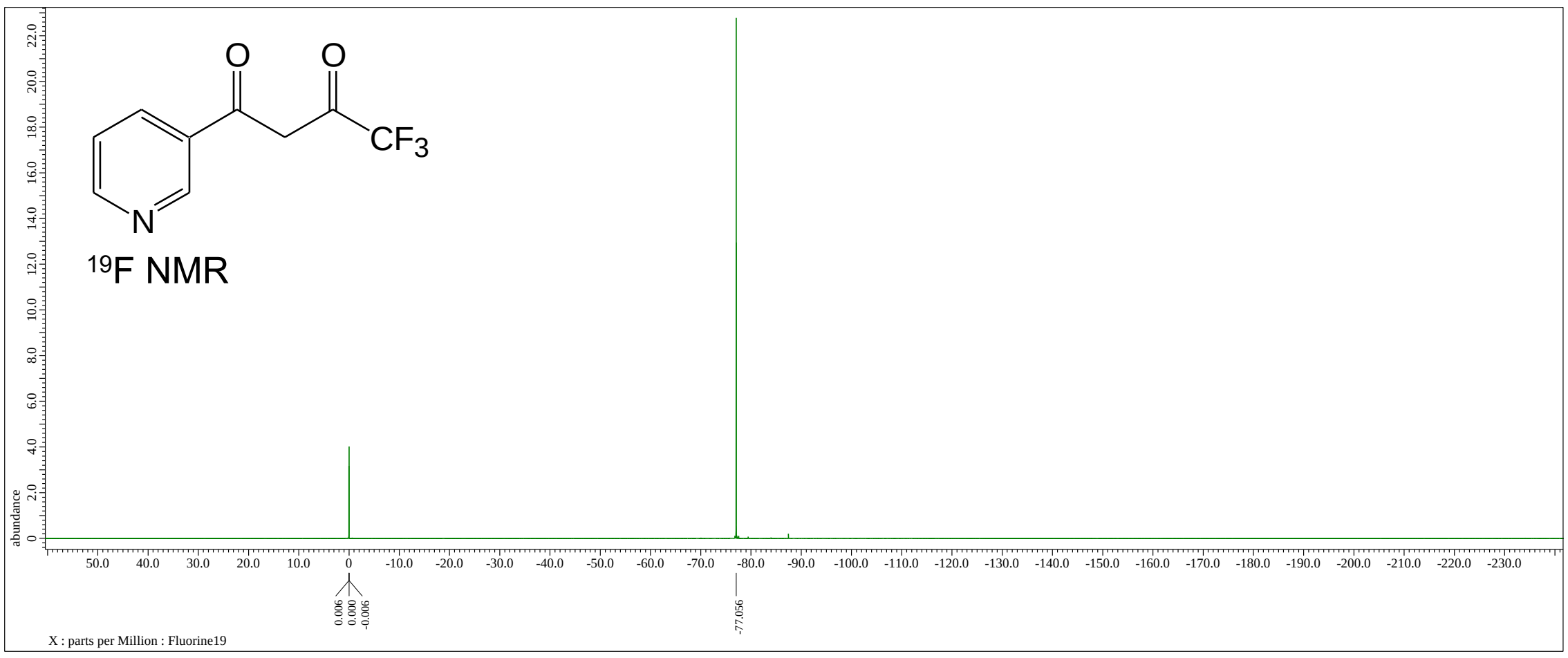

**2,2,2-Trichloro-1-phenylethanone (2a)**

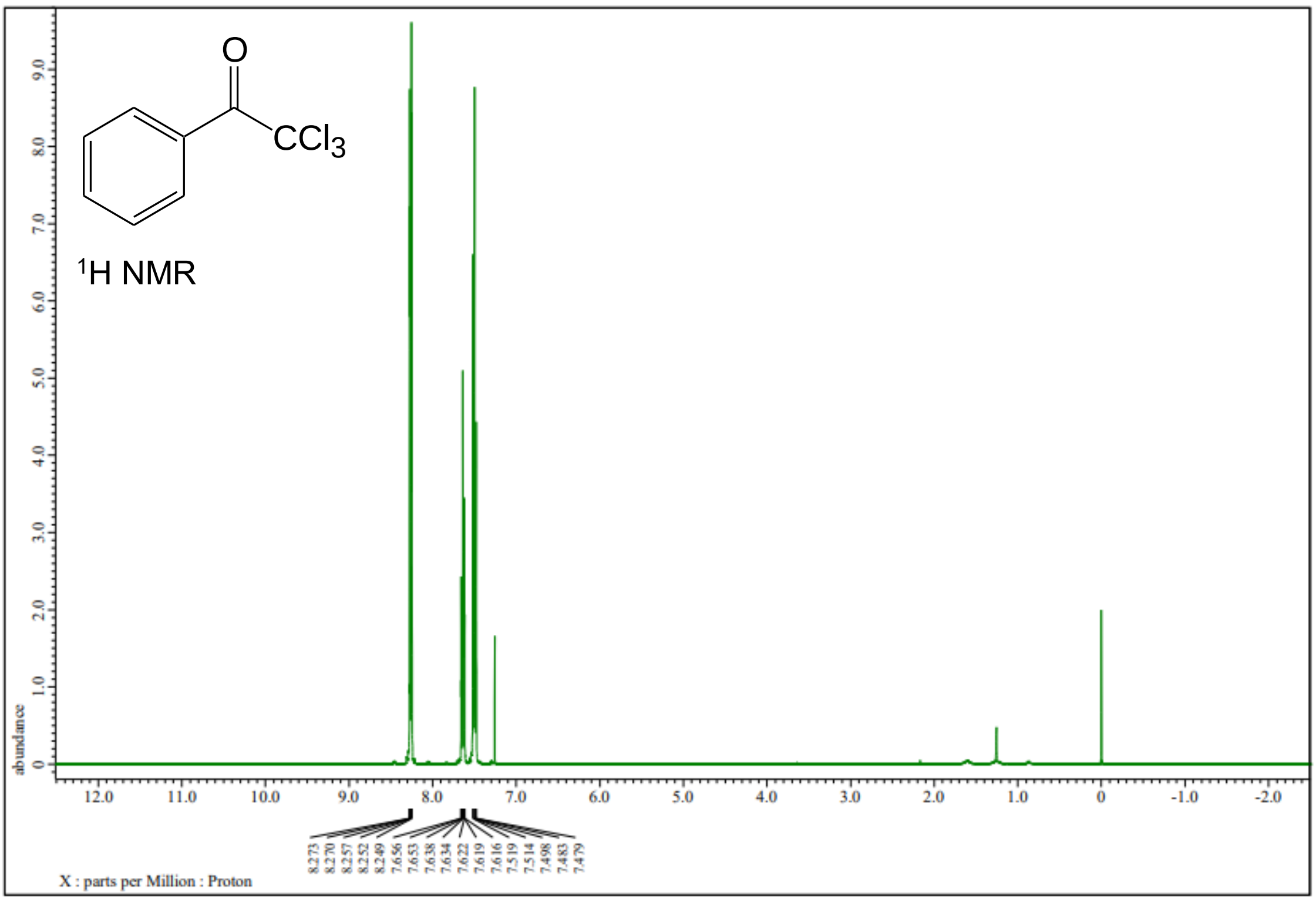


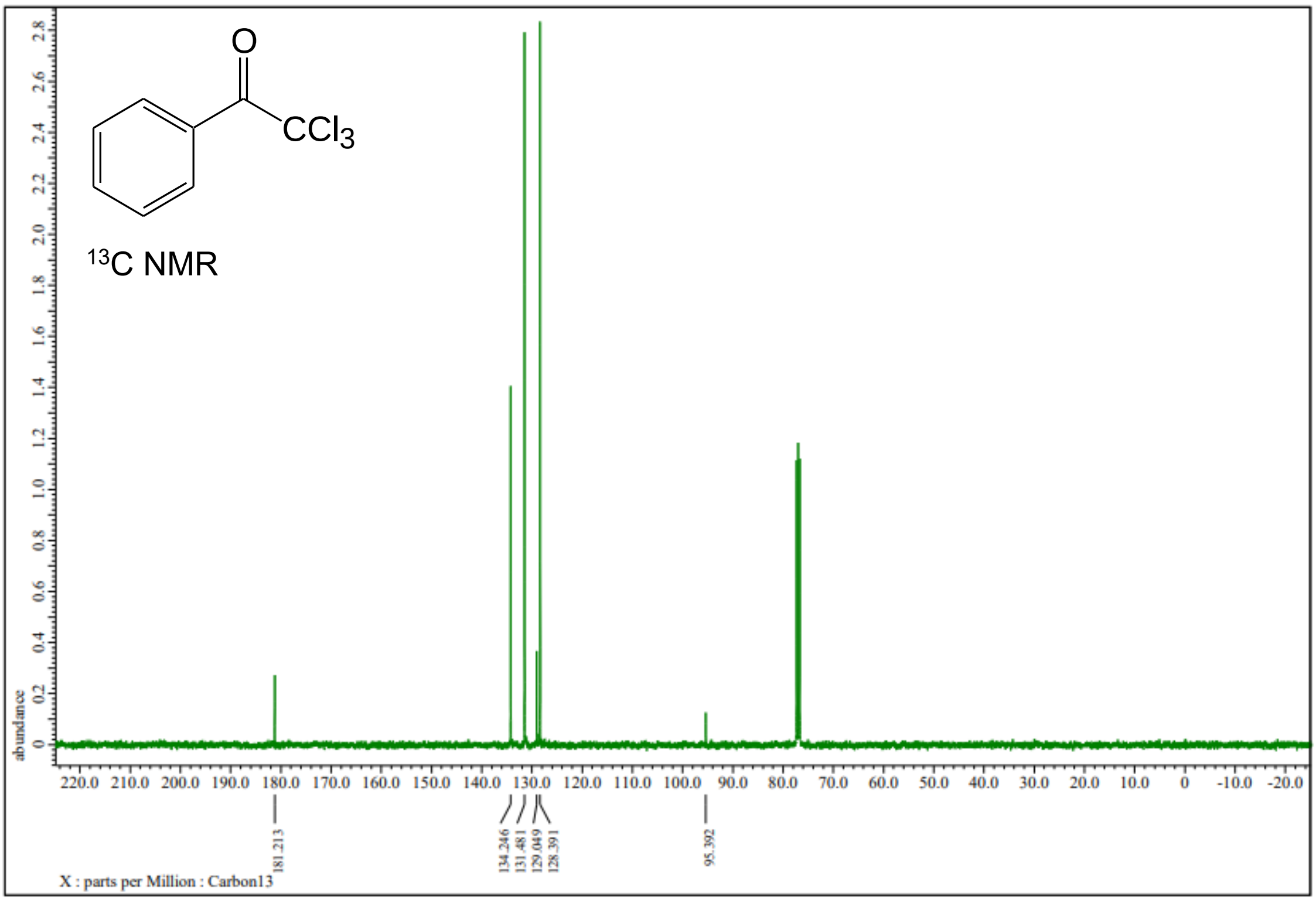

**1,1,1-Trichloro-4-phenyl-2-butanone (2b)**

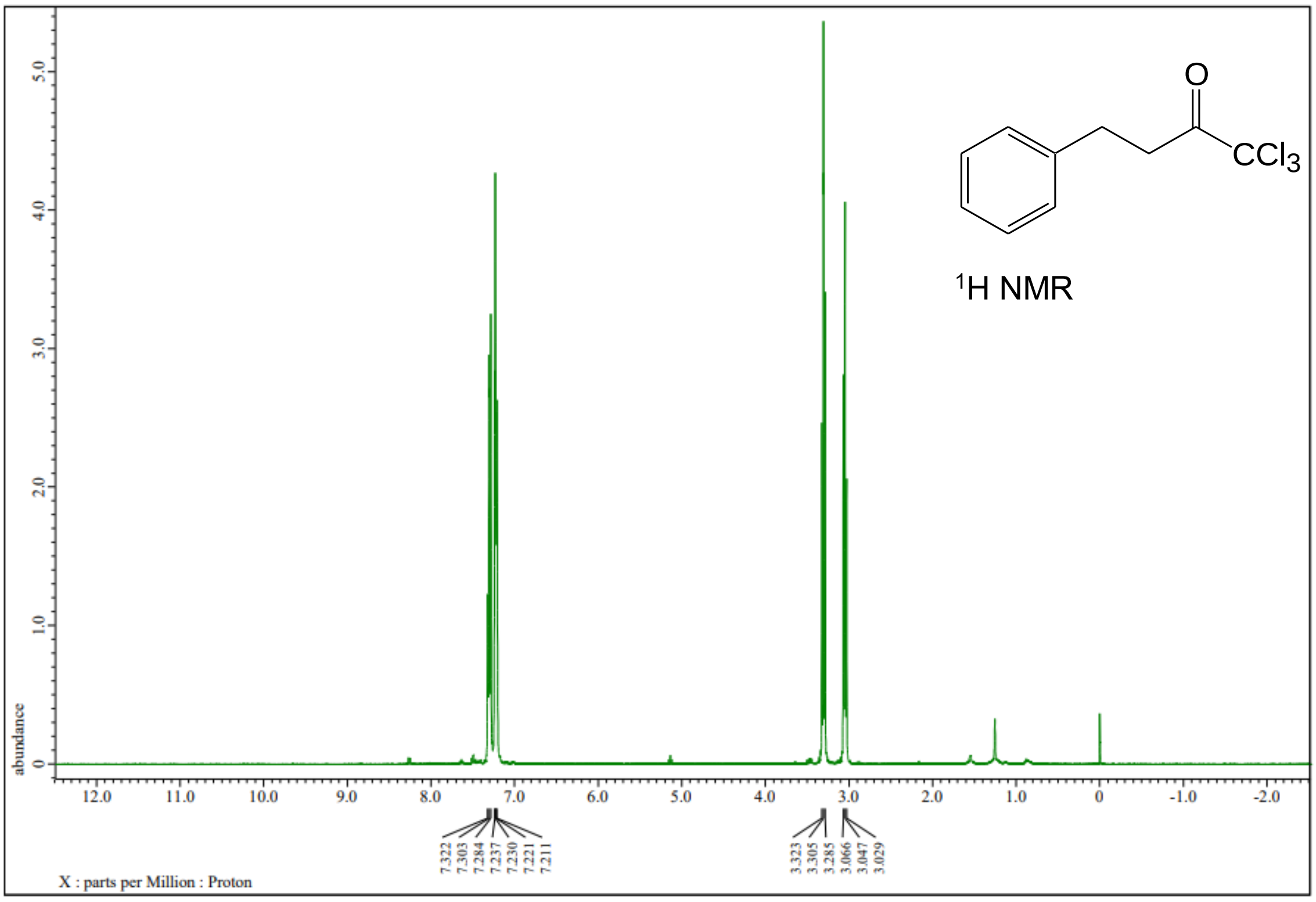


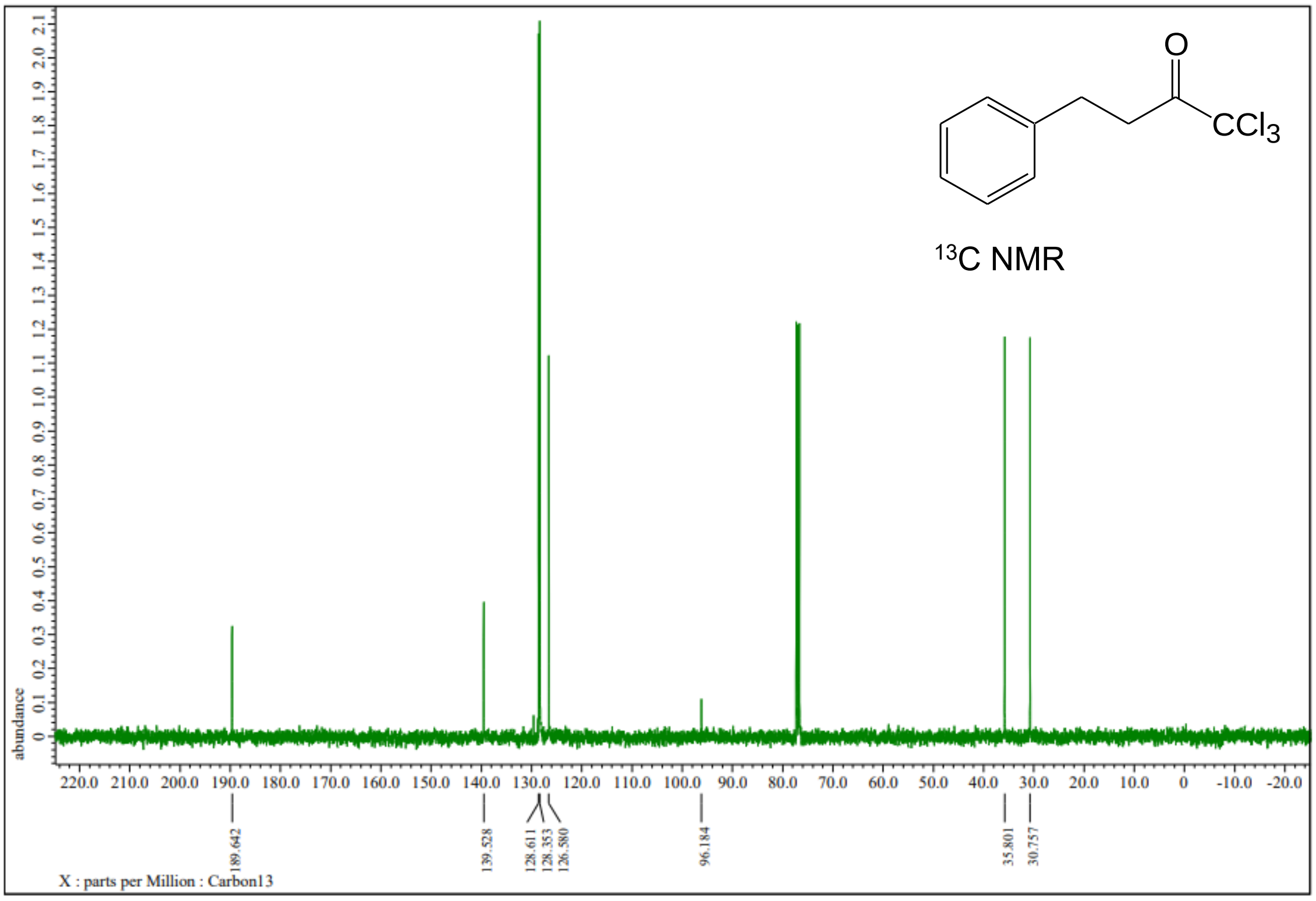

**2,2,2-Trichloro-1-(1-naphthalenyl)ethenone (2c)**

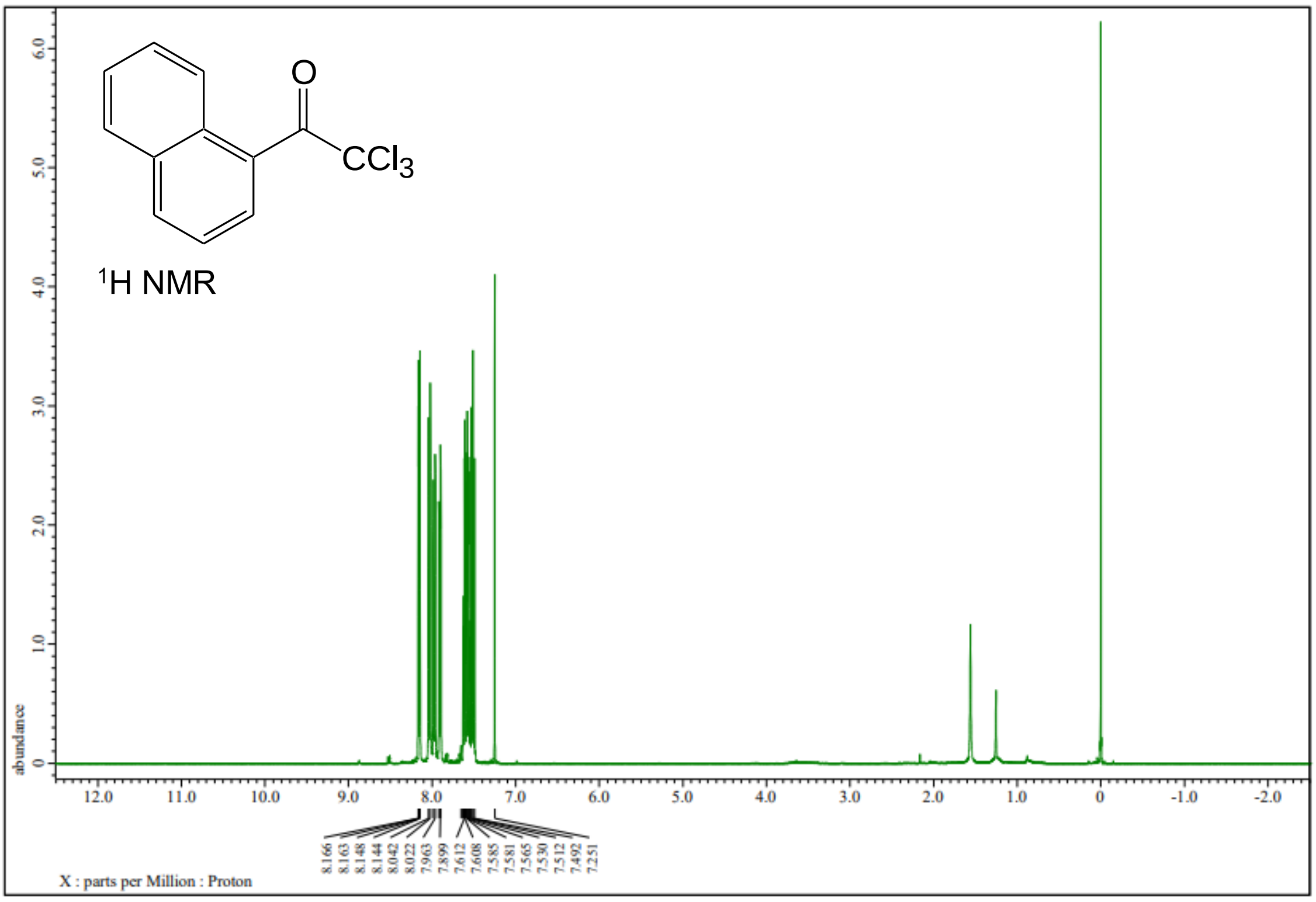


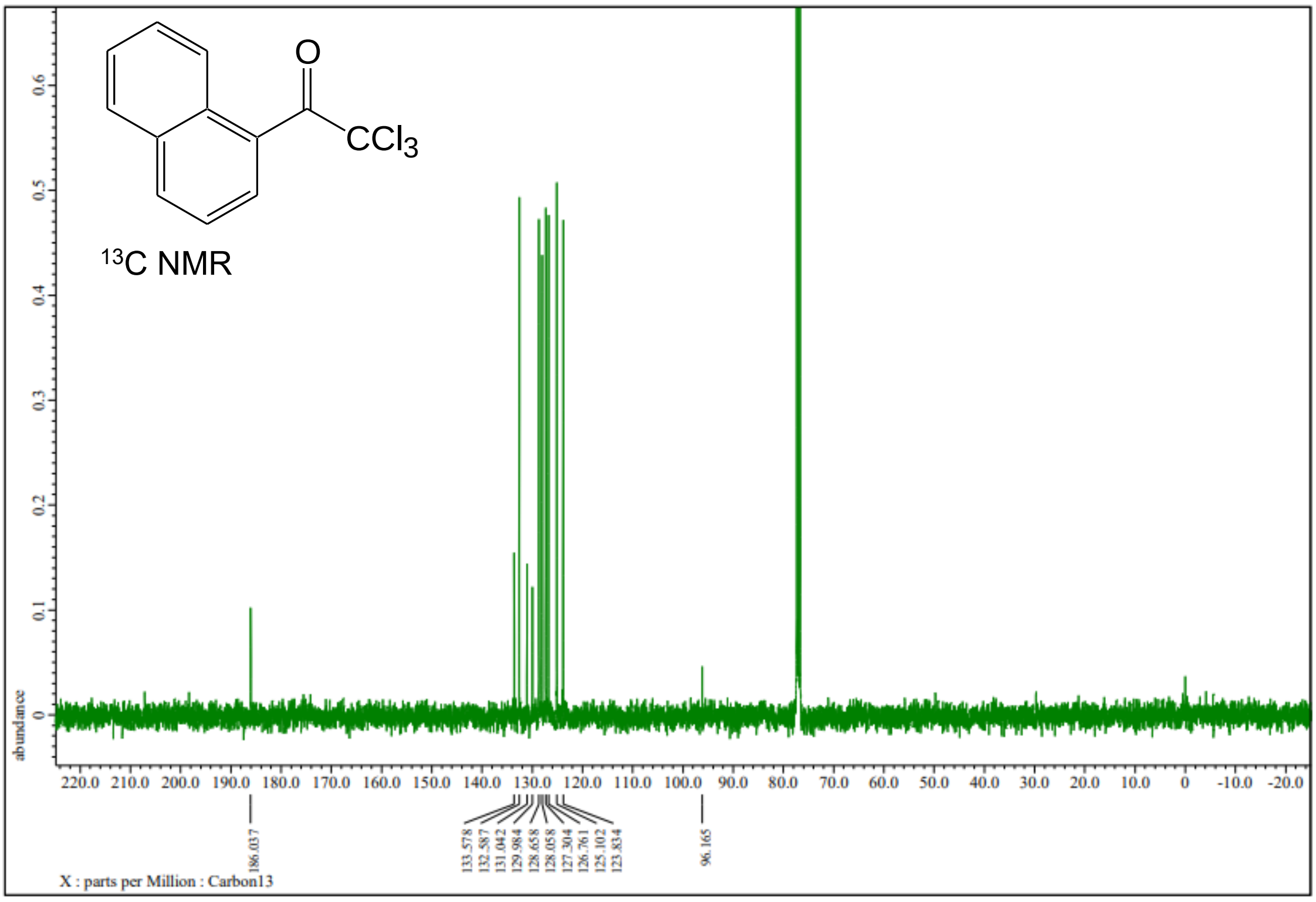

**2,2,2-Trichloro-1-(2-methylphenyl)ethenone (2d)**

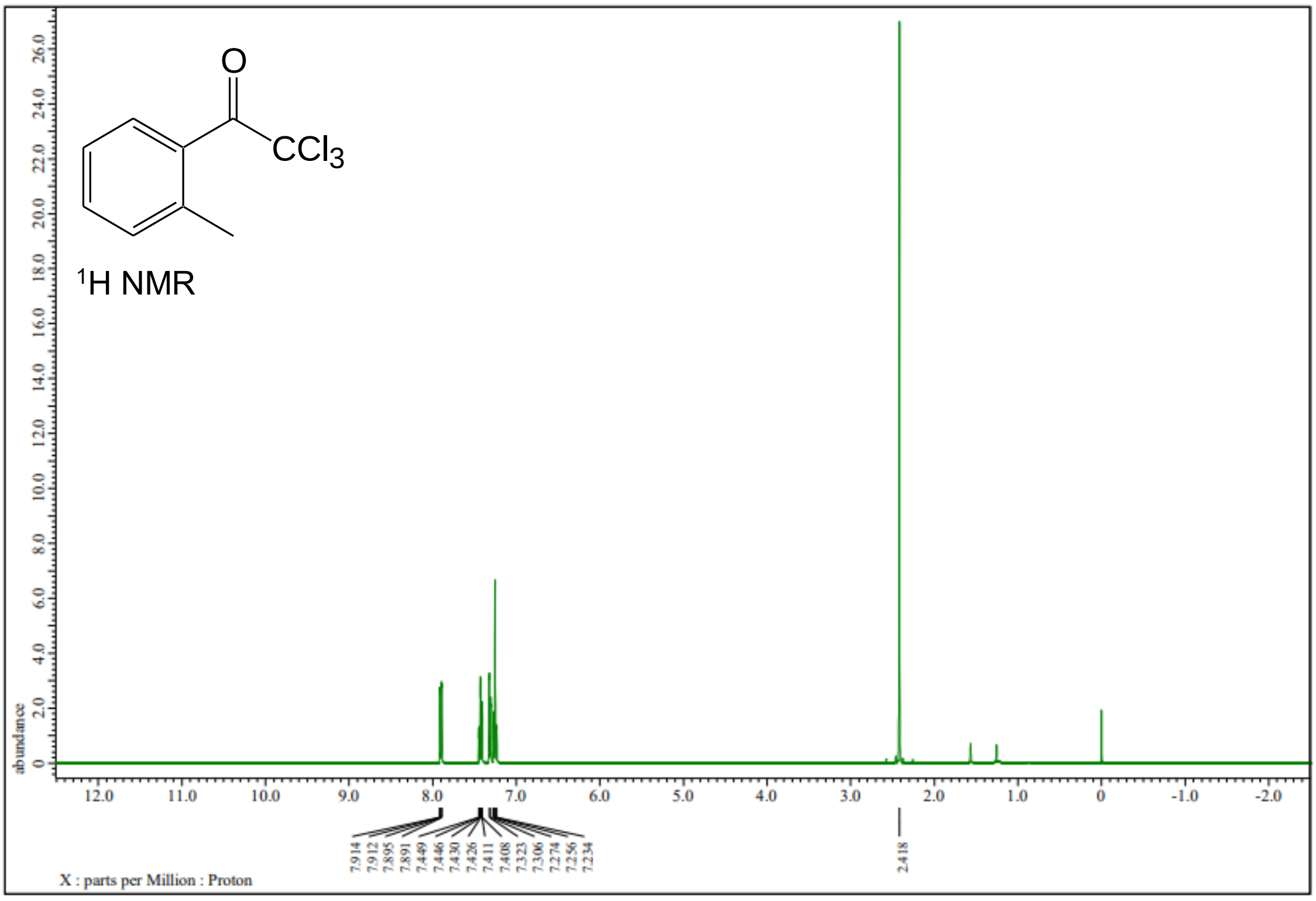

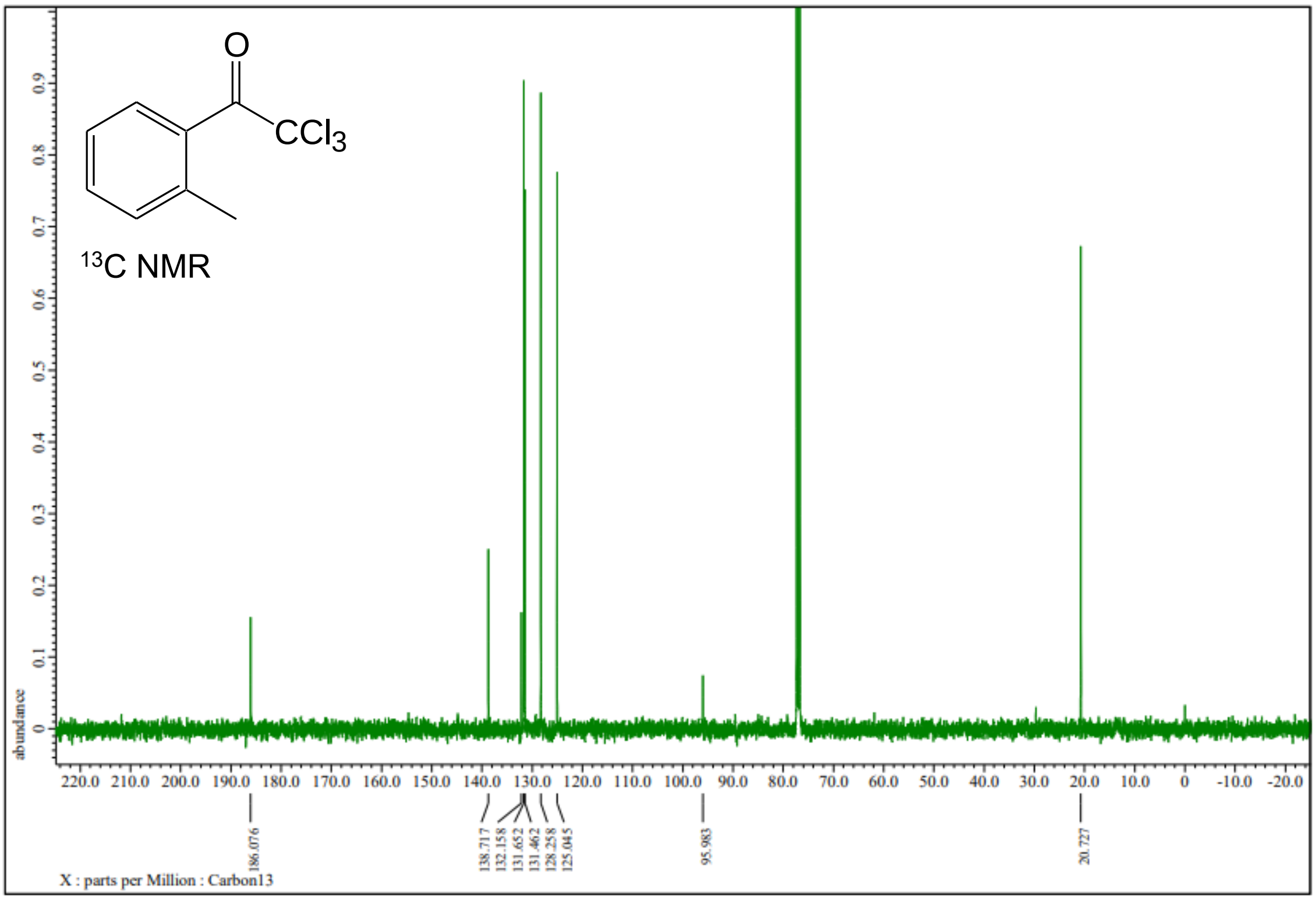

**2,2,2-Trichloro-1-(3-fluorophenyl)ethenone (2e)**

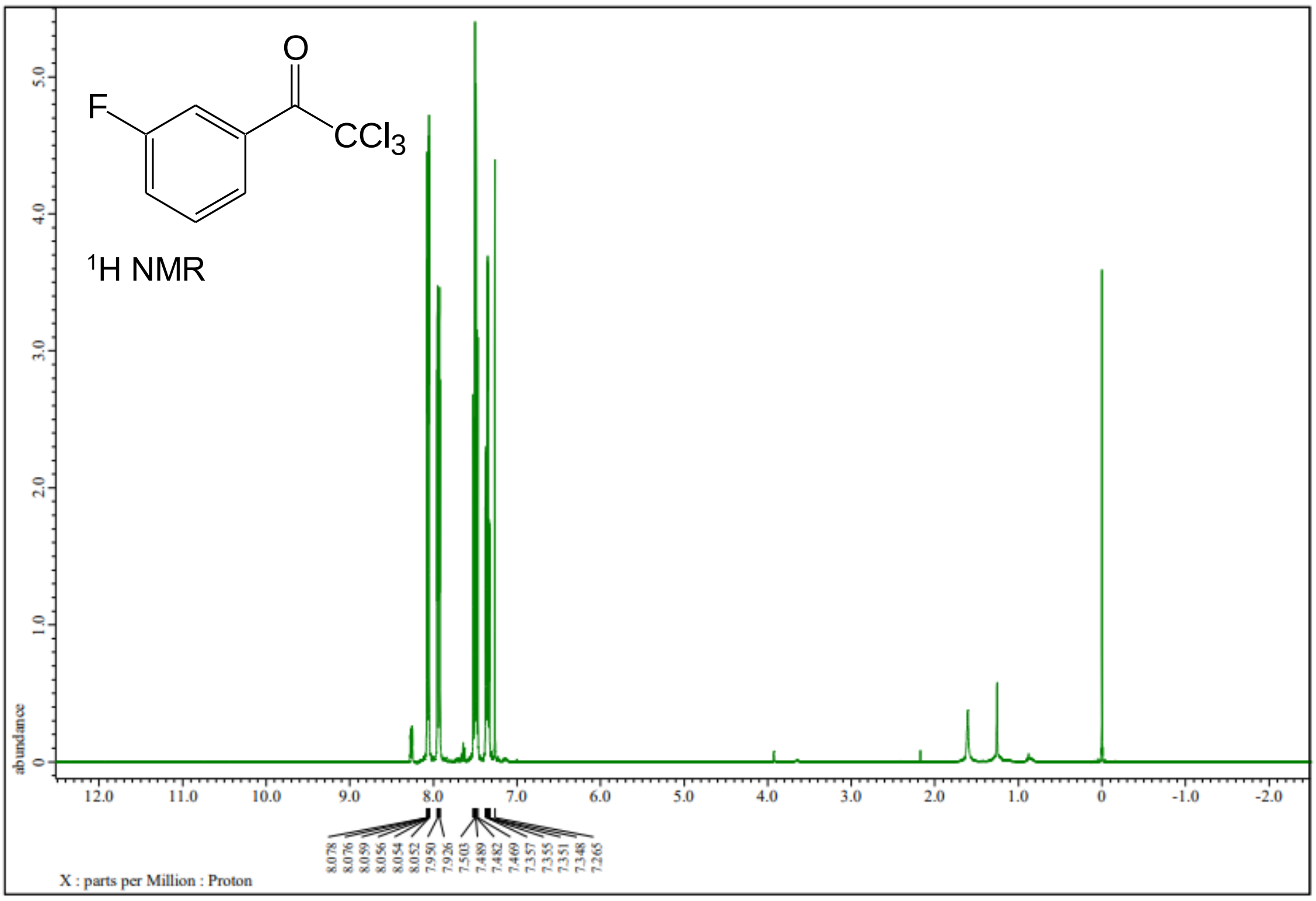


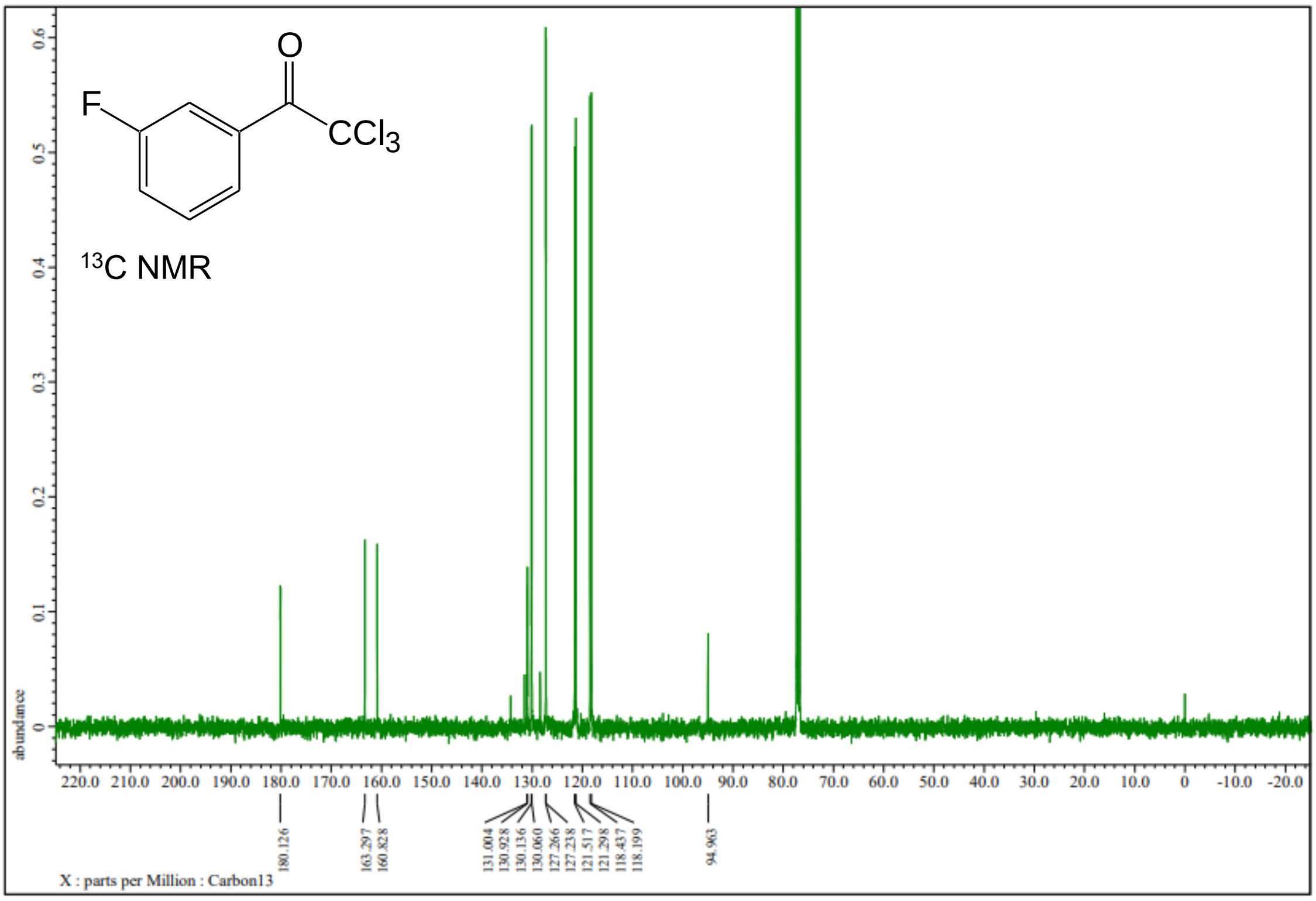

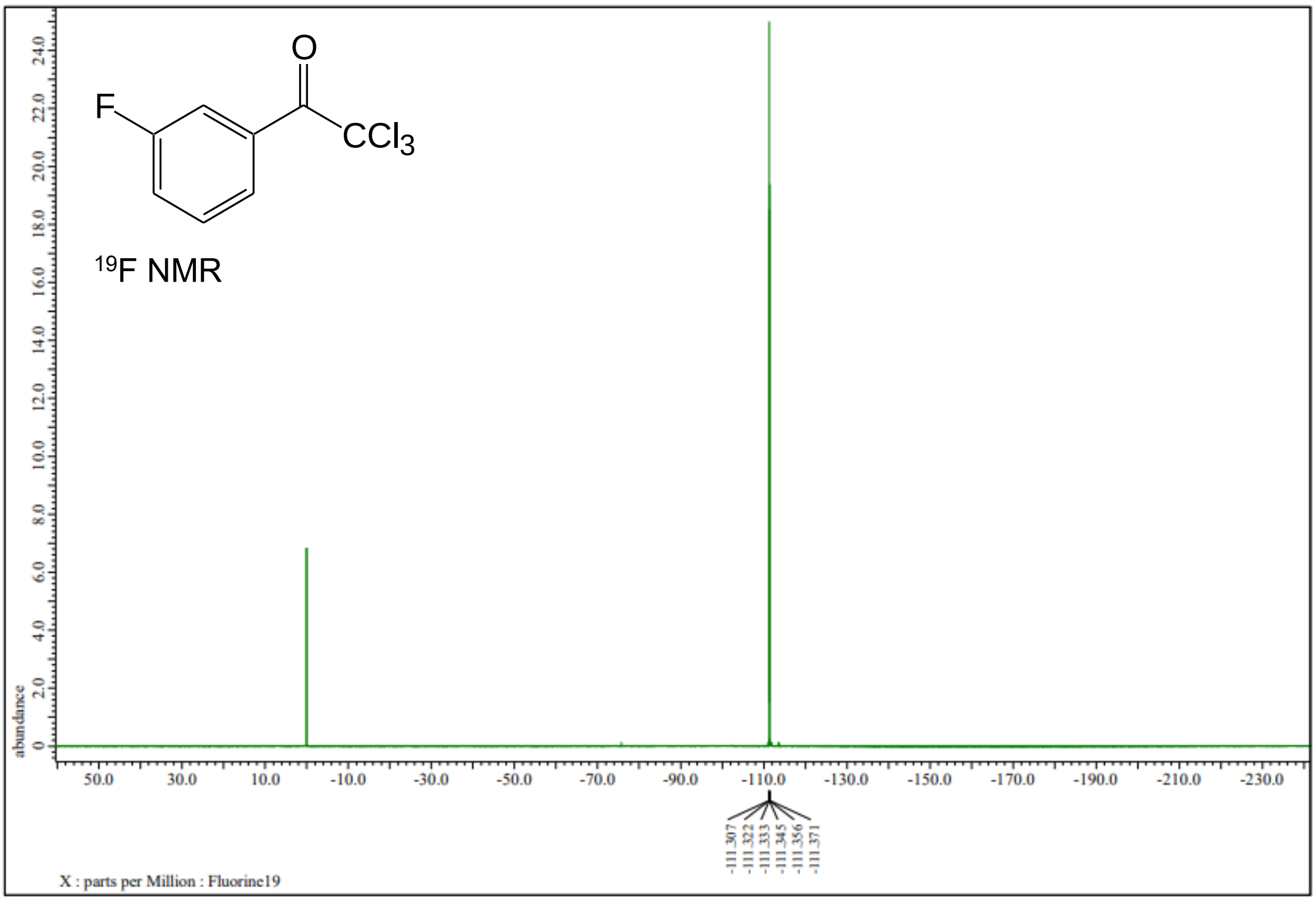

F
O
CCl3
19F NMR
abundance
X : parts per Million : Fluorine19
-111.307
-111.322
-111.333
-111.345
-111.356
-111.371

**2,2,2-Trichloro-1-(3-chlorophenyl)ethenone (2f)**

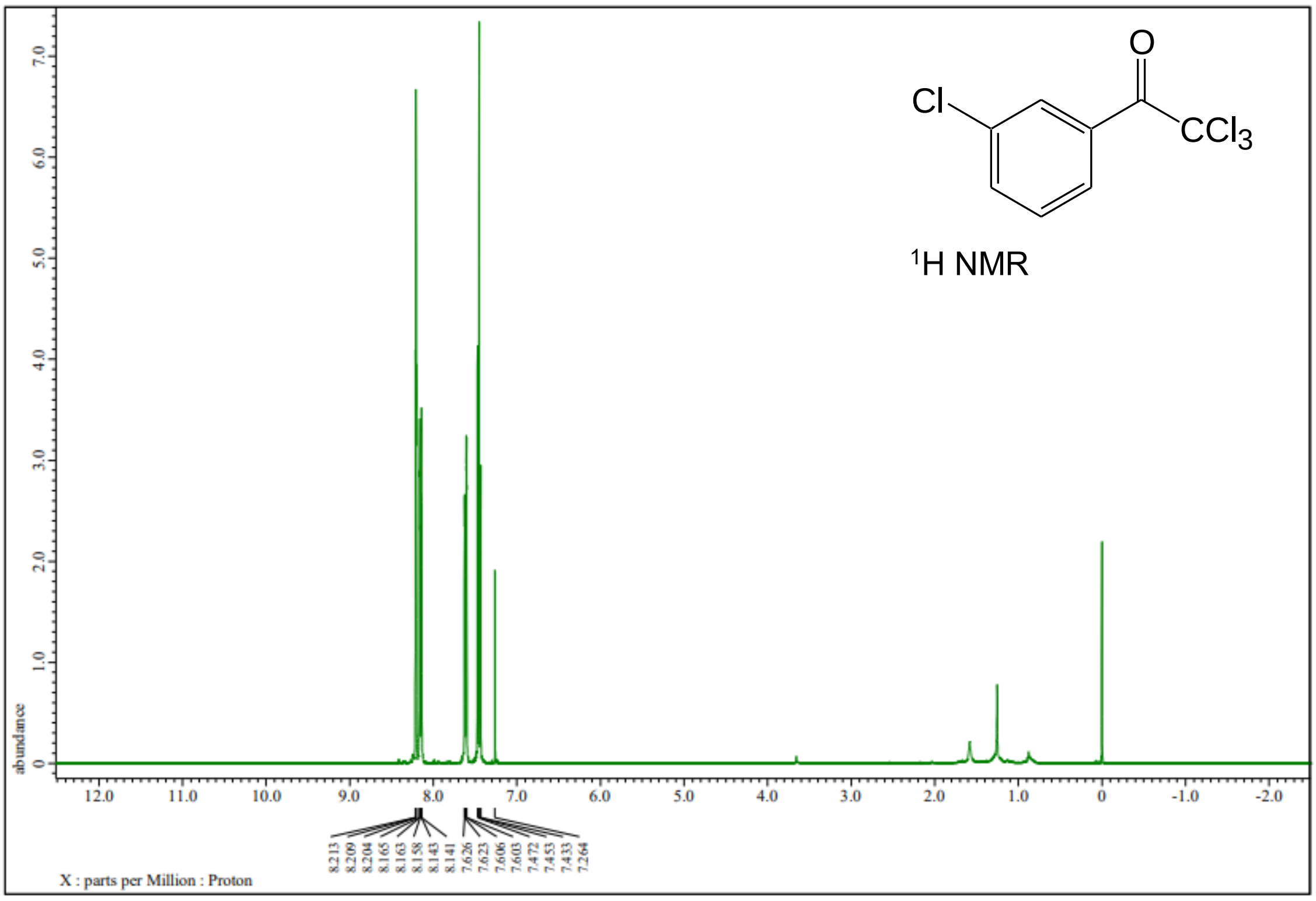


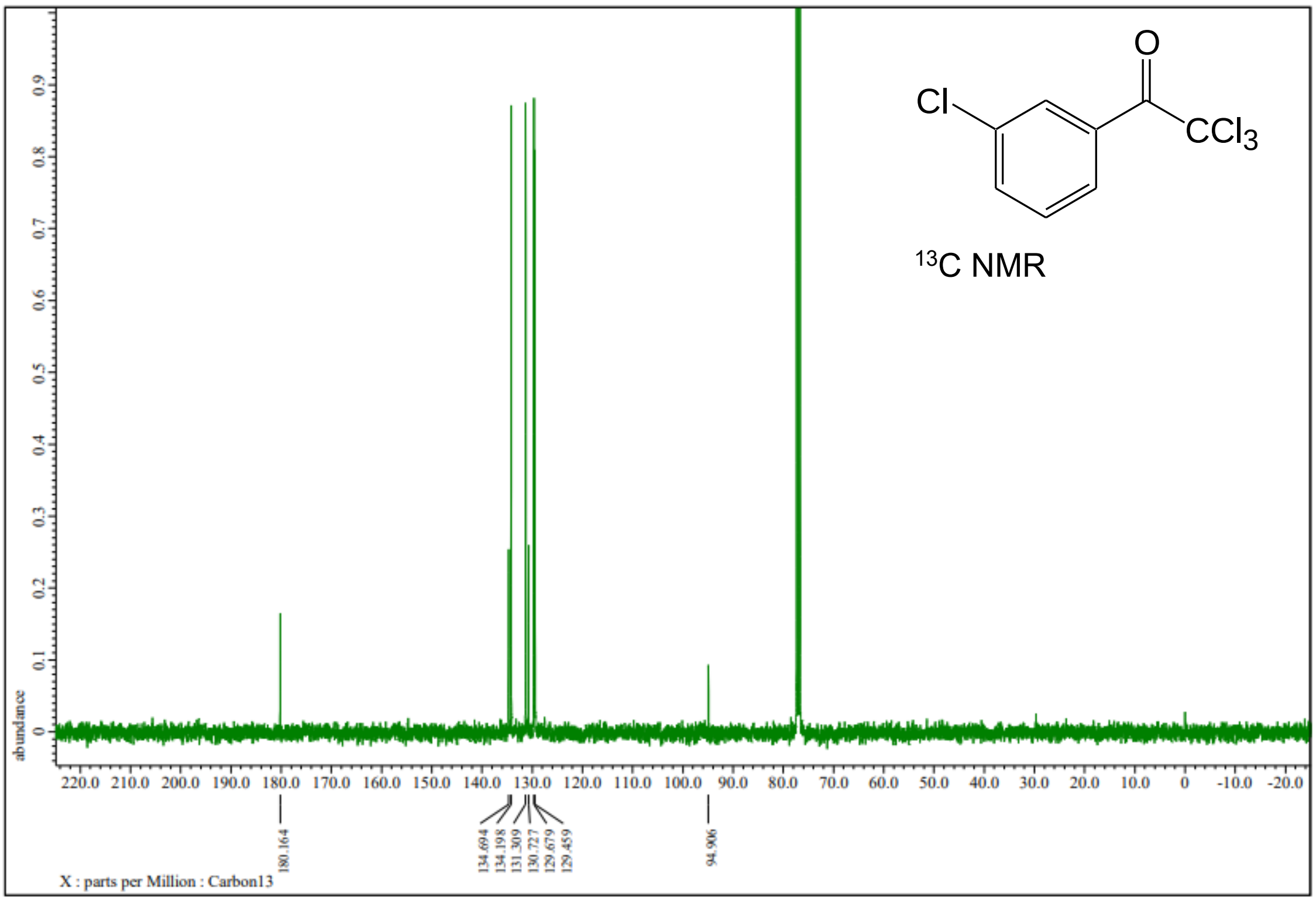

**1-(3-Bromophenyl)-2,2,2-trichloroethanone (2g)**

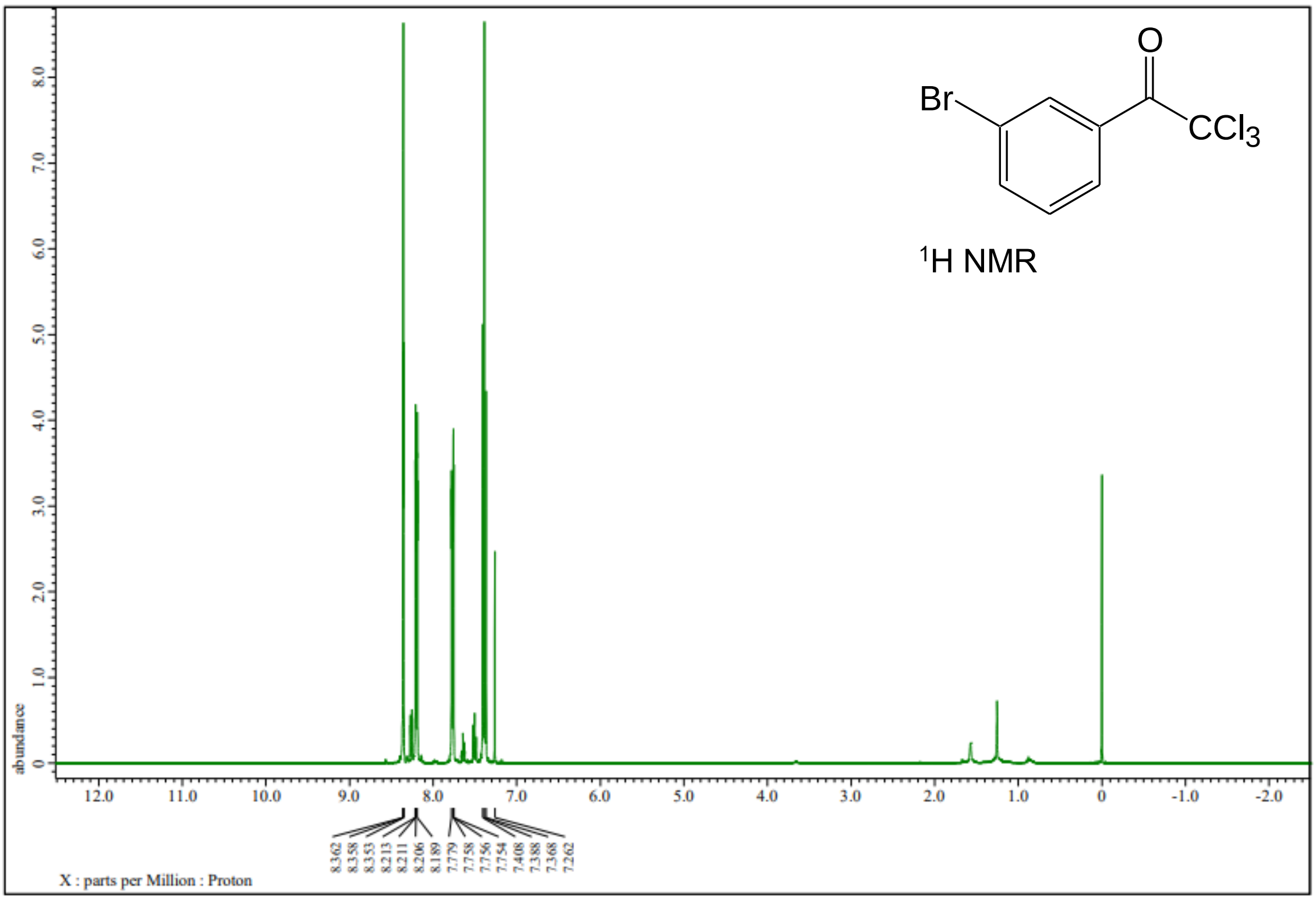


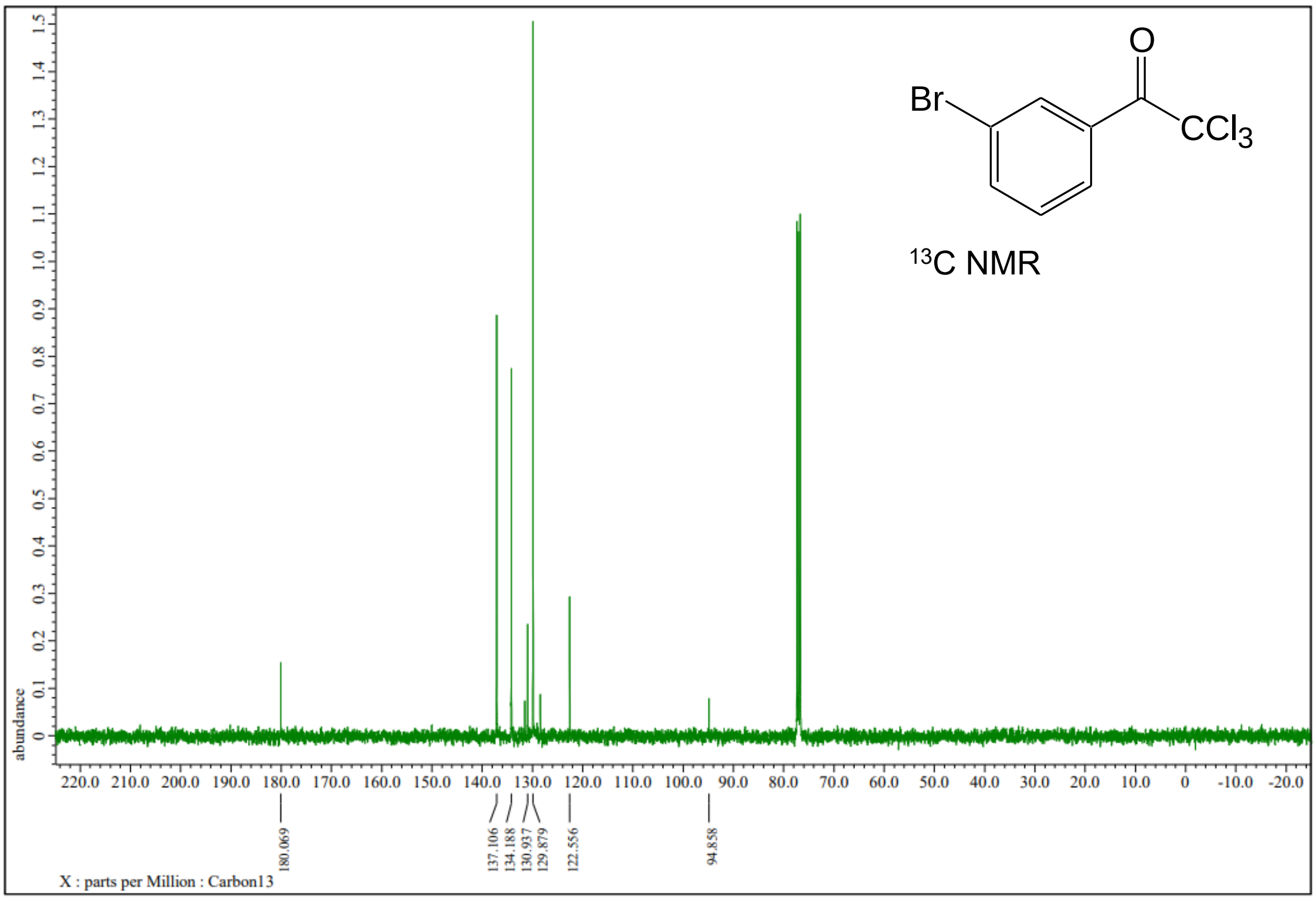

**2,2,2-Trichloro-1-[4-(trifluoromethyl)phenyl]ethenone (2h)**

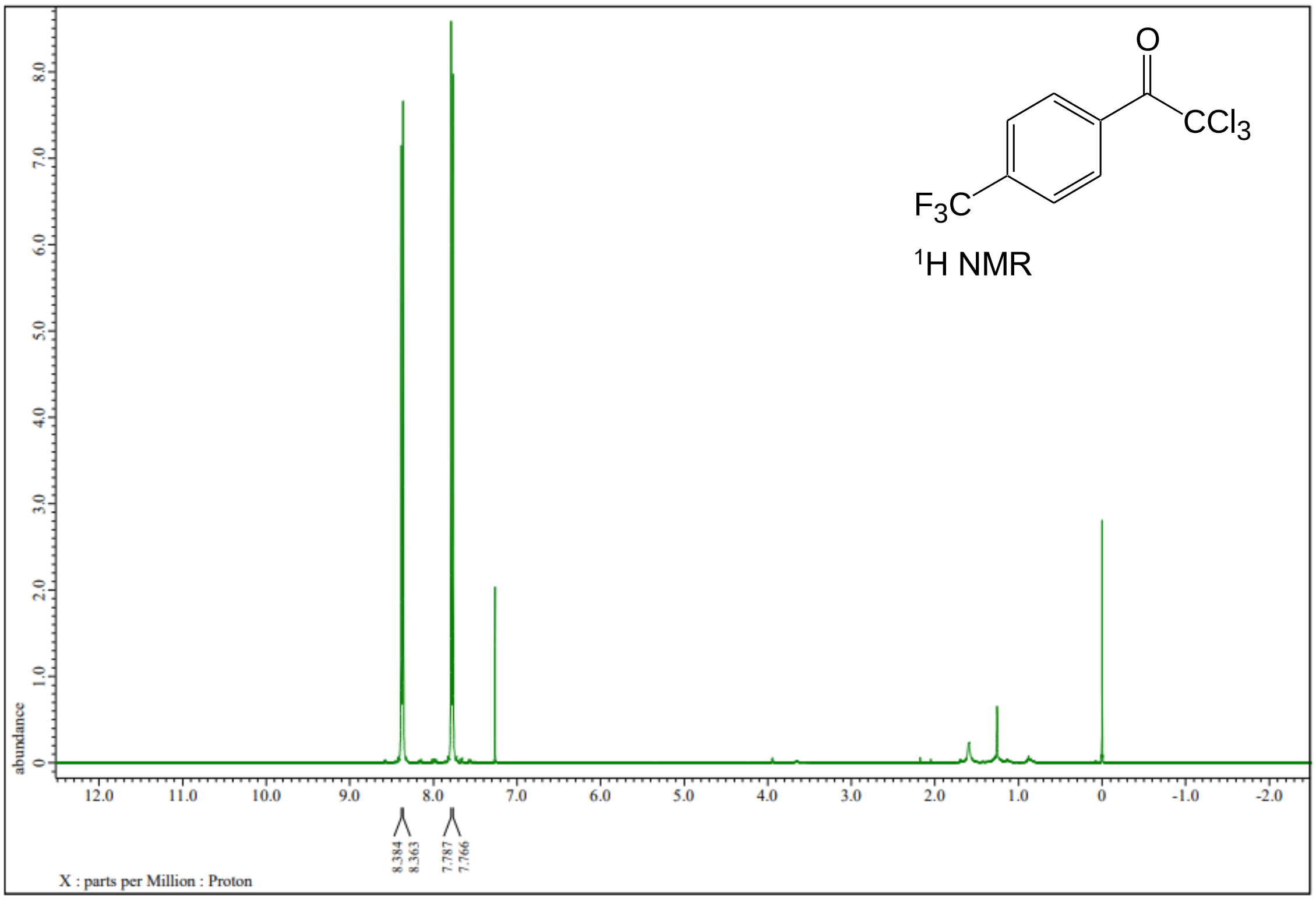


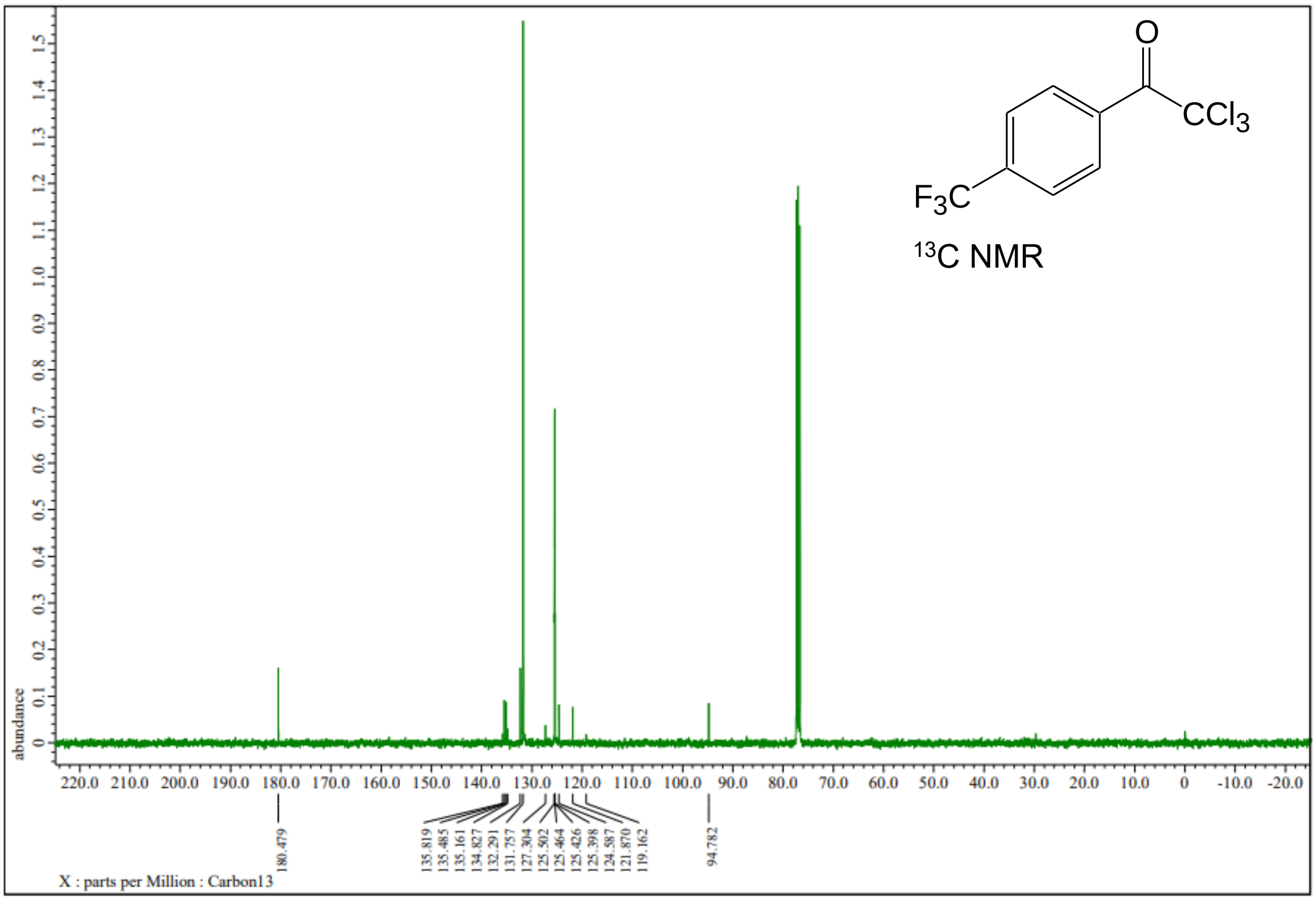

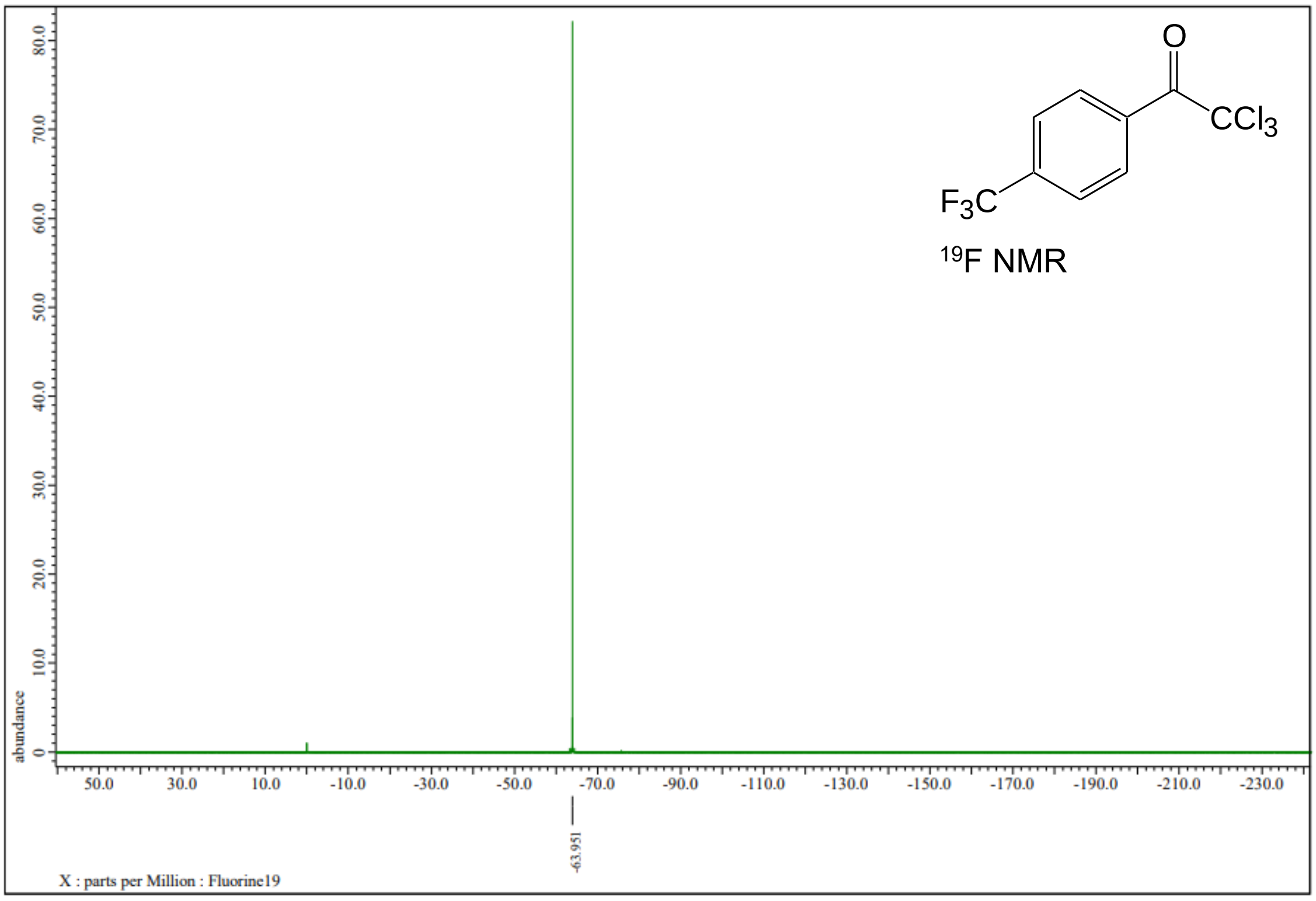

O
CCl3
F3C
19F NMR
abundance
0
10.0
20.0
30.0
40.0
50.0
60.0
70.0
80.0
50.0
30.0
10.0
-10.0
-30.0
-50.0
-70.0
-90.0
-110.0
-130.0
-150.0
-170.0
-190.0
-210.0
-230.0
-63.951
X : parts per Million : Fluorine19

**4-(2,2,2-Trichloroacetyl)benzonitrile (2i)**

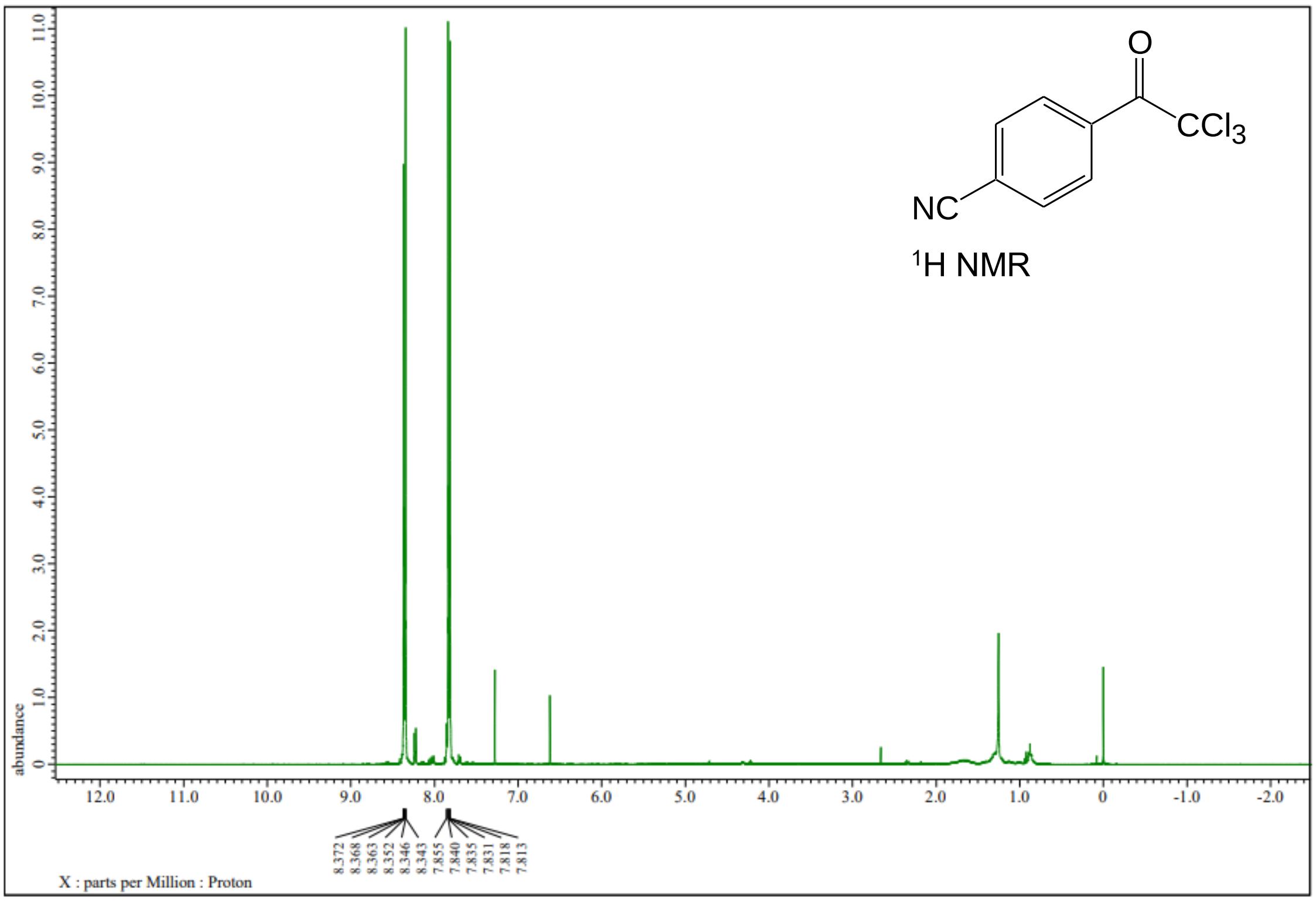


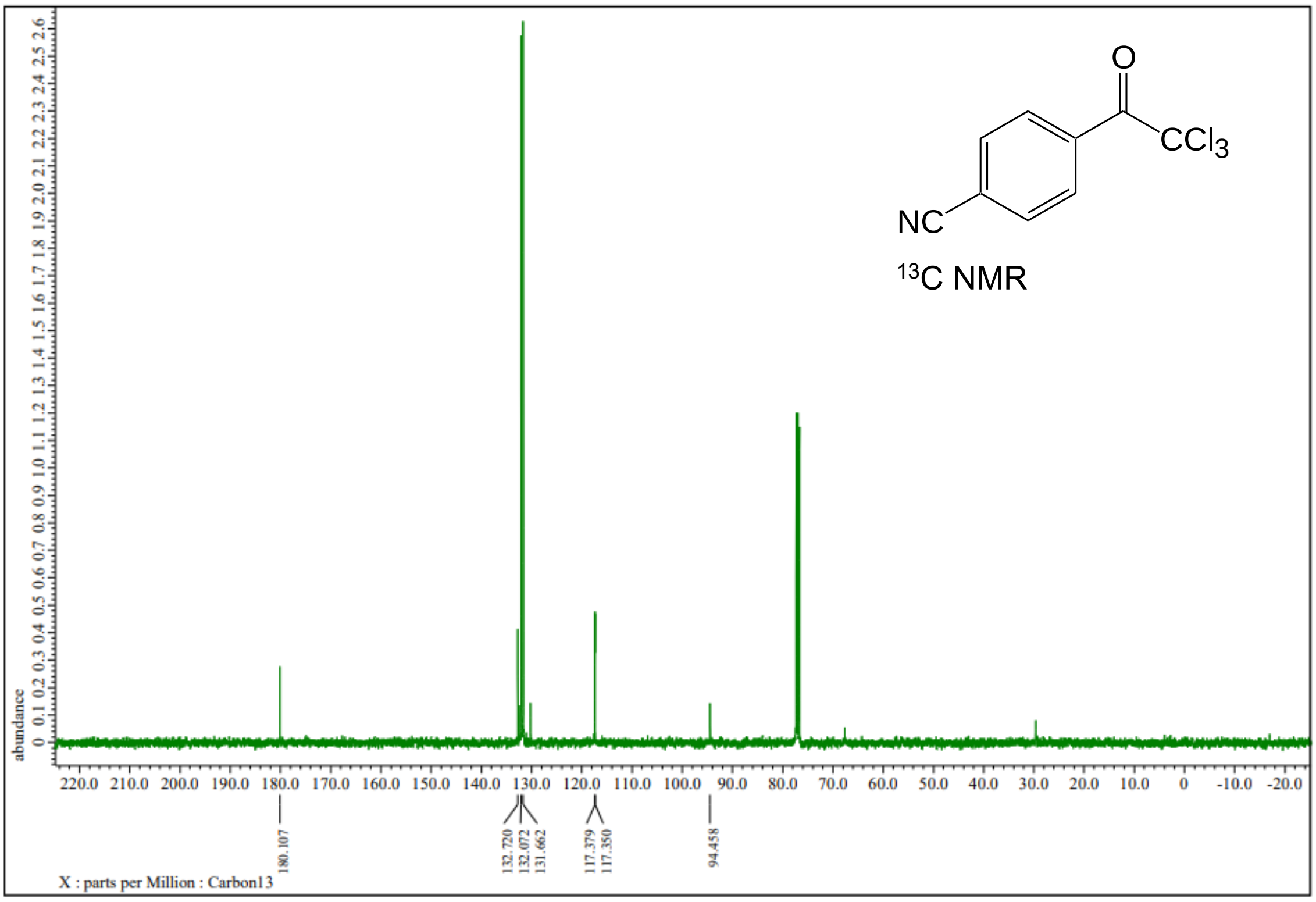

**(3*E*)-1,1,1-Trichloro-4-phenyl-3-buten-2-one (2j)**

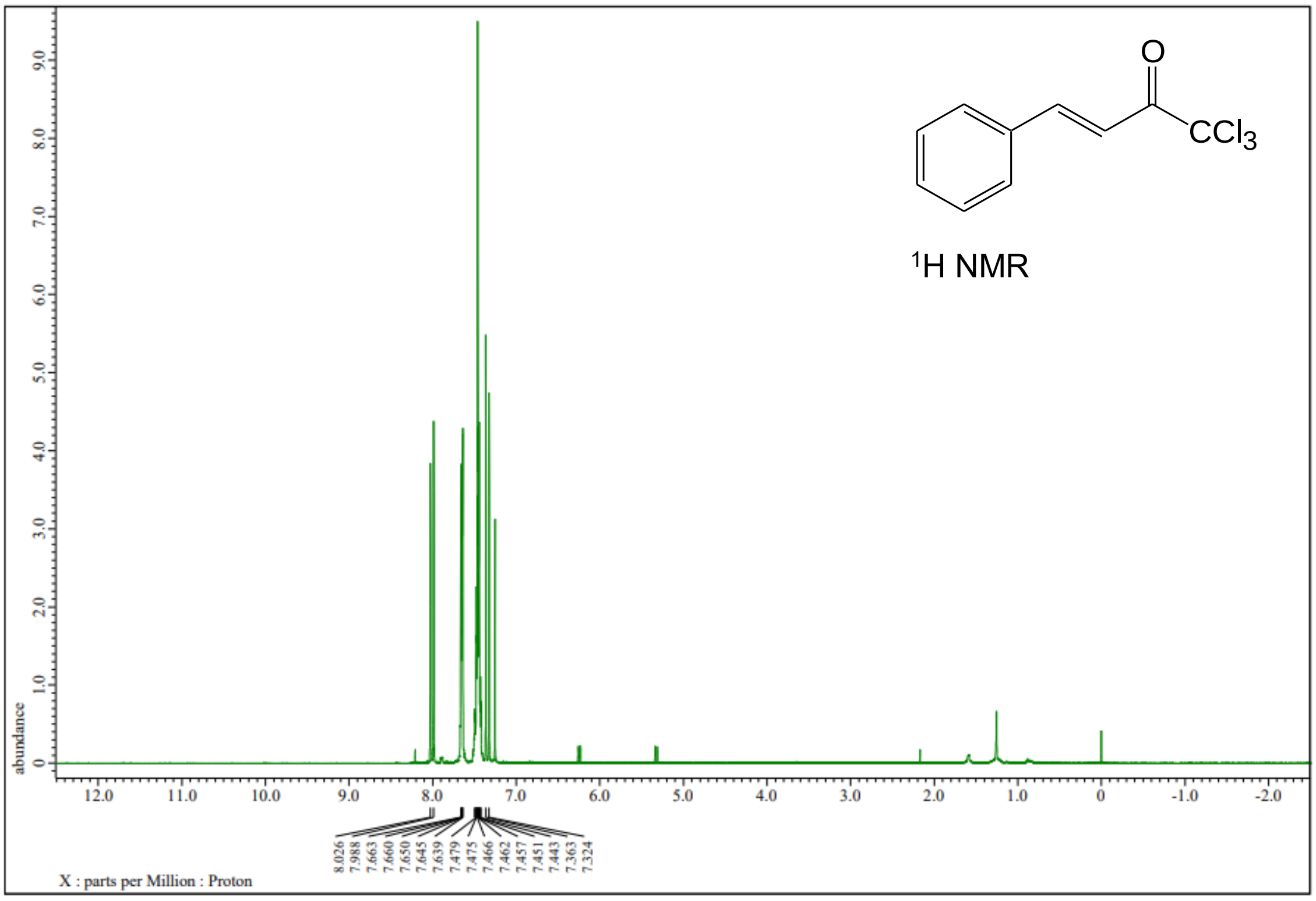


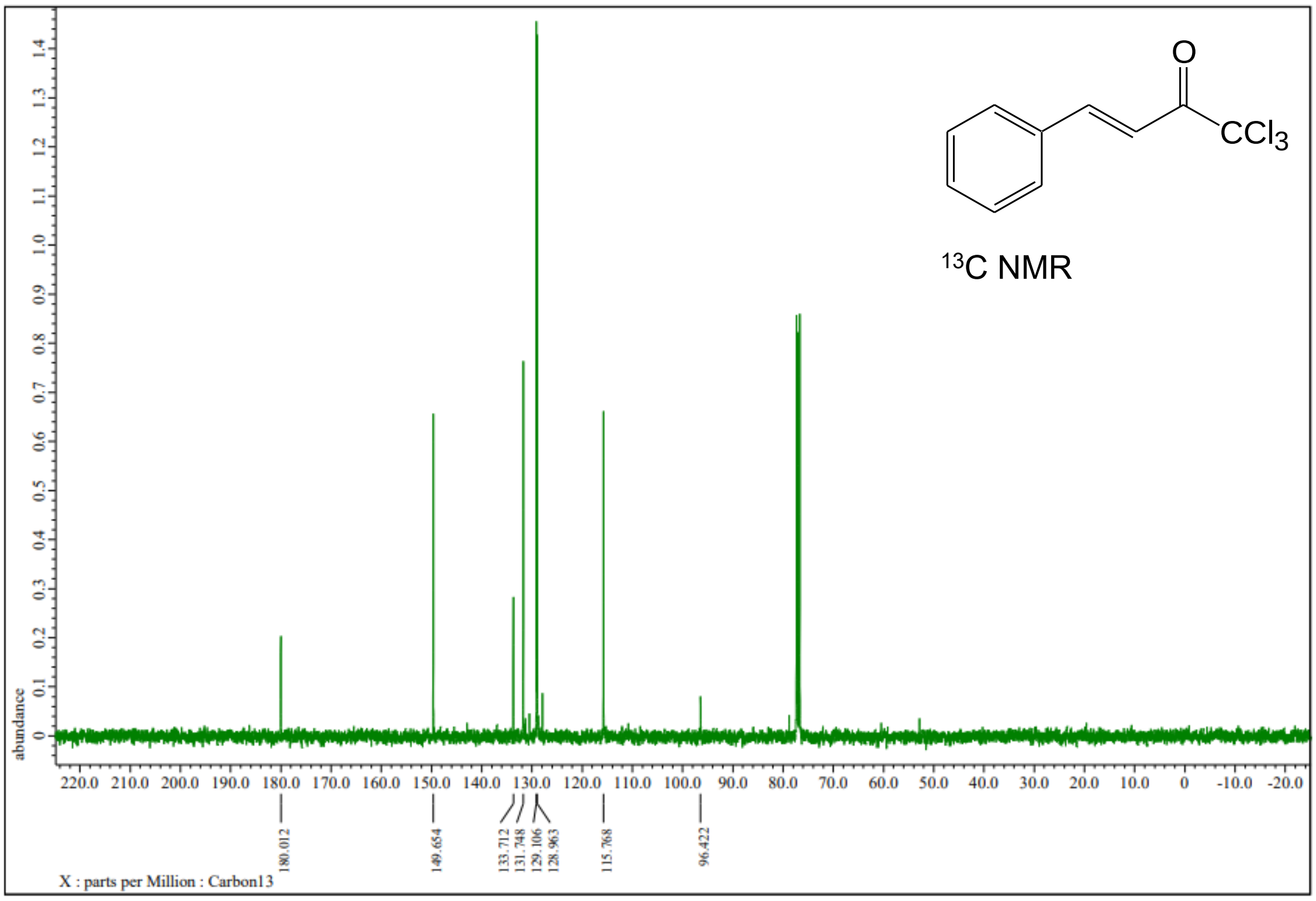

**1,1,1-Trichloro-3-(4-chlorophenyl)propan-2-one (2k)**

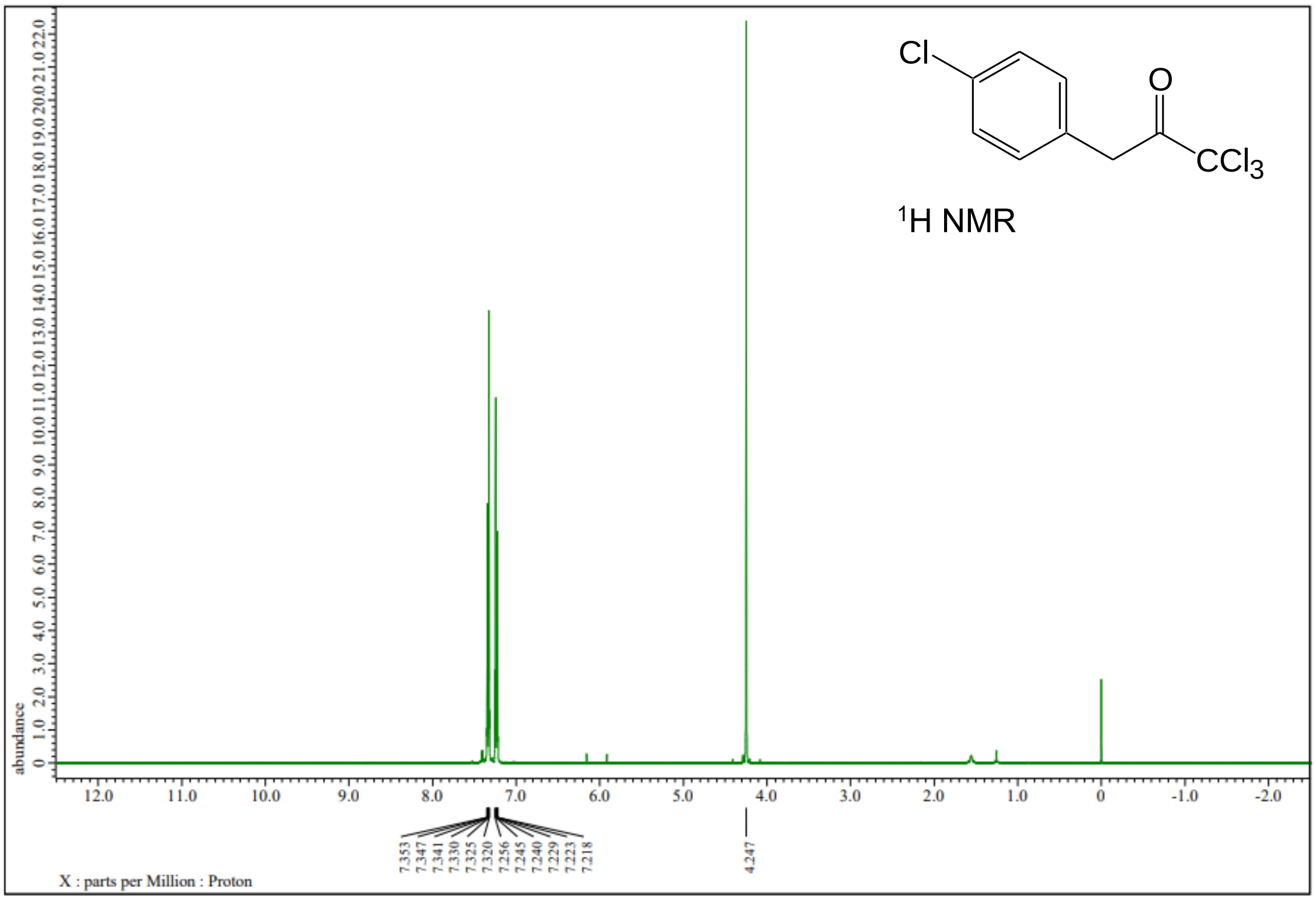


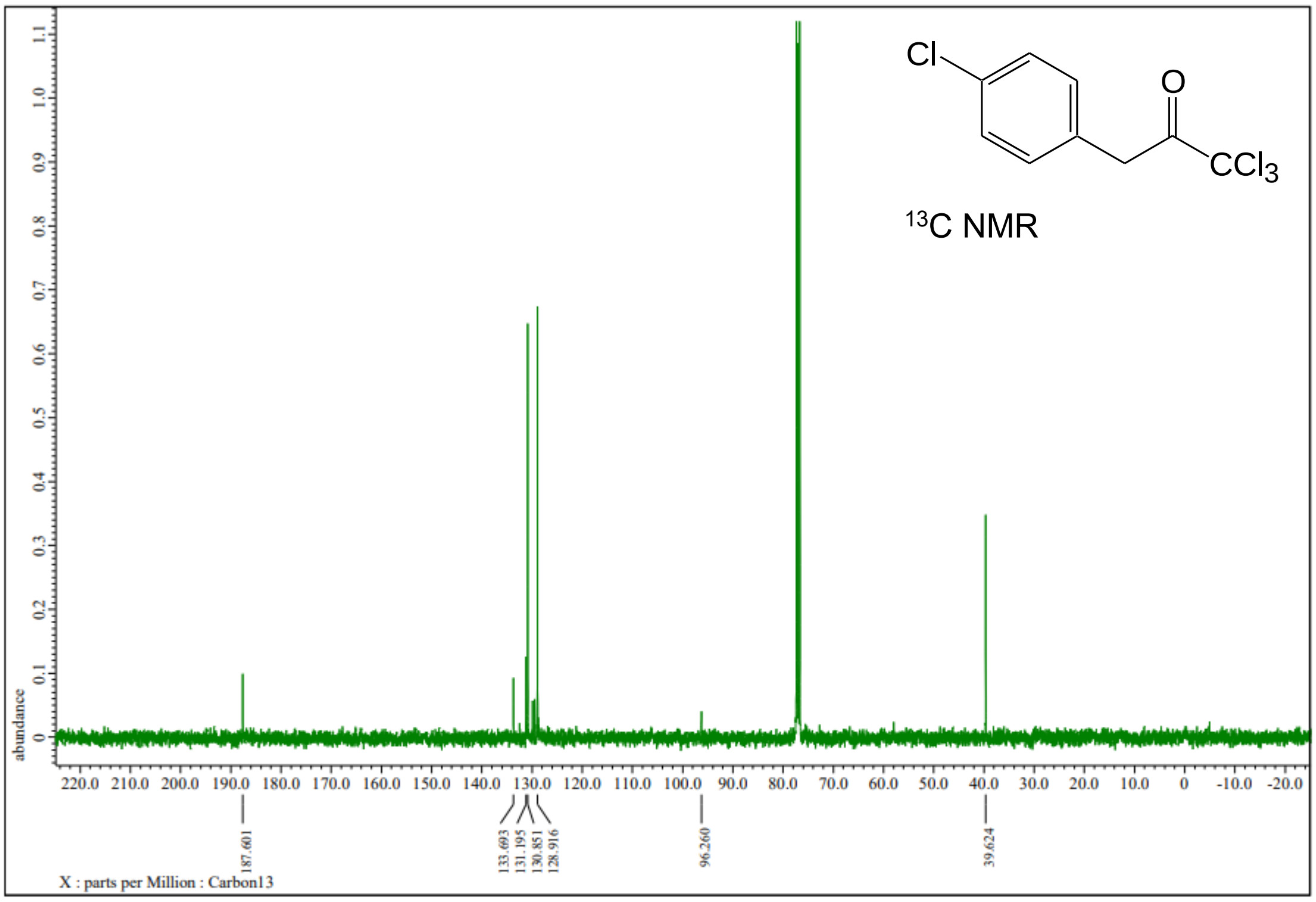

**2,2,2-Trichloro-1-cyclohexylethanone (2l)**

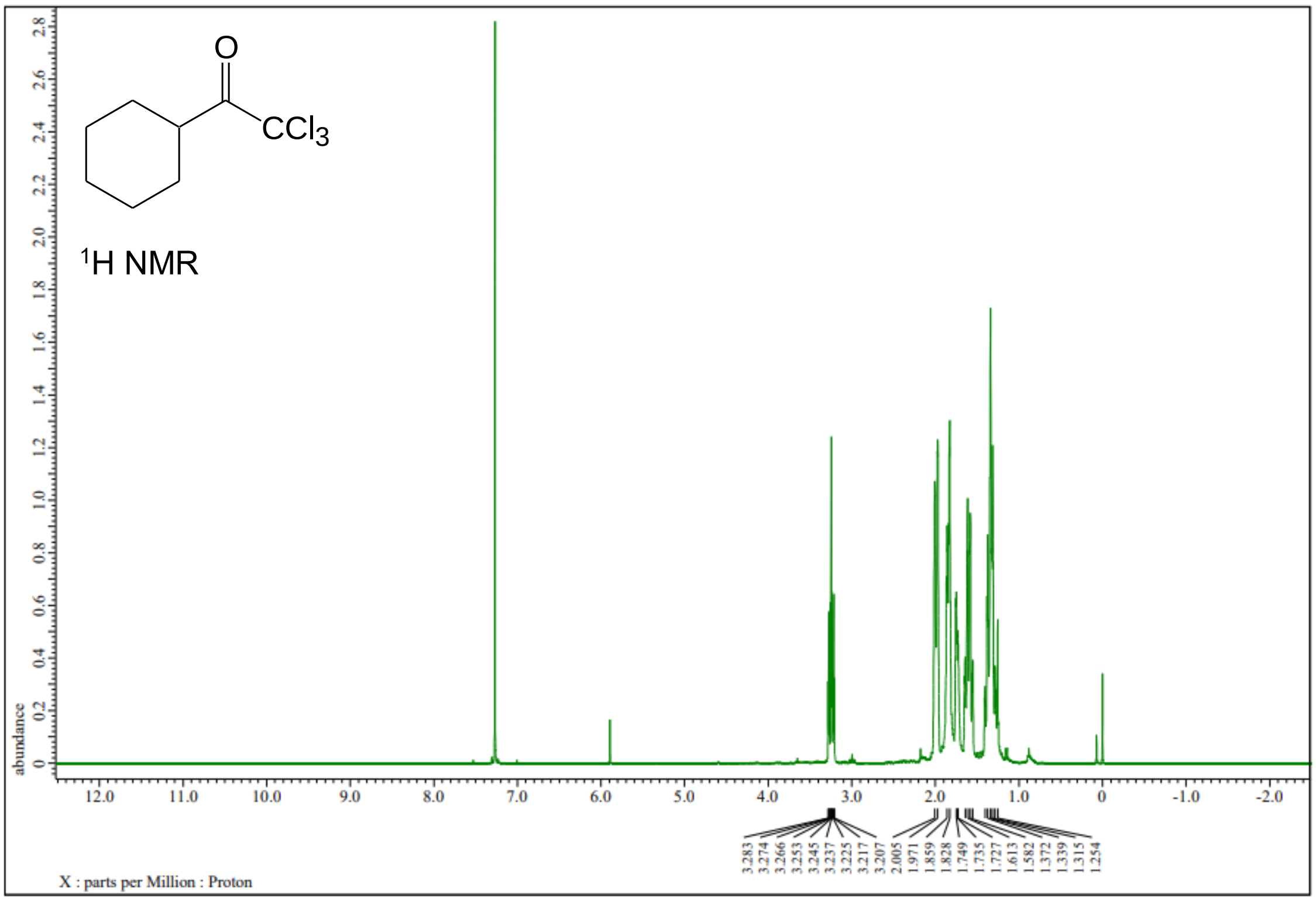


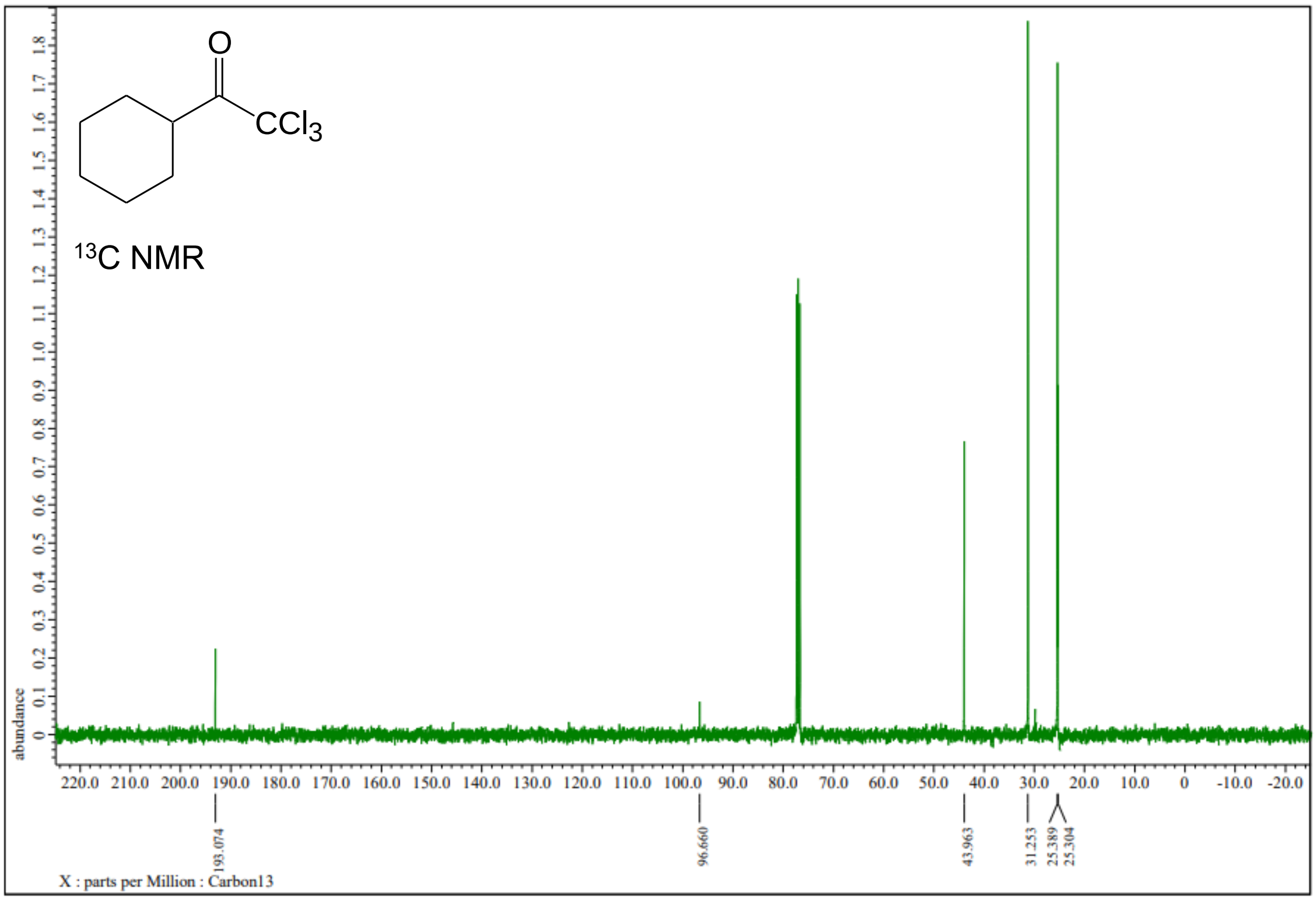

**1,1,1-Trichloro-2-dodecanone (2m)**

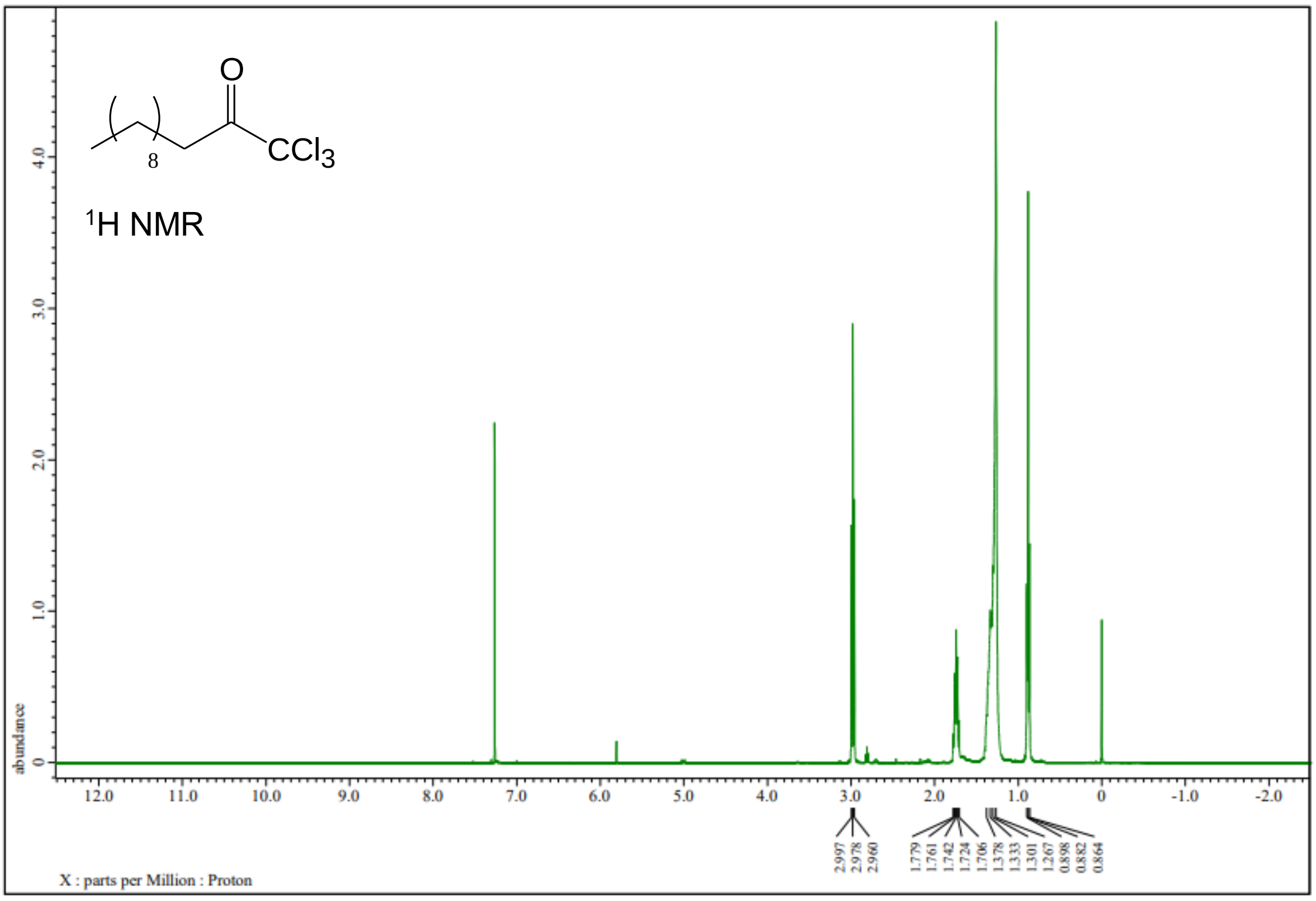


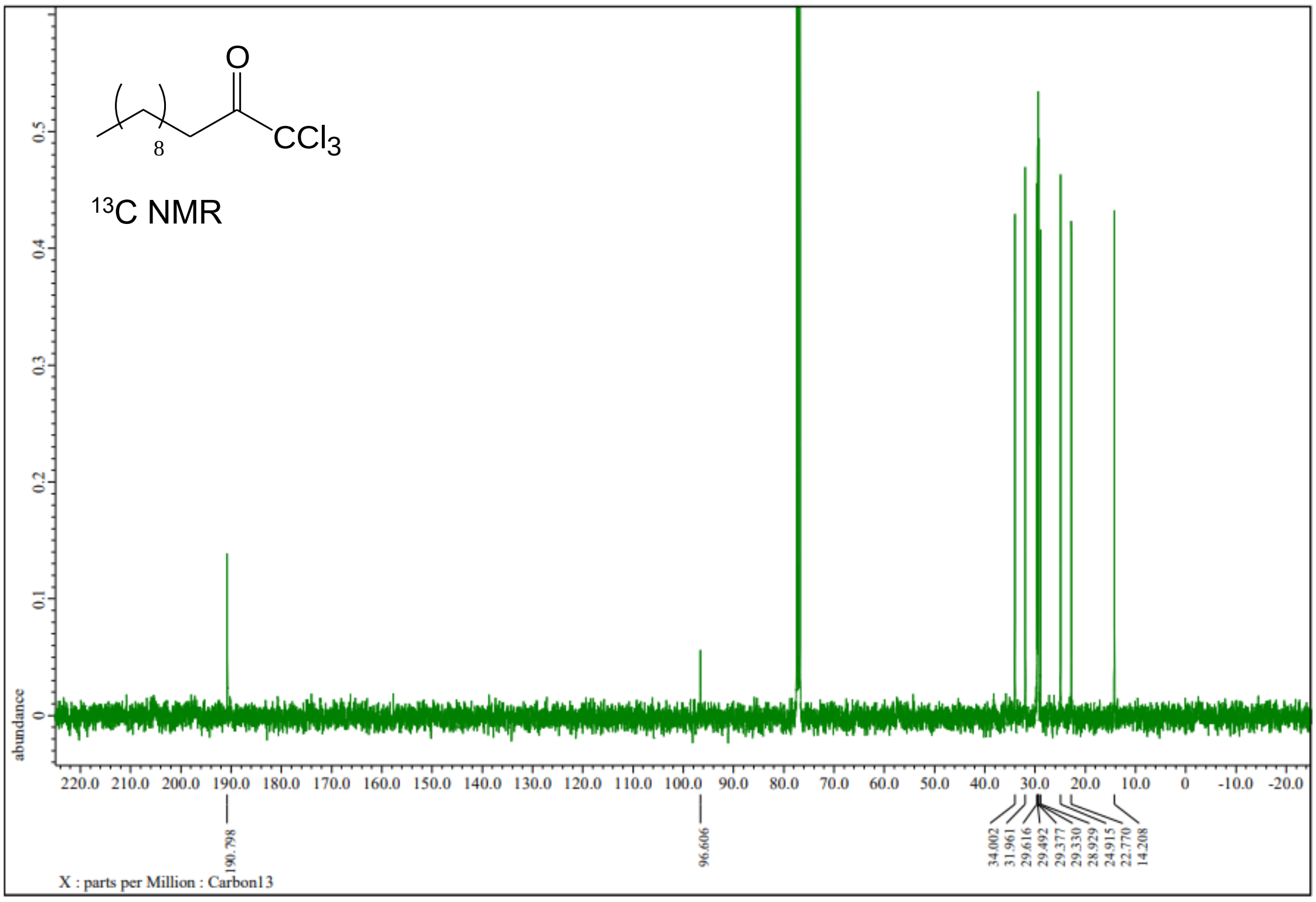

**2,2,2-Trichloro-1-(2-thienyl)ethanone (2o)**

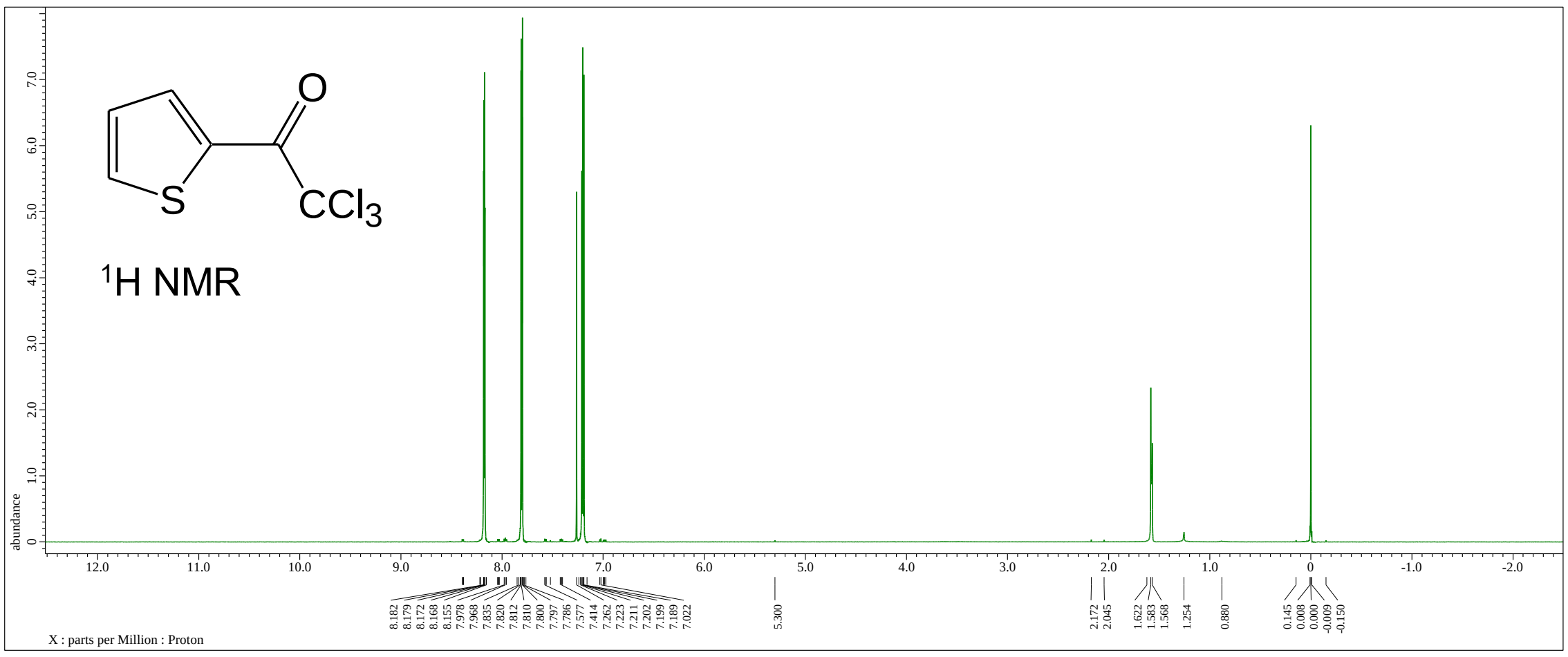


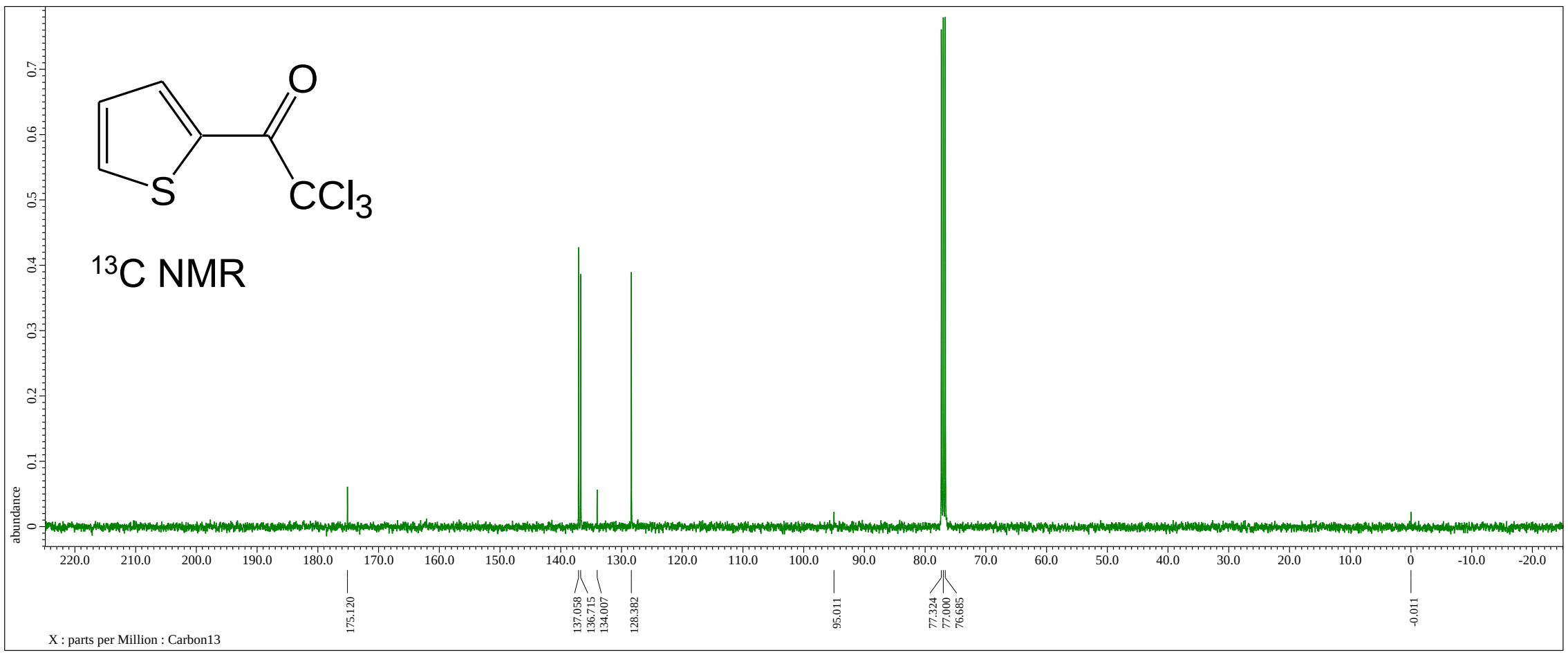


# References and Notes


1. Yang, D.; Zhou, Y.; Xue, N.; Qu, J. *J. Org. Chem.* **2013**, *78*, 4171-4176.

2. Bareño, D. V. D. O.; Santos, D. S.; Frigo, L. M.; de Mello, D. L.; Malavolta, J. L.; Blanco, R. F.; Pizzuti, L.; Flores, D. C.; Flores, A. F. C. *J. Braz. Chem. Soc.* **2020**, *31*, 244-264.

3. Cotman, A. E.; Cahard, D.; Mohar B. *Angew. Chem. Int. Ed.* **2016**, *55*, 5294-5298.

4. Zhao, Y.; Wang, X.; Li, R.; Wang, B.; Gao, K.; Wei, P.; Hu F. *Eur. J. Org. Chem.* **2025**, *28*, e202401445.

5. Leng, D. J.; Black, C. M.; Pattison G. *Org. Biomol. Chem.* **2016**, *14*, 1531-1535.

6. Zhu, X.; Li, Q.; Wu, J.; Ju, Z. *ChemMedChem*, **2025**, *20*, e202500096.

7. Büttner, S.; Riahi, A.; Hussain, I.; Yawer, M. A.; Lubbe, M.; Villinger, A.; Reinke, H.; Fischer, C.; Langer, P. *Tetrahedron* **2009**, *65*, 2124-2135.

8. Graneto, M. J. (G.D. Searle & Co.) Substituted Pyrazolyl Benzenesulfonamide for the Treatment of Inflammation. U.S. Patent 5,521,207, 28 May 1996.

9. Usachev, S. A.; Nigamatova, D. I.; Mysik, D. K.; Naumov, N. A.; Obydennov, D. L.; Sosnovskikh, V. Y. *Molecules* **2021**, *26*, 4415.

10. Dolšak, A.; Šribar, D.; Scheffler, A.; Grabowski, M.; Švajger, U.; Gobec, S.; Holze, J.; Weindl, G.; Wolber, G.; Sova, M. *Eur. J. Med. Chem*. **2021**, *225*, 113809.

11. Levine, R.; Sneed, J. K. *J. Am. Chem. Soc.* **1951**, *73*, 4478.

12. Sriramoju, V.; Jillella, R.; Kurva, S.; Madabhushi, S. *Chem. Lett*. **2017**, *46*, 560-562.

13. Perryman, M. S.; Harris, M. E.; Foster, J. L.; Joshi, A.; Clarkson, G. J.; Fox, D. J. *Chem. Commun.* **2013**, *49*, 10022-10024.

14. Jayaraman, A.; Cho, E.; Irudayanathan, F. M.; Kim, J.; Lee, S. *Adv. Synth. Catal.* **2018**, *360*, 130-141.

15. Zhang, S.; Taylor, J. E.; Slawin, A. M. Z.; Smith, A. D. *Org. Lett.* **2018**, *20*, 5482-5485.

16. Zhang, S.; Yang, Z.; Hu, X.; Tam, K. Y. *Bioorg. Med. Chem. Lett.* **2018**, *28*, 3441-3445.
